# A genome scale overexpression screen to reveal drug activity in human cells

**DOI:** 10.1186/gm549

**Published:** 2014-04-29

**Authors:** Anthony Arnoldo, Saranya Kittanakom, Lawrence E Heisler, Anthony B Mak, Andrey I Shukalyuk, Dax Torti, Jason Moffat, Guri Giaever, Corey Nislow

**Affiliations:** 1 Department of Molecular Genetics, University of Toronto, Toronto, M5S 3E1, Canada; 2 Banting and Best Department of Medical Research, University of Toronto, Toronto, M5S 3E1, Canada; 3 Terrence Donnelly Centre for Cellular and Biomolecular Research, University of Toronto, 160 College Street, Toronto, Ontario M5S 3E1, Canada; 4 Donnelly Sequencing Center, University of Toronto, 160 College Street, Toronto, Ontario M5S 3E1, Canada; 5 Institute of Biomaterials and Biomedical Engineering, University of Toronto, 170 College Street, Toronto M5S 3E3, Canada; 6 Department of Pharmaceutical Sciences, University of Toronto, 144 College Street, Toronto, Ontario M5S 3M2, Canada; 7 Department of Pharmaceutical Sciences, University of British Columbia, 6619-2405 Wesbrook Mall, Vancouver, BC V6T 1Z3, Canada

## Abstract

Target identification is a critical step in the lengthy and expensive process of drug development. Here, we describe a genome-wide screening platform that uses systematic overexpression of pooled human ORFs to understand drug mode-of-action and resistance mechanisms. We first calibrated our screen with the well-characterized drug methotrexate. We then identified new genes involved in the bioactivity of diverse drugs including antineoplastic agents and biologically active molecules. Finally, we focused on the transcription factor RHOXF2 whose overexpression conferred resistance to DNA damaging agents. This approach represents an orthogonal method for functional screening and, to our knowledge, has never been reported before.

## Background

Biological systems tend to remain phenotypically stable in the face of environmental challenges and genetic changes [1]. As such, genetic perturbation has become an efficient technique to dissect cellular functions. In this study, we used the concept of modulating gene dosage in human cells to gain insight into drug mode of action. Understanding the primary mechanism of action as well as the potential polypharmacological effects of a drug can provide insight into how to reduce detrimental side effects, uncover new applications for novel indication and explain resistance mechanisms [2-4].

Drug resistance in malignant tissues can be categorized into three main mechanisms: (i) drug distribution/metabolism (pharmacokinetics), (ii) heterogeneity of cancer cells and (iii) tumor micro-environment [5,6]. Among the other cellular mechanisms, gene overexpression (for example, by amplification of the drug target) can titrate a drug’s effect. This is exemplified by the classic case of methotrexate resistance through the amplification of the gene *DHFR* in neoplastic tissue from an individual with disseminated small-cell lung cancer that relapsed during methotrexate chemotherapy [7]. Other forms of overexpression resistance include the up-regulation of 1) efflux pumps (for example, ATP-binding cassette transporters), 2) survival mechanisms (for example, anti-apoptotic proteins), 3) DNA damage repair, 4) pathways for drug inactivation and 5) the overexpression of target isotypes. Additionally, proteins downstream of the inhibited target can be modulated in such a manner as to bypass the toxic effect of a drug.

To dissect some of these mechanisms by which drugs act within the cell, we postulated that, when overexpressed, genes conferring resistance to a lethal chemical treatment can illuminate the drug mode of action. Overexpressing genes to confer resistance to an otherwise toxic compound is a well-established concept. Early studies showed that a streptomycin resistance gene cassette could function in a bacterial tetracycline resistant plasmid [8] and it was shown in other studies that cloning of the mouse dihydrofolate reductase into a bacterial plasmid provided resistance to trimethoprim in *Escherichia coli*[9]. A large-scale approach using the overexpression of a yeast genomic DNA library to identify genes conferring resistance to specific drugs has validated the concept of gene dosage for the discovery of drug targets in eukaryotes [10].

A logical extension of this concept is to employ newly available biological tools that enable the systematic perturbation of all protein function in human cultured cells using RNA interference (RNAi), CRISPR (clustered regularly interspaced short palindromic repeats), cDNA libraries, transposons or small molecule inhibitors in combination with gene overexpression. Although testing all important therapeutic drugs against all proteins from the approximately 20,000 genes of the human genome in all differentiated cell types is not currently feasible, we report the initial development of a gain-of-function approach to identify drug mode of action by the overexpression of 12,200 human ORFs (hORFs) [11,12] in the human cell line HEK293.

In summary, we have developed a new experimental pipeline to identify genes whose up-regulation suppresses the toxic effect of chemicals in human cells and have successfully applied this strategy to seven pharmacological compounds.

## Methods

### Cell culture and plasmids

Human embryonic kidney HEK293 (ATCC), HEK293T (ATCC), non-small cell lung carcinoma NCI-H1299 (ATCC), chondrosarcoma SW1353 (gift from Johanne Martel-Pelletier, University of Montreal, Canada) and pancreatic adenocarcinoma HPAC (ATCC) cells were maintained in DMEM (Wisent Inc. Montreal, Quebec, Canada) supplemented with 10% fetal bovine serum (Gibco, Carlsbad, California, US) at 37°C and 5% CO_2_. The rtTA expressing HEK293_M2 (ATCC), human breast adenocarcinoma MCF7_M2 (ATCC) and adenocarcinomic human alveolar basal epithelial A549_M2 (ATCC) cells were maintained in DMEM (Wisent Inc.) supplemented with 10% fetal bovine serum (Gibco) at 37°C and 5% CO_2_. Blast phase chronic myelogenous leukemia K562 cells (gift from Reinhart Reithmeier, University of Toronto, Canada) were maintained in RPMI1640 (Sigma, St. Louis, Missouri, US) supplemented with 10% fetal bovine serum (Gibco) at 37°C and 5% CO_2_. Multiple myeloma U266B1 cells (gift from Aaron Schimmer, Princess Margaret Cancer Centre, Canada) were maintained in Iscove's modified Dulbecco's medium (IMDM; Gibco) supplemented with 10% fetal bovine serum (Gibco) at 37°C and 5% CO_2_. Thyroid gland medullary carcinoma TT cells (gift from Gilbert Cote, MD Anderson Cancer Center, USA) were maintained in Ham’s F-12 K (Gibco) supplemented with 10% fetal bovine serum (Gibco) at 37°C and 5% CO_2_.

The lentiviral destination vector pLD-T-IRES-Venus-WPRE-STOP was cloned using the following procedure: the Gateway cassette from pLS-Dest-EcF (gift of Dr Tony Pawson, University of Toronto, Canada) was subcloned into pLJM17 (J Moffat, unpublished) using the restriction enzymes MluI and XbaI, resulting in the pLD-puro-TRE (T) vector. IRES-Venus-WPRE were PCR amplified from the pSLIK-Venus [13] using the following primers: XcmI_IRES_F (5′-GCGCCTTTTCCAAGGCAGCCCTGGAATTCCGCCCCTCTCCCTCC) and NsiI_WPRE_R (5′-AAACAATGCATGTCGACGCGGGGAGGCGGCCCAAAGGGAGATCC). The IRES-Venus-WPRE amplicon was cloned into the pLD-puro-T vector using the restriction enzymes XcmI and NsiI to replace the human phosphoglycerate kinase promoter and puromycin resistance gene. A stop codon was introduced directly downstream of the Gateway cassette by digesting pLD-T-IRES-Venus-WPRE with the restriction enzyme XbaI and blunted using DNA polymerase I, large (Klenow) fragment (New England Biolabs Inc., Beverly, Massachusetts, US).

The *piggyBac* PB-TGcMV-Neo plasmid used for the hit confirmation was derived from the PB-TET (AddGene, Cambridge, Massachusetts, US). The 'IRES-beta-Geo' fragment from PB-TET was replaced by the 'promoter PGK-neomycin resistance gene' fragment by homologous recombination in *Saccharomyces cerevisiae*. Briefly, PB-TET was digested with RsrII and ApaI to remove the beta-geo gene. The fragment coding for the promoter PGK and neomycin resistance gene, the URA3 cassette and the yeast 2 μ-origin of replication were PCR amplified and cloned into the digested PB-TET plasmid by homologous recombination in yeast. Yeast was then transformed with the p414-Cre plasmid in order to excise the URA3 cassette and the 2 μ-origin of replication after expression of the Cre recombinase.

All short hairpin RNAs (shRNAs) against the RHOXF2 and green fluorescent protein (GFP) mRNAs were derived from the RNAi Consortium (TRC) lentiviral libraries and obtained in the pLKO.1 vector. Five shRNAs against RHOXF2 were tested and were designed against the following regions: 5′-CGGGATGAGAGATGATTACTT (RHOXF2_shRNA_1); 5′-GACGAGAAAGAACTACAGGAT (RHOXF2_shRNA_2); 5′-AGAAGCATGAATGTGACTGAA (RHOXF2_shRNA_3); 5′-GAGGGCATTAATGGCAAGAAA (RHOXF2_shRNA_4); 5′-GTCGCTTACTGAAGAGGTCAA (RHOXF2_shRNA_5).

### Library preparation, virus production and lentiviral infection

The 12,212 hORF collection (representing 10,214 distinct genes) represents version 3.1 of an ongoing effort to create a complete human set of protein-encoding genes [11,12]. Initially based on the Mammalian Gene Collection cDNA collection, that work transfers full-length ORFs (excluding the 5′ and 3′ mRNA untranslated regions) into a Gateway system. Version 3.1 was obtained from Open Biosystems (Thermo Scientific, Waltham, Massachusetts, US) and regroups non-fully sequenced verified and non-clonal ORFs (limitations corrected in the 8.1 version [14]). The collection was divided into 34 minipools of 376 hORFs; for each minipool, the hORFs were cloned *en masse* from the pDONR223 into the lentiviral expression vector pLD-T-IRES-Venus-WPRE-STOP by Gateway LR reaction. After electroporation, transformants were selected on Luria Broth (LB) plus ampicillin and the lentiviral vector was extracted.

Lentivirus was produced by normalizing the amount of DNA for the 34 hORF minipools and by co-transfection with the packaging plasmid psPAX2 and the envelope plasmid pMD2.G into the packaging HEK293T cells using FuGENE (Roche Mississauga, Ontario, Canada).

HEK293_M2 cells were then infected at a multiplicity of infection of 0.3. Doxycycline (2 μg/ml) was added to induce the expression of the Venus fluorescent protein and Venus-positive cells were sorted using a BD FACS Aria Cell Sorter (East Ruherford, New Jersey, US). Because the expression of the gene coding for Venus is linked to the expression of the hORFs, selection of the fluorescent cells allowed the selection for non-silent, stably integrated lentivirus as well as functional inducible hORFs.

### Genomic DNA extraction, library preparation and data analysis for the hORFeome representation in HEK293_M2 cells

We thawed 6.3 × 10^6^ HEK293_M2 cells harboring the virally integrated human ORFeome version 3.1 collection and these were cultured for 1 day to recover. Genomic DNA was recovered from 16 × 10^6^ cells and 6 μg of genomic DNA (gDNA were used to amplify the collection (three PCR reactions with 2 μg of gDNA). Following PCR, the amplified ORFs were used directly to prepare a Nextera sequencing library (Illumina Nextera DNA Sample Preparation Kit, San Diego, California, US) and sequenced on an Illumina MiSeq according to the manufacturer’s protocol. Fifty-nucleotide paired-end reads were aligned against a reference database consisting of the ORF sequences in the hORFeome 3.1 collection using bwa (version 0.6.1). Coverage at each base position in each reference sequence was determined using the bedtools program genomeCoverageBed (v2.14.2). A custom Perl script was used to summarize the coverage levels to determine the mean coverage for each reference sequence and the proportion of each reference sequence with different levels of coverage. Raw data have been deposited in the ArrayExpress repository under accession number E-MTAB-2498.

### hORFeome drug target screen

Aliquots of 500,000 HEK293_M2 cells were seeded in a T175 flask and grown for 15 hours before the induction of hORF expression by addition of doxycycline. After 18 hours of induction, cells formed micro-colonies that were cultured in the presence of drug or DMSO (as control) for 2 to 3 weeks until distinct colonies were detectable.

After each screen, surviving cells were harvested and gDNA was extracted as follows. Cells were lysed using SNET buffer (10 mM Tris pH 8.0, 0.1 M EDTA, 0.5% SDS, 0.1 mg/ml Proteinase K, 25 μg/ml RNAse A). gDNA was isolated using one volume of phenol/chloroform/isoamyl alcohol (25:24:1) pH 8.0, precipitated by adding two volumes of 95% ethanol (-20°C), washed with ice cold 70% ethanol and resuspended in Tris-HCl pH 8.0.

During the screen selection experiments, only a minority of hORFs protected HEK293_M2s against the cytotoxic effect of the drug. Compared to the initial cell population, HEK293_M2 cells harboring the most resistant genes should be over-represented. In order to further amplify the set of resistant genes from the resulting reduced population, hORFs were PCR amplified from only 80 ng of genomic DNA. The sequences of the forward and reverse primers specific for the inserted hORFs were 5′-CGGTACCCGGGGATCCTCTAGTCAGCTGAC and 5′-CCATTTGTCTCGAGGTCGAGAATTCTAGCTAGAATC, respectively. Each reaction was carried out in a 50 μl volume containing 25 μl of 2× Phusion Flash High-Fidelity Master Mix (Finnzymes, Espoo, Finland), 200 nM of each primer and 80 ng of genomic DNA. The PCR profile was: 1 minute at 98°C for one cycle; 10 s at 98°C, 20 s at 65°C, 4 minutes at 72°C for 35 cycles; 10 minutes at 72°C for one cycle. PCR products were purified using QIAquick PCR Purification Kit (QIAGEN Venlo, Limburg, Netherlands), 150 ng of purified PCR product was biotinylated using the BioPrime DNA Labeling Kit (Invitrogen, Carlsbad, California, US) and unincorporated biotin-14-dCTP was removed by passing the samples through Sephadex G-50 columns (GE Healthcare Fairfield, Connecticut, US). Sample (150 ng) was added to the Affymetrix GeneChip Human Gene 1.0 ST array and hybridized at 45°C for 17 hours with a rotation of 60 rpm. Chips were washed and stained with SAPE (2× MES staining buffer, 20 mg/mL bovine serum albumin (BSA) and 1 mg/ml streptavidin-phycoerythrin), washed on an Affymetrix fluidics station and scanned.

Representation of the complete hORFeome collection was assessed using the same experimental conditions with some modifications. gDNA was extracted from at least 16 × 10^6^ cells (approximately 1,300 cells per hORF), 3 PCR reactions were performed using 2 μg of gDNA each (approximately 75 genomes per hORFs), two biotinylation reactions were performed using 500 ng of combined PCR products and 3.5 μg of sample was added to an Affymetrix GeneChip Human Gene 1.0 ST array.

Microarray raw data have been deposited in the ArrayExpress repository [15] under accession number E-MTAB-2493.

### Selection and individual validation of over-represented hORFs

The hORFs conferring resistance to the drug were identified in a step-wise procedure. Screen results were first visualized by plotting the log2 of the signal intensity for each hORF retrieved via PCR from cells cultured in the presence of drug (on the x-axis) and by plotting the log2 ratio of the signal intensity for each hORF of the cells cultured in the presence of drug divided by the signal intensity for each hORF of the cells grown in the presence of DMSO (on the y-axis). Genes with a log2(drug/DMSO) >3 and a log2(drug) >6 enrichment were then listed and ranked. Primary hits selected for validation were those previously enriched genes in common among the drug screen replicates (at least two independent experiments).

For validation, 10,000 HEK293-M2 cells were seeded in 300 μl of DMEM plus 10% iFBS plus penicillin/streptomycin in a 48-well plate pretreated with poly-L-lysine (Sigma, P4832). After 12 hours of incubation, gene expression was induced by the addition of doxycycline (2 μg/ml final concentration). After 18 hours of induction, drug was added and cell growth was assessed 3 days after drug addition by sulforhodamine B (SRB) assay as previously described [16].

### Immunostaining

The HEK293_M2 + RHOXF2 stable cell line (PB-TGcMV-Neo) were seeded in an eight-well chamber slide (BD Falcon, East Ruherford, New Jersey, US.) and cultivated overnight in the presence or absence of doxycyclin (2 μg/ml final). Cells were treated with 50 nM of mitomycin C for 2 days before fixation with 2% paraformaldehyde and permeabilization with 0.3% Triton X-100. Cells were blocked for 30 minutes with blocking/dilution buffer (10% goat serum, 0.5% NP-40, 0.5% saponin, 1× phosphate-buffered saline (PBS)) and incubated with the anti-phospho-histone H2A.X (serine 139, clone JBW301; Millipore, Billerica, Massachusetts, US) overnight at 4°C. Cells were then incubated with an Alexa 546 anti-mouse antibody for 1 hour at room temperature, incubated with DAPI (0.8 μg/ml; Sigma) for 10 minutes and mounted using ProLong Gold (Invitrogen). Images were captured using a 40× dry objective. Nuclei and γ-H2A.X foci were quantified using CellProfiler [17].

### RNA extraction and library preparation for gene expression profiling of the RHOXF2 overexpressing cell line

The HEK293_M2 + RHOXF2 stable cell line (PB-TGcMV-Neo) was seeded in 10-cm dishes, cultivated 24 hours in the presence or absence of doxycycline (2 μg/ml final) before being treated with 40 nM of mitomycin C or DMSO (control) during a 2-day period. Total RNA was extracted (RNeasy Mini Kit, QIAGEN), mRNA-focused libraries were generated from 1 μg of total RNA (Illumina TruSeq RNA Sample Preparation Kit V.2) and sequenced using an Illumina HiSeq2000 according to the Illumina protocol. Raw data have been deposited in the ArrayExpress repository under accession number E-MTAB-2497.

### RNA-sequencing data analysis

Single-end reads, 51 nucleotides in length, were generated on the Illumina HiSeq. Sequence data were aligned to the UCSC hg19 reference genome using TopHat (v2.0.0), provided with a RefSeq GTF file and instructed to align only across known junctions. Differential expression, as fragments per kilobase of exon per million fragments mapped (FPKM) differences, were generated by analysis with cuffdiff (v1.1.0) using the indicated comparisons. Based on their differential expression, potential activated and repressed isoforms were identified and related enriched biological processes were assessed using GOrilla [18].

### RHOXF2 quantitative RT-PCR

Total RNA from 20 different normal human tissues (each pool from 3 donors) were obtained from the FirstChoice Human Total RNA Survey Panel (Ambion, Austin, Texas, US). Total RNA (5 μg) from each tissue was used for oligo(dT)12-18-primed reverse transcription using the SuperScript II reverse transcriptase (Invitrogen). Quantitative PCR was performed using the LightCycler 480 SYBR Green I Master (Roche) and the LightCycler 480 System with the following primers:

QhRHOXF1_1F (5′-ACCGTGTTCTACTGCCTGAGTGTA) and QhRHOXF1_1R (5′-TTCATGCCGTTCTCGTGGTTCACA) on RHOXF1 exon 1; QhRHOXF1_2F (5′-TGGAGGAGCTGGAAAGTGTT) and QhRHOXF1_2.2R (5′-GGCCCTTTTATTCTTAAACC) spanning RHOXF1 exon 1 and exon 3; QhRHOXF2_1F (5′-CCGGACCAGTGTAGCCAGTA) and QhRHOXF2_1R (5′-TCTTTTTCTTCTCCGCCTTG) spanning RHOXF2 exon 1 and exon 2; QhRHOXF2_2F (5′-ATGGTGCTGTCGCTTACTGA) and QhRHOXF2_2R (5′-TCGAGGTCTCCTTCCCATAG) on RHOXF2 exon 2; QhRHOXF2_4F (5′-CAGCGGGATGAGAGATGATT) and QhRHOXF2_4R (5′-TTGGGGAATGTGAAAGAAGG) on RHOXF2 exon 3.

Housekeeping/reference genes were: QhCYCG_F (5′-CTTGTCAATGGCCAACAGAGG) and QhCYCG_R (5′-GCCCATCTAAATGAGGAGTTGGT) on CYCG (cyclophilin G); QhGUSB_F (5′-ACGCAGAAAATATGTGGTTGGA) and QhGUSB_R (5′-GCACTCTCGTCGGTGACTGTT) on GUSB (beta-glucuronidase); QhActbF1 (5′-GAAGTCCCTTGCCATCCTAAAAG) and QhActbR1 (5′-AGGACTGGGCCATTCTCCTTA) on ACTB (beta-actin); QhEef1a1F2 (5′-CTGAACCATCCAGGCCAAAT) and QhEef1a1R2 (5′-AGCCGTGTGGCAATCCA) on EEF1A1 (eukaryotic translation elongation factor 1 alpha 1); QhGAPDH_F1 (5′-CATGAGAAGTATGACAACAGCCT) and QhGAPDH_R1 (5′-AGTCCTTCCACGATACCAAAGT) on GAPDH (glyceraldehyde-3-phosphate dehydrogenase).

### RHOXF2 immunohistochemistry

Formalin-fixed, paraffin-embedded sections of human normal testis were deparaffinized for 12 hours at 58°C and rehydrated. Heat-induced epitope retrieval was in sodium citrate buffer pH 6.0 using a pressure cooker [19]. All antibodies were diluted in Tris-buffered Saline (TBS) with 1% BSA. Sections were blocked in 10% goat serum (Gibco) for 2 hours at room temperature. The anti-RHOXF2 primary antibody (Sigma, HPA003314) was used at a 1:150 dilution and incubated overnight at 4°C in a humid chamber. Endogenous peroxidase activity was suppressed by incubating the section in 0.3% H_2_O_2_ for 15 minutes. Horse radish peroxidase (HRP)-conjugated secondary antibody (anti-rabbit IgG-HRP; GE Healthcare) was used at a 1:250 dilution and incubated for 1 hour at room temperature. 3,3′-Diaminobenzidine (Abcam) was used as chromogen. Sections were counterstained with Mayer’s hematoxylin (Sigma) before dehydration and mounting.

### Western blotting

For the detection of endogenous RHOXF2, total cell lysates were prepared from 200,000 cells. Briefly, samples were lysed in 2× SB loading buffer (60 mM Tris-HCl pH6.8, 3% SDS, 10% glycerol, 0.05% bromophenol blue, 10% beta-mercaptoethanol) and subjected to SDS-PAGE (12%). The RHOXF2-specific mouse polyclonal antibody (Abcam, ab67811) and the tubulin specific rat antibody (Abcam, ab6160) were used at a dilution of 1:800 and 1:40,000 respectively. Immunological complexes were visualized by enhanced chemiluminescence using HRP-conjugated anti mouse or anti rat IgG.

### Drug dose-response curves

K562 cells (100,000) were cultured in 600 μl of media in a 48-well plate for 24 hours before the addition of diluted drug. Two days after drug treatment, the percentage of drug inhibition was assessed by counting K562 using a Coulter particle count and cell analyser and compared to cells grown in the presence of DMSO only.

For the adherent HPAC, SW1353, and NCI-H1299 cell lines, 5,000 cells were seeded in 300 μl of media in 96-well plate and incubated for 24 hours prior to drug addition. Following two days of drug treatment, the percentage of drug inhibition was estimated by SRB assay [16] and compared to cells grown in DMSO as control.

## Results

### Development of the assay: DHFR and methotrexate

**Figure 1:**
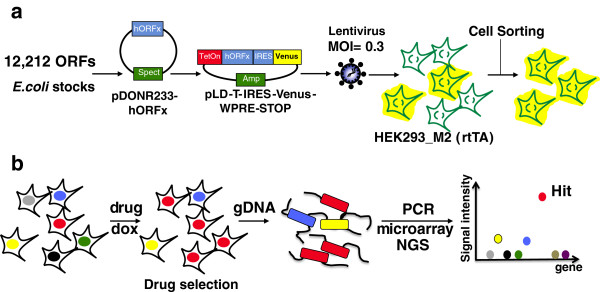
**A gain-of-function cell-based assay to characterize the mode of action of small chemical compounds. (a)** A collection of 12,212 hORFs was cloned *en masse* into the lentiviral expression vector pLD-IRES-Venus-WPRE-STOP. The resulting constructs were used to produce lentivirus and infect the rtTA-expressing cell line HEK293_M2 at a multiplicity of infection (MOI) of 0.3. Sorting of the Venus-positive cells resulted in the isolation of human cells with the functional integrated lentiviral constructs. **(b)** After seeding, doxycycline (dox) was added to the cells in order to induce the expression of the hORFs. After selection in the presence of a lethal dose of drug, surviving cells were harvested, gDNA extracted and the nature of the hORFs providing resistance to the chemical identified after hybridization on microarray. NGS, next-generation sequencing.

We developed an original phenotypic assay in which the mammalian cell line HEK293_M2 was used to identify hORFs capable of rescuing small molecule toxicity when overexpressed (Figure 1; Additional file 1). As a proof of concept, we rescued methotrexate toxicity by overexpression of its target, the gene encoding the enzyme dihydrofolate reductase (DHFR).

**Figure 2:**
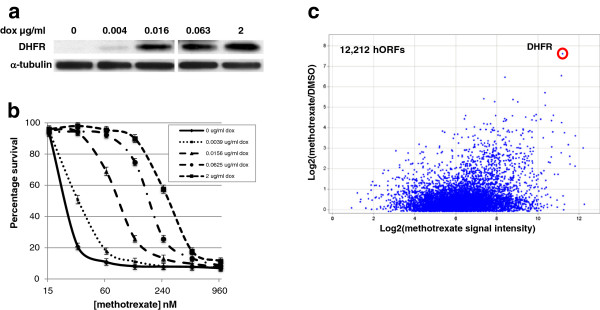
**DHFR was identified as the top candidate during a large-scale screen against methotrexate. (a)** Immunoblotting showing the conditional expression of DHFR in HEK293_M2 cells. Addition of 4 ng/ml, 16 ng/ml, 63 ng/ml and 2,000 ng/ml of doxycycline (dox) induced DHFR protein expression in the stable cell line HEK293_M2. Alpha tubulin was used as a loading control. For clarity, protein expression level at 0.0032 ng/ml of doxycycline has been omitted. **(b)** Single DHFR overexpression rescued methotrexate toxicity in HEK293_M2 cells. The gene coding for DHFR was cloned into the lentiviral vector and used to create stable HEK293_M2 cells able to conditionally express DHFR. Dose-response curves of the cells grown in the presence of an increasing concentration of doxycycline were calculated after 2 days of exposure to 15, 30, 60, 125, 250, 500 and 1,000 nM of methotrexate and compared with those for cells cultured in the presence of DMSO as control. Cell survival was quantified using a SRB assay. Errors bars represent the standard deviation for triplicate assays. **(c)** Identification of genes conferring resistance to methotrexate when overexpressed. HEK293_M2 cells harboring the 12,212 hORF collection were grown in the presence of a lethal dose of methotrexate. The nature of the hORFs conferring resistance to the drug was identified by plotting the log2 of the signal intensity for each hORF in the cells cultured in the presence of methotrexate on the x-axis; and by plotting the log2 ratio of the signal intensity for each hORF of the cells cultured in the presence of the drug divided by the signal intensity for each hORF of the cells grown in the presence of DMSO on the y-axis.

We first cloned the human *DHFR* gene into the Gateway-compatible lentiviral vector pLD-T-IRES-Venus-WPRE-STOP where gene expression is under the control of the Tet-On promotor (Additional file 2). After transduction of the rtTA-expressing cell line HEK293_M2 by lentivirus, we detected a high level of protein in the presence of doxycycline by western blotting (Figure 2a; Additional file 3). No protein was detected in the absence of doxycycline, suggesting robust control of transgene expression. Cells were next exposed to increasing doses of methotrexate in the absence or presence of different doses of doxycycline. Increasing levels of DHFR overexpression in HEK293_M2 cells rescued methotrexate toxicity in a dose-dependent manner, demonstrating that the lentiviral construct was functional (Figure 2b; Additional file 3).

We implemented our approach on a larger scale by cloning, *en masse*, a pool of 376 random hORFs (including *DHFR*) into the lentiviral vector. Corresponding lentivirus were used to generate a population of HEK293_M2 cells with the stably integrated hORFs. Gene expression was induced as before by addition of doxycycline and cells were exposed to a lethal concentration of methotrexate. Ten days after treatment, surviving cells were harvested, genomic DNA was extracted, and hORFs were PCR amplified and hybridized on an Affymetrix GeneChip Human Gene 1.0 ST array to determine which hORFs conferred resistance to methotrexate. *DHFR* was found as the predominant gene in this pool of 376 hORFs whose overexpression provided resistance to methotrexate, validating our strategy at the scale of several hundred pooled clones (Additional file 4).

The same approach was next applied to the complete collection of 12,212 hORFs. Here, we divided the collection into 34 minipools of 376 hORFs each, transferred them *en masse* into the pLD-T-IRES-Venus-WPRE-STOP lentivirus vector and then pooled 34 minipools together to produce sufficient quantities of lentivirus for the infection of HEK293_M2 cells at a multiplicity of infection of 0.3 (Figure 1). Because induction of the Venus marker is dependent on hORF expression, addition of doxycycline for 24 hours generated sufficient fluorescence to sort those cells with stable lentivirus integration (Additional file 5). The presence and relative abundance of the virally integrated hORFs were assessed using next-generation sequencing. Based on the identification of at least 95% of the sequence with a minimum of 10× coverage, 87% of the mappable hORFs were considered as present in the HEK293_M2 cells. Further analysis of the sequencing results demonstrated a size-dependent relative abundance of the hORFs, with smaller clones being more represented in the population (Additional file 6). An aliquot of 500,000 HEK293_M2 cells (an average of 41 cells per hORF) was seeded in a T175 flask, and grown for 15 hours before the induction of hORF expression by addition of doxycycline. After 18 hours of doxycycline induction, cells formed micro-colonies, which were cultured in the presence of methotrexate for 2 weeks. hORFs derived from the surviving cells were identified by PCR amplification and microarray hybridization. As we found for the minipool experiment, *DHFR* was the top gene conferring resistance to methotrexate when overexpressed (Figure 2c). These results encouraged us to test the same strategy for drugs with diverse modes of action.

### Screens with additional drugs

We tested six additional diverse drugs, including the antifolate aminopterin, the lipase inhibitor orlistat, the Hsp90 inhibitor 17-(dimethylaminoethylamino)-17-demethoxygeldanamycin (a geldanamycin analog) and three DNA damaging agents (bleomycin, cisplatin and mitomycin C) based on the diversity of their mode of action (Table 1).

**Table 1:** Characteristics of screened compounds

**Drug name**	**Screen concentration**	Category*	**Clinical use**	**Known target(s)**	**Reference**
Methotrexate	60 nM	Antimetabolites/ immunosuppressants	Antineoplastic	DHFR	[20]
			Antirheumatic		
			Dermatologic agent		
Aminopterin	20 nM	Antimetabolites (discontinued)	Dermatologic agent (phase I)	DHFR, FPGS(?)	[21]
			Antineoplastic agent (phase II)		
Orlistat	20 μM	Antiobesity preparations, excluding diet products	Anti-obesity agent	LPL	[22]
				PNLIP	[23]
				FASN	
Bleomycin	1 μM	Cytotoxic antibiotics and related substances	Antineoplastic agent	DNA	[24]
				LIG1	
				LIG3	
Cisplatin	2 μM	Other antineoplastic agents	Antineoplastic agent	DNA	[25]
Mitomycin C	80 nM	Cytotoxic antibiotics and related substances	Antineoplastic agent	DNA	[26]
17_DMAG	70 nM	Experimental Hsp90 inhibitor	Antineoplastic agent (phases I and II)	Hsp90	[27]
				HSP90AA1	
				HSP90AA2	
				HSP90B1	
				HSP90AB1	

Each drug was screened at least twice and the top hits (those hORFs most enriched in the final sample of cells; see Methods) were selected for validation. Of 123 candidates, we successfully cloned 120 hORFs into the modified Gateway-compatible *piggyBac* vector PB-TGcMV-Neo (Additional file 7) and generated 120 independent HEK293_M2 stable cell lines. Protection against the drug toxicity was assessed two days after culturing cells in the presence of four concentrations of each drug with or without doxycycline induction. Relative cell densities were compared with cells containing an empty vector control construct and were quantified by SRB assay. A total of 17 unique hits were confirmed to rescue their respective drug toxicity (Table 2). The number of validated hits varied considerably according to the compound, with bleomycin and 17-DMAG having low validation rates (7% and 3%, respectively) and, in contrast, cisplatin and mitomycin C having high validation levels (27% and 42%, respectively) (Table 2). Several scenarios, both technical and biological, may explain this modest confirmation rate. First, PCR amplification of the hORFs from gDNA and probe cross-hybridization on microarrays can be expected to generate a certain percentage of false positives, despite each screen having been performed in duplicate. Second, since this version of the hORFeome library is not fully sequence verified, we speculate that some genes contain mutations that impair proper expression. We attempted to minimize the impact of mutations by restriction verifying four independent colonies from the original pDONR233 bacterial stock, cloning positive clones as a pool for subsequent cloning into the PB-TGcMV-Neo vector and transfection into HEK293_M2 cells. Finally, the biological mechanisms by which these agents work will influence the number of hits obtained from each screen, and therefore affect the number of hits that can be confirmed. For this proof-of-principle study we chose to be conservative, selecting more hits for validation at the cost of a high false-positive rate.

**Table 2:** Validated hits

**Drug**	**Gene name**	**Accession number**	**Description**	**Number of validated/tested candidate genes**
Methotrexate	*DHFR*	BC000192	Dihydrofolate reductase	1/3
Aminopterin	*DHFR*	BC000192	Dihydrofolate reductase	1/9
Bleomycin	*IMMT*	BC002412	Inner membrane protein, mitochondrial (mitofilin)	2/30
	*STX3*	BC007405	Syntaxin 3	
Cisplatin	*MAP2K1IP1*	BC026245	Mitogen-activated protein kinase kinase 1 interacting protein 1	4/15
	*MMD*	BC026324	Monocyte to macrophage differentiation-associated	
	*PTPN2*	BC016727	Protein tyrosine phosphatase, non-receptor type 2	
	*RHOXF2*	BC021719	Rhox homeobox family, member 2	
Mitomycin C	*C20orf54*	BC009750	Chromosome 20 open reading frame 54	8/19
	*CCDC45*	BC009518	Coiled-coil domain containing 45	
	*ELF5*	BC029743	E74-like factor 5 (ets domain transcription factor)	
	*PTPN2*	BC008244	Protein tyrosine phosphatase, non-receptor type 2	
	*RHOXF2*	BC021719	Rhox homeobox family, member 2	
	*TMEM150*	BC050466	Transmembrane protein 150	
	*USPL1*	BC038103	Ubiquitin specific peptidase like 1	
	*ZFP64*	BC012759	Zinc finger protein 64 homolog (mouse)	
17-DMAG	*EIF4B*	BC073139	Eukaryotic translation initiation factor 4B	1/29
Orlistat	*MGLL*	BC006230	Monoglyceride lipase	2/18
	*PON3*	BC070374	Paraoxonase 3	

### Screens for the antifolates methotrexate and aminopterin

For the antifolate screens, the single confirmed ORF was *DHFR*. This result highlights the fact that when a single hORF confers a strong growth advantage, it can dominate the other clones in the population and potentially mask the detection of other potential protective genes. One way to address this and potentially improve the dynamic range for this drug class would be to repeat these screens with a pool of hORFs lacking *DHFR*.

Among the potential expected hits present in our collection was the enzyme gamma-glutamyl hydrolase (GGH), which catalyzes the removal of polyglutamates from methotrexate. Therefore, overexpression of GGH would be expected to decrease the intracellular presence of methotrexate-polyglutamate, thereby increasing the efflux of methotrexate out of the cell and decreasing cytotoxicity. However, GGH overexpression was demonstrated to be insufficient to produce resistance to the drug in a human fibrosarcoma (HT-1080) and a human breast carcinoma (MCF-7) cell line [28].

DHFRL1 (dihydrofolate reductase like-1) consistently appeared as a top hit for screens with both methotrexate and aminopterin but failed to confer resistance when individually expressed in HEK293_M2 cells. Based on the strong similarity between DHFR and DHFRL1 (a total of 14 non-synonymous changes), we hypothesized that the observed unspecific signal was probably due to cross-hybridization. Interestingly, a similar problem occurred when the same strategy using the same hORFeome library was tested in the model organism *S. cerevisiae*. Both DHFR and DHFRL1 were enriched in the screens against methotrexate (and their expression confirmed at the protein level) but only DHFR conferred methotrexate resistance (data not shown).

### Orlistat screens

Orlistat is a lipase inhibitor that reportedly targets LPL (lipoprotein lipase), PNLIP (pancreatic lipase) and FASN (fatty acid synthase). Although LPL is absent from our collection, PNLIP and FASN are present. However, genes for the latter two did not confer overexpression resistance.

Our screen identified the gene coding for monoglyceride lipase (MGLL) as a resistance hit. MGLL hydrolyzes intracellular triglyceride stores in adipocytes and other cells to fatty acids and glycerol. The enzyme might also be involved in the hydrolysis of monoglycerides [29]. *In vitro* experiments suggest that orlistat can inhibit the MGLL-like activity in rat-cerebellar membranes and rat cerebellar homogenate [30], rendering the human MGLL a potential direct target for orlistat.

Another validated hit, *PON3*, is also involved in lipid metabolism. The gene encodes paraoxonase 3, which is secreted into the bloodstream and associates with high-density lipoprotein. The protein can also rapidly hydrolyze lactones and inhibit the oxidation of low-density lipoprotein.

### Bleomycin screens

Although the exact mechanism of action of bleomycin is unknown, available evidence indicates that its main mode of action is via the inhibition of DNA synthesis, with additional evidence for its inhibition of RNA and, to a lesser extent, protein synthesis [31].

We show that overexpression of IMMT (inner membrane protein, mitochondrial) partially rescues bleomycin toxicity. Bleomycin treatment induces damage of both nuclear and mitochondrial DNA [32]. A recent study demonstrated that the mitochondrial localization of PARP-1 requires interaction with IMMT and that it is involved in the maintenance of mitochondrial DNA integrity [33]. The authors also suggest that IMMT overexpression results in an increase of PARP-1 in the extracellular compartment. We therefore speculate that IMMT overexpression recruits sufficient PARP-1 into the mitochondrion to maintain mitochondrial DNA integrity and promote cell survival.

### Cisplatin and mitomycin C screens

RHOXF2 and PTPN2 were found to suppress the toxicity of cisplatin and mitomycin C. Together with the antifolates, these DNA damaging agents were the only screens yielding identical hits. Also, as for methotrexate and aminopterin, both these alkylating agents have the same mechanism of action.

Another validated hit for mitomycin C, ZFP64 (zinc finger protein 64 homolog), is noteworthy because it has been shown to be potentially phosphorylated by ATM and ATR, the major signal transducing kinases of the DNA damage response in response to ionizing radiation [34]. This observation is consistent with a potential role for ZFP64 in the repair of DNA damage generated by both alkylating agents and ionizing radiation.

### RHOXF2 overexpression confers resistance to several DNA damaging agents

**Figure 3:**
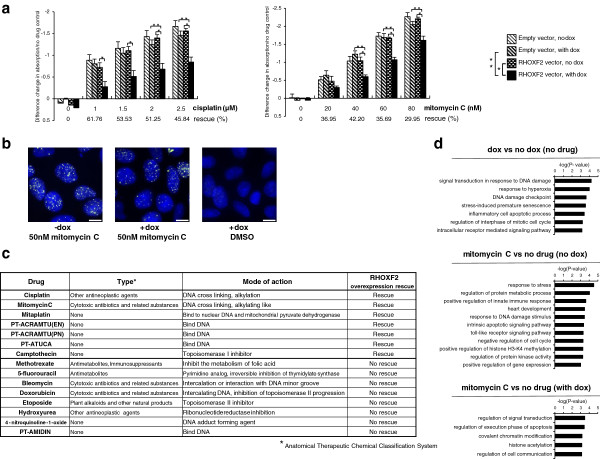
**RHOXF2 overexpression in HEK293_M2 cells conferred resistance to a wide variety of DNA damaging agents. (a)** In HEK293_M2 cells, RHOXF2 overexpression rescued cisplatin and mitomycin C toxicity. Stable RHOXF2-expressing cells were cultured in the presence of an increasing concentration of cisplatin or mitomycin C and their growth was compared to stable cells with the empty vector PB-TGcMV-Neo. The effect of RHOXF2 expression on cell viability was measured two days after drug exposure and compared to cells cultured in the absence of drug as a 100% viability control. *P* < 0.05, TukeyHSD test: *significant difference between RHOXF2 vector with and without doxycycline (dox); **significant difference between RHOXF2 vector with doxycycline and empty vector with doxycycline. Error bars represent standard error of the mean (n = 4). **(b)** Reduction in the number of γ-H2A.X foci by RHOXF2 overexpression in HEK293_M2 cells. Cells expressing or not RHOXF2 were treated with 50 nM of mitomycin C (approximately IC_10_) for 2 days and stained for the presence of γ-H2A.X foci. Following RHOXF2 overexpression, the number of γ-H2A.X foci per nucleus dropped from 20.71 ± 1.49 to 15.73 ± 0.92 (24% rescue). Nuclear DNA was stained with DAPI. Quantification of γ-H2A.X foci in >250 cells from triplicate experiments. Scale bar: 10 μm. **(c)** In HEK293_M2 cells, RHOXF2 overexpression conferred resistance to various DNA damaging agents. RHOXF2 drug suppression capacity was tested using DNA damaging agents with various modes of action as previously described for cisplatin and mitomycin C. **(d)** Gene Ontology analysis of upregulated genes in HEK293_M2 cells. Cells expressing or not RHOXF2 were treated with 40 nM of mitomycin C for 2 days. Based on their *P*-values, the most enriched biological processes are shown.

Among the top candidates from the screens and confirmations, we focused on RHOXF2, a relatively uncharacterized member of the homeobox gene family, because this gene, when overexpressed, provided resistance to both cisplatin and mitomycin C. RHOXF2 clearly reduced drug-induced toxicities when overexpressed in the HEK293_M2 cell line treated with cisplatin (1 to 2.5 μM) or with mitomycin C (20 to 80 nM). Rescue efficiency ranged from 45 to 60% and 30 to 42%, respectively (Figure 3a). This observation was confirmed using an independent cell biological assay in which overexpression of this transcription factor significantly reduced the formation of γ-H2A.X foci in cells treated with 50 nM of mitomycin C for 2 days (Figure 3b).

We next asked if RHOXF2′s protective effect was restricted to cisplatin and mitomycin C or if this gene, when overexpressed, could also suppress the toxicity of DNA damaging agents with different mechanisms of action. Accordingly, we assessed the cytotoxicity of antimetabolites (methotrexate, 5-fluorouracil), a radiomimetic (bleomycin), replication inhibitors (camptothecin, doxorubicin, etoposide), mitaplatin, 4-nitroquinoline-1-oxide and several experimental platinum anticancer agents (PT-ACRAMTU(EN), PT-ACRAMTU(PN), PT-ATUCA, PT-AMIDIN) on HEK293-M2 cells expressing or not the transcription factor (Figure 3c; Additional file 8). With the exception of the topoisomerase I inhibitor camptothecin, all drugs whose toxicity was suppressed by RHOXF2 overexpression directly bind to DNA to exert their effects.

These observations motivated us to ask (i) if and how RHOXF2, an uncharacterized transcription factor, modulates gene expression in HEK293_M2 cells and (ii) if the nature of the regulated genes can illuminate the protective response to DNA damaging agents. We cultured HEK293_M2 cells with or without overexpression of RHOXF2 in the presence of 40 nM of mitomycin C or DMSO (control) and profiled their transcriptional response by RNA-seq. The sequencing data confirmed the RHOXF2 transcript was absent in the uninduced HEK293_M2 samples and verified its strong induction in the presence of doxycycline regardless of the drug treatment (Additional file 9). Neither RHOXF1 nor RHOXF2B expression was affected by RHOXF2 overexpression (Additional file 9). Interestingly, and despite their limited number, Gene Ontology analysis of the genes whose expression was induced during RHOXF2 overexpression showed that the most significantly enriched pathways are related to DNA damage processes (*P* < 7 × 10^-5^ and *P* < 3 × 10^-4^), stress response (*P* < 3 × 10^-4^), apoptosis (*P* < 4 × 10^-4^) and cell cycle (*P* < 7 × 10^-4^) (Figure 3d; Additional file 9). As anticipated, in the absence of RHOXF2 and the presence of mitomycin C, we found a strong enrichment for genes related to the response to stress (*P* < 3 × 10^-5^) and regulation of protein metabolic process (*P* < 9.5 × 10^-5^). Finally, in the presence of RHOXF2, genes involved in the regulation of signal transduction (*P* < 4 × 10^-4^) were induced during drug treatment. Therefore, we suggest that when treated with DNA damaging agents, the protective effect conferred by overexpression of RHOXF2 is due, in part, to its activation of several key genes involved in the response to DNA damage.

Finally, we asked if the RHOXF2-dependent protective effect observed in HEK293_M2 cells was restricted to one cell type. We then tested if RHOXF2 overexpression could confer resistance to the rtTA-expressing human breast adenocarcinoma cell line MCF7_M2 and the adenocarcinomic human alveolar basal epithelial cell line A549_M2. As quantified by both a two-fold shift in IC_50_ (44.91 μM to 88.17 μM) and by the 21% decrease in toxicity at 3 μM, RHOXF2 overexpression protected MCF7_M2 cells against cisplatin-mediated toxicity (Additional file 10a). In the absence of RHOXF2, addition of doxycycline resulted in a modest increase in IC_50_ (41.06 μM to 58.59 μM; empty vector). The effect of the vector alone, while detectable, is less than that observed in the presence of RHOXF2. In contrast, although RHOXF2 overexpression in A549_M2 cells provided some apparent resistance (shift in IC_50_ from 448 nM to 1,099 nM), the measured effect was, in fact, largely due to doxycycline (shift in IC_50_ from 718 nM to 1,454 nM with empty vector) (Additional file 10b). In conclusion, our experiments suggest that the protective effect of RHOXF2 is not confined to a single genetic background and expression of the transcription factor is sufficient to protect both HEK293 and MCF7 cells against the DNA damaging agent cisplatin. As with all large-scale screens, the biological significance of this observation should be further confirmed, for example, by interrogating a larger panel of cell lines.

Based on its viability and transcriptional phenotypes in HEK293_M2 cells, we further investigated the potential role for RHOXF2 in neoplastic cells.

### In normal tissue, RHOXF2 expression is confined to testis but RHOXF2 is expressed in several cancer cell lines

**Figure 4:**
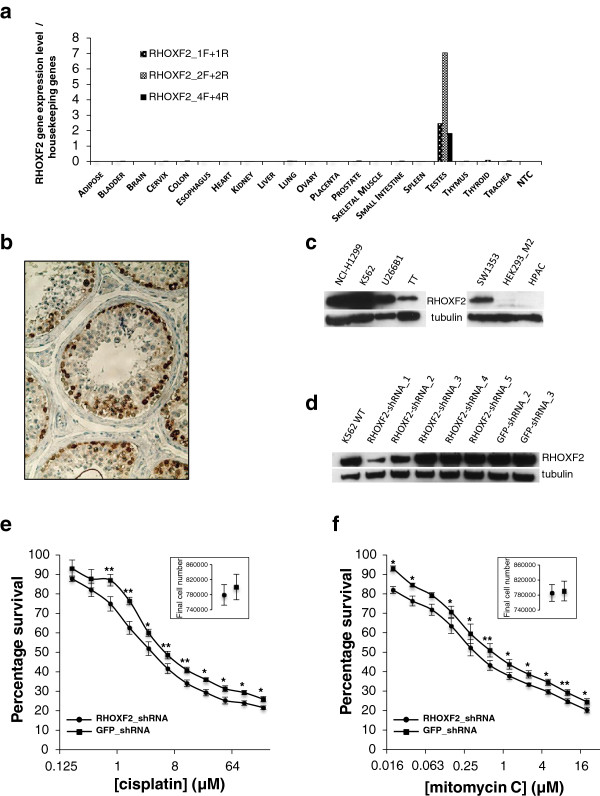
**RHOXF2 is expressed in various cancer cell lines and modulates K562 resistance to DNA damaging agents. (a)** In normal human tissues, RHOXF2 was exclusively detected in testis. Total RNA from 20 distinct tissues (mix of 3 different donors) was reverse transcribed into first strand cDNA and used as template for quantitative PCR. Three distinct pairs of primers covering exons 1, 2 and 4 were used. RHOXF2 expression level was calculated relative to the signal obtained for the amplification of housekeeping genes (CYCG, GUSB, ACTB, EEF1A1, GAPDH). **(b)** Representative example of immunohistochemical staining for RHOXF2 in normal testis. Section has been counterstained with Mayer’s hematoxylin. **(c)** RHOXF2 was detected in various tumor cell lines by western blotting. **(d)***RHOXF2* shRNA validation in the human chronic myelogenous leukemia cell line K562. WT, wild type. **(e,f)** Partial depletion of RHOXF2 increased K562 sensitivity to cisplatin and mitomycin C. Final numbers of viable cells were calculated in the absence of drug (inset). Student’s *t*-test; *P < 0.05; **P < 0.01; means ± standard error of the mean for n = 4 experiments.

In a small-scale study performed by northern blot analysis on normal tissues, expression of the transcription factor RHOXF2 was initially reported to be restricted to testis [35]. We performed a large-scale systematic survey interrogating a more representative panel of 20 different normal human tissues (3 donors per tissue type) by quantitative RT-PCR (Figure 4a). We first confirmed RHOXF2′s testis-specificity and then investigated which specific testis cell types expressed RHOXF2 protein by immunohistochemistry on normal testis samples. We detected a strong signal for RHOXF2 in spermatogonia and primary spermatocytes but not in spermatids (Figure 4b). Because RHOXF2 gene expression was detected in early stage spermatogenic cells and not in later stages, we hypothesized that RHOXF2 might be involved in the self-renewal or maintenance of the undifferentiated state of the testis germ cells.

Computational analysis predicts RHOXF2 to be a testicular cancer candidate gene [36] and, in fact, the transcript was detected in several types of human testicular cancers [37]. Furthermore, in the present study, we show that RHOXF2 overexpression conferred resistance to cisplatin (Figure 3a) and that RHOXF2 is found in several other cancer cell lines, the non-small cell lung carcinoma NCI-H1299, the blast phase chronic myelogenous leukemia K562, the multiple myeloma U266B1, the thyroid gland medullary carcinoma TT (CRL-1803) and the chondrosarcoma SW1353 (Figure 4c; Additional file 11).

### In the chronic myelogenous leukemia K562 cell line, RHOXF2 depletion modulates the response to antineoplastic agents

Our study showed that RHOXF2 overexpression confers resistance to cisplatin and mitomycin C in HEK293_M2 cells, in which the protein is not endogenously expressed (Figure 4c; Additional file 9). We first generalized this observation by positing that overexpression or ectopic expression of certain genes greatly reduces some detrimental drug effects on the growth of particular cancer cell lines. We then reasoned that if this is truly drug-gene specific, the opposite mechanism should experimentally verify it. More specifically, depletion of the endogenously expressed protein should confer sensitivity to the cell line during drug treatment. The most direct translation of this gene-drug specificity could then be exploited to understand drug resistance in cancer cell lines. Therefore, we asked if sensitivity to DNA damaging agents was observed when RHOXF2 is knocked down in cancer cells where the protein is normally expressed. We generated several stable cell lines to deplete RHOXF2 (NCI-H1299, K562, SW1353, and the human pancreatic HPAC as negative control) with five shRNAs that target the mRNA. Two shRNAs against GFP were used as a negative control. Two shRNAs showed a strong knock-down of the protein in all three RHOXF2-positive cell lines (Figure 4d; Additional file 12e,h).

K562 showed a significant increase in sensitivity for both cisplatin (IC_50_: 3.231 μM to 2.052 μM) and mitomycin C (IC_50_: 377.8 nM to 250.4 nM) when the transcription factor was depleted, demonstrating the potential role of RHOXF2 in response to DNA damaging agents and particularly in response to DNA crosslinking agents (Figure 4e,f). Independent K562 transduction resulted in identical sensitivity to the drugs, showing the phenotype is independent of the lentiviral integration site. We ruled out the possibility that the calculated drug sensitivity simply reflected a growth difference among cell lines by demonstrating that the RHOXF2_shRNA and GFP_shRNA control cell lines divided at the same rate (Figure 4e,f, insets). Finally, we assessed the robustness of our knock-downs in the presence of the drugs by confirming that the modulation of RHOXF2 protein level is maintained even when the K562 cells were exposed to cisplatin or mitomycin C at IC_70_ (Additional file 13a,b).

As expected, no change was measured for the RHOXF2-negative HPAC cells (Additional file 12a,b). Interestingly, no increase in sensitivity to cisplatin and mitomycin C was observed for NCI-H1299 and SW1353 knock-down cell lines (Additional file 12c,d,f,g).

The modest increase in sensitivity of K562 cells to DNA damaging agents can be explained by different factors. First, transcription factors are generally present in limited quantities in cells [38-40]. Here, although clearly depleted in the presence of the shRNAs, RHOXF2 is still present at a relatively high level in K562 cells (Figure 4d). Therefore, we speculate that despite the efficient knock-down, the limited shift in IC_50_ is in part due to the presence of enough protein to sustain a certain level of resistance during drug treatment. In order to test our first hypothesis, a complete knock-out of the transcription factor would be necessary (for example, using CRISPR technology). Secondly, a recent study in mouse embryonic stem cells reports that knock-down of a few transcription factors is associated with substantial transcriptome change [41]. These data not only indicate the robustness of the pluripotency gene network but also suggest that perhaps RHOXF2 knock-down hardly perturbs the transcription factor network in the K562 cellular context.

## Discussion

We have reduced to practice an elegant method to simultaneously evaluate thousands of genes for their capacity to provide a survival advantage in the presence of a toxic dose of drug using cells expressing the entire hORF collection and a microarray or sequencing-based readout.

The availability of hORF collections represents great progress compared to traditional approaches (random mutagenesis, cDNA libraries, and so on) because it permits the systematic interrogation of a gene's biological function in a targeted manner. Such gain-of-function approaches would be bolstered when combined with the reciprocal loss-of-function approach [42]. To date, several studies have already explored the availability of such collections. Most of the studies tested the hORFs function in an array (well-based) format [43-45] that simplifies the readout and analysis of the results. Here, we tested the function of thousands of ORFs simultaneously in a competitive manner. Our pool approach greatly reduced the tedious and expensive upstream steps of cloning, transduction and testing of the genes. The primary challenges to the pool approach are (i) potential loss of ORFs during cloning and infection (leading to representation variability), (ii) the potential masking effect of overrepresented clones in a population during selection (for example, DHFR in the case of methotrexate) and (iii) the necessity for dedicated analysis of the final results (hits prioritization). In addition to future enhancements of the human ORFeome collection, other promising gain-of-function approaches to assess drug mode of action are the development of functional variomics technology [46] and the exploration of human isoform space in human cell lines.

In developing the assay, we first determined the optimal period of time between the beginning of the gene expression and the addition of the chemical compound. To address that question, we quantified the number of Venus-positive cells 12 hours, 24 hours and 48 hours after induction of expression of the hORF collection. Because the human gene and the Venus marker are co-transcribed, quantifying the level of Venus expression by flow cytometry serves as a proxy for the level of human gene expression. Because the number of Venus-positive cells peaks at 24 hours after doxycycline addition (Additional file 5), we postulated that 18 hours post-induction would be sufficient time after which to add drug to test the human gene for any protective effects.

We then addressed the sensitivity of the technique by improving the ratio between the number of seeded cells, the growth area and the time of drug addition. Seeding 500,000 cells in a T175 flask 33 hours before drug addition provided enough time and density for isolated cells to form separate micro-colonies that should overexpress only one or a few genes. This 'clonogenic assay approach' was more sensitive to lower drug concentrations and provided a better selective environment for the surviving cells, presumably by minimizing signaling from a large population of dying cells that would arise at higher plating densities. Similarly, adding fresh medium and drug every 48 hours for the duration of the screen improved the selection process by washing away the dead and dying adherent cells that could have interfered with the subsequent screen readout.

During the course of the screen, the cell population followed a stereotypical pattern of growth, starting with a dramatic decrease in cell number and disappearance of the majority of cells, followed by a stabilization of the population and concluding with the development of medium to large size colonies originating from the initial surviving cells. Daily visual inspection of the cell population was crucial to determine when to stop the drug selection and harvest the cells, typically after 20 to 30 doubling times.

Several refinements may enhance this assay. First, improving the coverage and expression quality of the human genes using a more complete collection of fully sequenced ORFs as such collections become available [14]. Moreover, as illustrated in Additional file 6, relative abundance of the virally integrated hORFs is size-dependent. Therefore, to correct for that variation, preparation of the normalized amount of plasmid DNA for lentivirus production could be done as follows: i) reduce the pool size (less than 376 hORFs per pool as in our study); ii) hORFs should be grouped by gene length. One can also match the particular drug under interrogation to disease-appropriate cell types and environmental conditions. Whenever possible, experiments should be performed with an initial high number of cells to guarantee the full representation of the collection during drug target screening. Although DNA damaging resistance was observed in the RHOXF2-overexpressing HEK293_M2 cells, it would be informative to further study if that observation is dependent on RHOXF2 expression status. Finally, next-generation sequencing will facilitate the throughput of hit detection and reduce the number of false positives arising from microarray cross-hybridization.

Our study revealed several genes whose functions relate to the action of the drugs tested. RHOXF2 is of a particular interest because of (i) its strong rescue phenotype in HEK293_M2 cells, (ii) its capacity to suppress both cisplatin and mitomycin C toxicity, and (iii) the dearth of functional information on this transcription factor. Homeobox genes encode transcription factors that play a central role during embryogenesis. *RHOXF2* belongs to the Rhox family of genes, which are expressed not only during embryogenesis but also in adult reproductive tissue [47]. In normal tissue, RHOXF2 expression is likely restricted to testis but its function remains largely unknown [35,48]. Here, we demonstrated that (i) the presence of RHOXF2 was sufficient to lower both cisplatin and mitomycin C toxicity in HEK293_M2 cells, and (ii) the transcription factor might exert its effect via the activation of genes involved in response to DNA damage. Furthermore, RHOXF2 protein expression was detected in several cancer cell lines. In one of these lines (chronic myelogenous leukemia/K562) knocking down the protein increased the toxicity for both DNA damaging agents. Interestingly, although no growth disadvantage was observed in K562 cells partially depleted for RHOXF2 (Figure 4e,f), its depletion via shRNA increased the sensitivity of this leukemia cell line to several pharmacological compounds. We therefore speculate that although not a driver of tumorigenesis, RHOXF2 could confer on the neoplastic cells a selective advantage during drug treatment.

## Conclusions

We demonstrate the usefulness of our approach in order to understand drug mechanisms of action by combining genetic perturbation with drug treatment. Here we recapitulated examples of drug resistance and uncovered several unanticipated drug-target interactions by virtue of the suppression of chemical toxicity observed upon gene overexpression. We also provide a step-by-step protocol for performing such screens and for analyzing the data (Additional file 1). Moreover, our innovative overexpression approach could also be applied to identify genes whose up-regulation could be toxic or enhance cell proliferation in different cell types. For example, the assay could be useful to uncover tumor-specific activity in the context of genome engineering as well as for the discovery of cell-type-specific oncogenes.

## Abbreviations

BSA: bovine serum albumin; DHFR: dihydrofolate reductase; DMEM: Dulbecco's modified Eagle's medium; gDNA: genomic DNA; GGH: gamma-glutamyl hydrolase; hORF: human ORF; HRP: horse radish peroxidase; MGLL: monoglyceride lipase; ORF: open reading frame; PBS: phosphate-buffered saline; PCR: polymerase chain reaction; RNAi: RNA interference; shRNA: short hairpin RNA; SRB: sulforhodamine B.

## Competing interests

The authors declare that they have no competing interests.

## Authors’ contributions

AA, SK, GG and CN contributed to the conception and design of this project. AA and CN developed the methodology. AA and SK performed experiments. ABM constructed the pLD-T-IRES-Venus-WPRE-STOP vector. AIS performed quantitative PCR experiments. DT performed the Illumina RNA sequencing. AA and LEH analyzed and interpreted the data. AA, SK, LEH, AIS, JM, GG and CN wrote the manuscript. CN supervised the study. All authors read and approved the final manuscript.

## Supplementary Material

Additional file 1 Step-by-step protocol for a genome-wide mammalian overexpression drug screen. Click here for file

Additional file 2 Plasmid map of the lentiviral vector pLD-T-IRES-Venus-WPRE-stop. Click here for file

Additional file 3 **Original immunoblotting showing the conditional expression of DHFR in HEK293_M2 cells.** Addition of 4 ng/ml, 16 ng/ml, 32 ng/ml, 63 ng/ml and 2,000 ng/ml of doxycycline induced DHFR protein expression in the stable cell line HEK293_M2. Expression level was compared to alpha tubulin as a loading control. The asterisk marks a human protein that cross-reacts with the anti-DHFR antibody. Click here for file

Additional file 4 **DHFR was identified as the predominant gene in this pool of 376 hORFs whose overexpression provided resistance to methotrexate.** HEK293_M2 cells harboring a minipool collection of 376 hORFs (including *DHFR*) were grown in the presence of a lethal dose of methotrexate. The nature of the hORFs conferring resistance to the drug was identified by plotting the log2 of the signal intensity for each hORF in the cells cultured in the presence of methotrexate on the x-axis; and by plotting the log2 ratio of the signal intensity for each hORF of the cells cultured in presence of the drug divided by the signal intensity for each hORF of the cells grown in presence of DMSO on the y-axis. Click here for file

Additional file 5 **Detection of Venus-expressing HEK293_M2 cells after 12, 24 and 48 hours of induction. (a,b)** HEK293_M2 cells stably transduced with the hORFeome collection (b) or not (a) were cultured in the presence of doxycycline (2 μg/ml) for 12, 24 and 48 hours. After gene induction, cell number and intensity of the Venus fluorescence were measured by flow cytometry. The percentage of Venus positive cells was compared to the total number of living cells measured. Click here for file

Additional file 6 Human ORFeome relative abundance in HEK293_M2s at T0. Click here for file

Additional file 7 Plasmid map of the transposon-based vector PB-TGcMV-Neo. Click here for file

Additional file 8 **In HEK293_M2 cells, RHOXF2 overexpression conferred resistance to various DNA damaging agents.** Stable RHOXF2 cells was cultured in the presence of an increasing concentration of DNA damaging agents and their growth was compared to the stable cells with the empty vector PB-TGcMV-Neo. The effect of RHOXF2 expression on cell viability was measured two days after drug exposure and compared to cells cultured in the absence of drug as a 100% viability control. TukeyHSD test, *P* < 0.05; *significant difference between RHOXF2 vector with and without doxycycline; **significant difference between RHOXF2 vector with doxycycline and empty vector with doxycycline. Error bars represent standard error of the mean (n = 4). Click here for file

Additional file 9 RHOXF2 raw FPKM values and Gene Ontology enrichment. Click here for file

Additional file 10 **RHOXF2 overexpression rescued cisplatin toxicity in HEK293_M2 and MCF7_M2 but not A549_M2 cells.** Stable RHOXF2 cells were cultured in the presence of an increasing concentration of cisplatin and their growth was compared to the stable cells with the empty vector PB-TGcMV-Neo. The effect of RHOXF2 expression on cell viability was measured three days after drug exposure and compared to cell cultured in the absence of drug as a 100% viability control. Lines show the nonlinear fit of a variable-slope dose-response model for **(a)** MCF7_M2 cells, **(b)** A549_M2 cells and **(c)** HEK293_M2 cells as positive control; **(d)** resulting IC_50_s. Data analysis was performed using the 'drc' package in R. Experiments were done in triplicate. Click here for file

Additional file 11 RHOXF2 was detected in various tumor cell lines by western blotting. Click here for file

Additional file 12 **RHOXF2 knocked-down in HPAC, NCI-H1299 and SW1353 cells does not modulate cisplatin and mitomycin C sensitivities. (a-g)** Dose-response curves for HPAC (a,b), NCI-H1299 (c,d) and SW1353 (f,g) were established after 2 days of exposure to cisplatin (a,c,f) or mitomycin C (b,d,g). Percentage of growth inhibition was calculated by comparing the number of cells treated with drug to the number of cells cultured in media with DMSO as control. Error bars represent standard error of the mean (n = 4). RHOXF2 depletion in NCI-H1299 (e) and SW1353 cells (h). Total cell extracts for each cell line were used to detect the presence of RHOXF2 by western blotting. Expression level was compared to the alpha tubulin as loading control. Click here for file

Additional file 13 **RHOXF2 knocked down in K562 cells. (a,b)** Cell lines in which RHOXF2 and GFP were knocked down were grown in the presence of 35 μM cisplatin (a) or 7 μM of mitomycin C (b) for 2 days. Total cell extracts for each cell line were used to detect the presence of RHOXF2 by western blotting. Expression level was compared to alpha tubulin as loading control. **(c)** Total cell extracts from a RHOXF2-expressing cell line and a cell line with GFP knocked down but overexpressing RHOXF2 were used to detect the presence of RHOXF2 by western blotting. Click here for file
