# Supplementary material for: A genome scale overexpression screen to reveal drug activity in human cells
Source: Genome Med. 2014 Apr 29;6(4):32. doi: 10.1186/gm549 (PMC4062067; doi:10.1186/gm549)
Supplement: Additional file 1 — Step-by-step protocol for a genome-wide mammalian overexpression drug screen. [file gm549-S1.docx]

**Additional file 1**

Step-by-step protocol for a genome-wide mammalian overexpression drug screen

To provide flexibility in term of experimental design, the overexpression protocol is divided into three modules: 1) the human ORFeome collection, 2) cell culturing and 3) test compounds. This modular design allows one to adapt and combine these modules to best fit the biological question.

| 1. | Select human open reading frames (hORFs) |
| --- | --- |

In our study we used the version 3.1 of a collection of 12,212 human ORFs (representing 10,214 distinct genes) obtained from Open Biosystems (Thermo Scientific, Cat. No. OHS5175). Recently, the version 8.1 was released and contains sequenced verified clonal ORFs (http://horfdb.dfci.harvard.edu/hv7/)

| 2. | Select a cell line |
| --- | --- |

We chose HEK293_M2 due to its ease of culture and to the constitutive expression of rtTA, a reverse tetracyclin-controlled transactivator necessary for the inducible expression of the cloned human genes upon addition of doxycyclin.

| 3. | Select a set of drugs/compounds |
| --- | --- |

We used a set of compounds with diverse activity in order to test the performance of our system.

| 1. | Large scale cloning of the hORFeome collection in the lentiviral expression vector pLD-T-IRES-Venus-WPRE-STOP. |
| --- | --- |

The collection of 12,212 hORFs was divided in 34 minipools of 376 hORFs (i.e. four 96 well plates of the collection/minipool). For each minipool, the hORFs were cloned en masse from the pDONR223 into the lentiviral expression vector pLD-T-IRES-Venus-WPRE-STOP by Gateway LR reaction.

We first performed 34 Gateway LR reactions (one per minipool) to transfer the hORFs from the pDONR223 vector to the lentiviral vector:

• Mix 150 ng pLD-T-IRES-Venus-WPRE-STOP + 150 ng of pDONR223 + hORFs.

• Add 2μL of LR Clonase II enzyme mix (Invitrogen, Cat. No. 11791–020).

• Incubate the reaction at 25°C for 16 hours.

• Add 1μL of Proteinase K. Incubate at 37°C for 10 minutes.

To select for the successfully cloned hORFs, each reaction mixtures were transformed into DH5α electro-competent cells and transformed bacteria were selected on LB + Ampicillin plates:

• On ice, gently mix 4μL of the LR reaction to 40μL of DH5α (ElectroMAX-DH5α-E-Cells, Invitrogen, Cat No. 11319–019).

• Pipette the cell/DNA mix into a chilled 0.1 cm cuvette (GenePulserCuvette, BioRad, Cat. No. 165–2089).

• Insert cuvette into the BioRad E.coli Pulser apparatus and deliver pulse (voltage set to 2.0 kV).

• Immediately add 1 mL of SOC medium and incubate 90 minutes at 30°C with gentle shaking.

• Spread 1 mL of SOC + cells on LB + agar + Ampicillin (100μg/mL) in a 23×23cm plate (BD Falcon, Cat. No. 351040) using sterile glass beads. Dry plates in flow hood if needed.

• Collect all bacterial colonies by scraping plate and proceed to lentiviral vector (with cloned hORFs) extraction using one maxi-prep reaction for each minipool.

Note: We optimized cloning reactions such that for each minipool of 376 hORFs, ~50,000 E.coli transformants per plate (i.e. 130 transformants per hORF) were obtained.

| 2. | Lentiviral particles were produced to create frozen “screen-sized” stocks of infected cells. Lentiviral particles harboring the hORFeome collection were produced according to the modified protocols provided by The RNAi Consortium (TRC) (http://www.broadinstitute.org/rnai/public/resources/protocols) as described below:   \| • \| For each of the 34 hORF minipools, normalize the plasmid DNA concentrations to 200 ng/μL and then pool an aliquot of each minipool. \| \| --- \| --- \| \| • \| Seed 10 mL of 293 T packaging cells at 350,000 cells/mL (total 3×10^6^ cells) in low-antibiotic growth media (DMEM + 10%FBS + 0.1X Pen/Strep) in 10 cm tissue culture dishes, in a total of 8 dishes. \| \| • \| Incubate at 37°C, 5% CO_2_ until cells reach ~70% confluence (~24 hours). \| \| • \| Transfect packaging cells with 5.4μg of packaging plasmid (psPAX2), 600 ng of envelope plasmid (VSV-G/pMD2.G), 6μg of pool plasmid mix (pLD-T-IRES-Venus-WPRE-STOP) using 18μL of FuGene 6 transfection reagent (Roche, Cat. No. 11-814-443-001). \| \| • \| Incubate transfected cells for 18 hours (37°C, 5% CO_2_) \| \| • \| Change media to remove transfection reagent and replace with 10 mL high-BSA growth media for viral harvests (500 mL DMEM + 10%FBS + Pen/Strep with 5.5 g BSA). \| \| • \| Incubate cells for 48 hours (37°C, 5% CO_2_) \| \| • \| Harvest media containing lentivirus, transfer media to a polypropylene storage tube. \| \| • \| Centrifuge the media containing virus at 1250 rpm for 5 minutes to pellet any packaging cells that were collected during harvesting. \| \| • \| Transfer the supernatant to a sterile polypropylene storage tube. \| \| • \| Aliquot virus (to reduce the number of freeze/thaw cycles) and store at −80°C. \| |
| --- | --- | --- | --- | --- | --- | --- | --- | --- | --- | --- | --- | --- | --- | --- | --- | --- | --- | --- | --- | --- | --- | --- | --- |

HEK293_M2 cells are infected with the lentiviral hORFeome collection at a multiplicity of infection of 0.3 - 0.4 (this low MOI minimizes the number of multiple hORFs integrations) as follows:

• Add 5×10^6^ cells to 80 mL of DMEM + FBS + Pen/Strep +8μg/mL of polybrene, aliquot 4 mL of the culture into four 6 well plates and immediately add the virus.

• 24 hours post-infection, replace medium with virus with fresh DMEM + FBS + Pen/Strep to help cells to recover and start dividing.

• Leave HEK293_M2 cells amplify for an additional 24 hours and add doxycycline (2 μg/mL) to induce the expression of the Venus fluorescent protein (for 24 additional hours). This should yield approximately 13×10^6^ cells.

• Harvest 9×10^6^ cells and prepare them for cell sorting as follows: after centrifugation, wash cells twice with filter sterilized pre-chilled 1xHank’s Balance Salt Solution (HBSS, Gibco, Cat. No. 14025–092) + 1%BSA + 0.5% EDTA, transfer on ice, gently resuspend in cold 1xHBSS + 1%BSA + 0.5% EDTA to a density of 1.8 - 2×10^6^ cells/mL and filter through a 70μm mesh (BD Falcon, Cat. No. 352235) prior to cell sorting.

• Sort HEK293_M2 cells using a BD FACS Aria Cell Counter (or equivalent). Isolate at least 2.2×10^6^ Venus positive cells (final yield is ~ 180 cells per hORFs).

• Amplify transduced HEK293_M2 cells, split into aliquots of 6.3×10^6^ cells each (~500 cells per hORFs) and freeze for future screens. Storage medium: 50% FBS + 40% complete medium + 10% DMSO.

The representation of each clone within the Human ORF collection in HEK293_M2 cells was assessed as follows:

• Because the DNA content of the typical diploid human cell is (6x×10^9^bp/human genome) × (660 g/mol/bp) / (6.02×10^23^mol) = 6.58×10^−12^ g/genome or ~6.5 picograms for 12,212 hORFs, 1 fold coverage requires 12,212 cells i.e. 12,212 × 6.58×10^−12^ g = 8×10^−8^ g = 0.08 ug of gDNA.

• Extract gDNA from a minimum of 16x10^6^ cells (~1,300 cells per genes). Lyse cells using SNET buffer (10 mM Tris pH 8.0, 0.1 M EDTA, 0.5% SDS, 0.1 mg/mL Proteinase K, 25μg/mL RNAse A). Isolate genomic DNA using 1 volume of Phenol/Chloroform/Isoamyl Alcohol (25:24:1) pH 8.0, precipitate DNA by adding 2 volumes of 95% Ethanol (−20°C), wash with ice cold 70% Ethanol and resuspend in Tris–HCl pH 8.0.

• Amplify the collection by combining the product of three PCR reactions (~75 fold representation) using the forward and reverse primers specific for the inserted human ORFs, 5′-CGGTACCCGGGGATCCTCTAGTCAGCTGAC and 5′-CCATTTGTCTCGAGGTCGAGAATTCTAGCTAGAATC respectively. Each reaction was carried out in a 50μL volume containing 25μL of 2X Phusion Flash High-Fidelity Master Mix (Finnzymes), 200 nM of each primer and 2μg of genomic DNA. The PCR profile was 1 min at 98°C for one cycle; 10 sec at 98°C, 20 sec at 65°C, 4 min at 72°C for thirty five cycles; 10 min at 72°C for one cycle.

• Perform 2 biotinylation reactions of 500 ng purified PCR product each (BioPrime DNA Labeling Kit, Invitrogen, Cat. No. 18094–011).

• Remove unincorporated biotin-14-dCTP by passing the samples through Sephadex G-50 columns (GE Healthcare, Cat. No. 17-0573-01).

• Hybridize 3.5μg of resulting probes to Affymetrix GeneChip Human Gene 1.0 ST array (Cat. No. 901087) as described in Ketala et al. (2011). A comprehensive platform for highly multiplexed mammalian functional genetic screens. Ketela T, Heisler LE, Brown KR, Ammar R, Kasimer D, Surendra A, Ericson E, Blakely K, Karamboulas D, Smith AM, Durbic T, Arnoldo A, Cheung-Ong K, Koh JL, Gopal S, Cowley GS, Yang X, Grenier JK, Giaever G, Root DE, Moffat J, Nislow C. BMC Genomics. 2011 May 6;12:213.

| 3. | Screens are performed in duplicate (at least) and all cells are collected at the end of the drug selection. We grew HEK293_M2s in T175 flasks with one or several flask(s) containing drug(s) and a flask without (as reference). |
| --- | --- |

• Seed an aliquot of 6.3x10^6^ HEK293_M2 cells in 40 mL of DMEM + 10%FBS + Pen/Strep in a T175 flask, and leave the cells to recover for 24 to 36 hours before starting the screens.

• Seed aliquots of 500.000 HEK293_M2 cells in T175 flasks, grow cells for 15 hours and induce the hORFs expression by adding doxycycline (2μg/mL final concentration).

• After 18 hours of induction, cells should form micro-colonies expressing one or a few hORFs only. Such a cell density (i) renders the assay more sensitive to low drug concentrations and (ii) improves the positive selection by eliminating the sensitive cells for the flask surface without affecting “healthy” neighboring colonies.

• Add drug or DMSO (as reference) to the medium and culture cells for 2 to 3 weeks until distinct colonies are detectable. During drug treatment, it is crucial to add fresh medium with drug every 48 hours to maintain the selection process and wash away the dead and dying adherent cells that could interfere with the subsequent screen readout.

• During the course of the screen, the cell population follows a stereotypical pattern of growth; starting with a dramatic decrease in cell number and disappearance of the majority of cells, following by a stabilization of the population and concluding with the development of medium to large size colonies originating from the initial surviving cells. Daily visual inspection of the cell population was essential to determine when to stop the drug selection and harvest the cells, typically after 20 to 30 doubling times.

• Collect all cells, wash once with PBS and store pellets at −80°C.

| 4. | Assessment of human ORFs conferring resistance to drug treatment. |
| --- | --- |

During the selection experiments, only a minority of hORFs protected HEK293_M2s against the cytotoxic effect of the drug. Compared to the initial cell population, HEK293_M2 harboring the most resistant genes should be over-represented. The set of resistant genes from this population, PCR amplification, biotinylation and probe hybridization was performed using less DNA compared to the amount used in the representation experiments:

• Once the screen is completed, extract gDNA from all resulting cells (cell number will vary from screen to screen). Lyse cells using SNET buffer (10 mM Tris pH 8.0, 0.1 M EDTA, 0.5% SDS, 0.1 mg/mL Proteinase K, 25μg/mL RNAse A). Isolate genomic DNA using 1 volume of Phenol/Chloroform/Isoamyl Alcohol (25:24:1) pH 8.0, precipitate DNA by adding 2 volumes of 95% Ethanol (−20°C), wash with ice cold 70% Ethanol and resuspend in Tris–HCl pH 8.0.

• PCR amplify the “resistant” genes from 80 ng of genomic DNA. Use the forward and reverse primers specific for the inserted human ORFs, 5′-CGGTACCCGGGGATCCTCTAGTCAGCTGAC and 5′-CCATTTGTCTCGAGGTCGAGAATTCTAGCTAGAATC respectively. PCR reaction was carried out in a 50μL volume containing 25μL of 2X Phusion Flash High-Fidelity Master Mix (Finnzymes), 200 nM of each primer and 80 ng of genomic DNA. The PCR profile was 1 min at 98°C for one cycle; 10 sec at 98°C, 20 sec at 65°C, 4 min at 72°C for thirty five cycles; 10 min at 72°C for one cycle.

• Perform a single biotinylation reaction of 150 ng of purified PCR product (BioPrime DNA Labeling Kit, Invitrogen, Cat. No. 18094–011).

• Remove unincorporated biotin-14-dCTP by passing the samples through Sephadex G-50 columns (GE Healthcare, Cat. No. 17-0573-01).

• Hybridize 150 ng of resulting probes to Affymetrix GeneChip Human Gene 1.0 ST array (Cat. No. 901087).

Primary hits are selected based on signal intensities of individual hORFs from cells cultured in the presence of drug or DMSO:

• Visualize screen results by plotting the log2 of the signal intensity for each hORF retrieved via PCR from cells cultured in the presence of drug (on the x-axis) and by plotting the log2 ratio of the signal intensity for each hORFs of the cells cultured in presence of the drug divided by the signal intensity for each hORFs of the cells grown in presence of DMSO on the y-axis.

• Genes with a log2(drug/DMSO) > 3 and a log2(drug) > 6 enrichment were then listed and ranked.

• Primary hits selected for validation were those previously enriched genes in common among the drug screen replicates (at least two independent experiments).

Note: log transformed signal intensities of each hORF (on the x-axis) minimized the number of false positives for which high log2 ratios would mainly originate from initial low signal intensities (typically below 1) in the absence of drug.

5. Primary hit validation

Candidate genes are then individually validated in HEK293_M2s using identical hORFs cloned into a modified piggyBac vector:

• Retrieve four restriction verified colonies from the original pDONR233 bacterial stock, pool the DNA extractions and clone into the piggyBac vector PB-TGcMV-Neo by LR reaction (LR Clonase II enzyme mix, Invitrogen, Cat. No. 11791–020).

• Generate stable HEK293_M2 cell lines harboring the individual candidate hORFs and an additional stable cell line with the empty vector.

• Seed HEK293_M2 cells in 48-well plates at 10,000 cells/well in 300μL of media. Note: wells are preteated with poly-L-Lysine (Sigma, Cat. No. P4832).

• After 12 hours incubation, induce the hORF expression by addition of doxycycline (2μg/mL).

• Drugs were serially diluted first in DMSO then in DMEM, with the final DMSO concentration not exceeding 0.5%.

• After 18 hours incubation, add drug as follows in a 48 well plate: the top two rows contain vehicle control (considered the 100% growth control) and the 4 remaining rows with a serial dilution of the drug.

• Three days later, assess relative cell growth by sulforhodamine B (SRB) viability assay, described below.

• Fix HEK293_M2s by addition of trichloroacetic acid (TCA) from a 10% stock in water to 3.3% w/v final concentration, incubate at 4°C for 30 minutes, then wash gently 5 times with running tap water.

• Fixed precipitates were air dried and 100 μL of 0.4% w/v of SRB dissolved in 1% acetic acid was added to each well and incubate at room temperature for 30 min on a rocking platform.

• Remove unbound SRB with 4 washes of 1% acetic acid and then air dry the plates. Solubilize the bound dye by adding 300μL of 10 mM Tris base (pH 10.5). After 10 minutes determine the solubilized dye concentration with a absorbance reader set at a wavelength of 510 nm (e.g. BioTek Synergy 2). Potential drug resistance was tested for the HEK293_M2s harboring the candidate hORFs and the PB-TGcMV-Neo empty vector. The percentage of growth inhibition was calculated by dividing the absorbance obtained from the cells treated with drug by the absorbance of the cells cultured in presence of vehicle alone (100% growth control). The “empty vector” control evaluates any possible influence of the vector backbone on the cell viability during drug treatment. Background signal subtraction was based on wells incubated in the absence of cells.
