# Supplementary material for: A genome scale overexpression screen to reveal drug activity in human cells
Source: Genome Med. 2014 Apr 29;6(4):32. doi: 10.1186/gm549 (PMC4062067; doi:10.1186/gm549)
Supplement: Additional file 2 — Plasmid map of the lentiviral vector pLD-T-IRES-Venus-WPRE-stop. [file gm549-S2.pptx]

## Slide 1
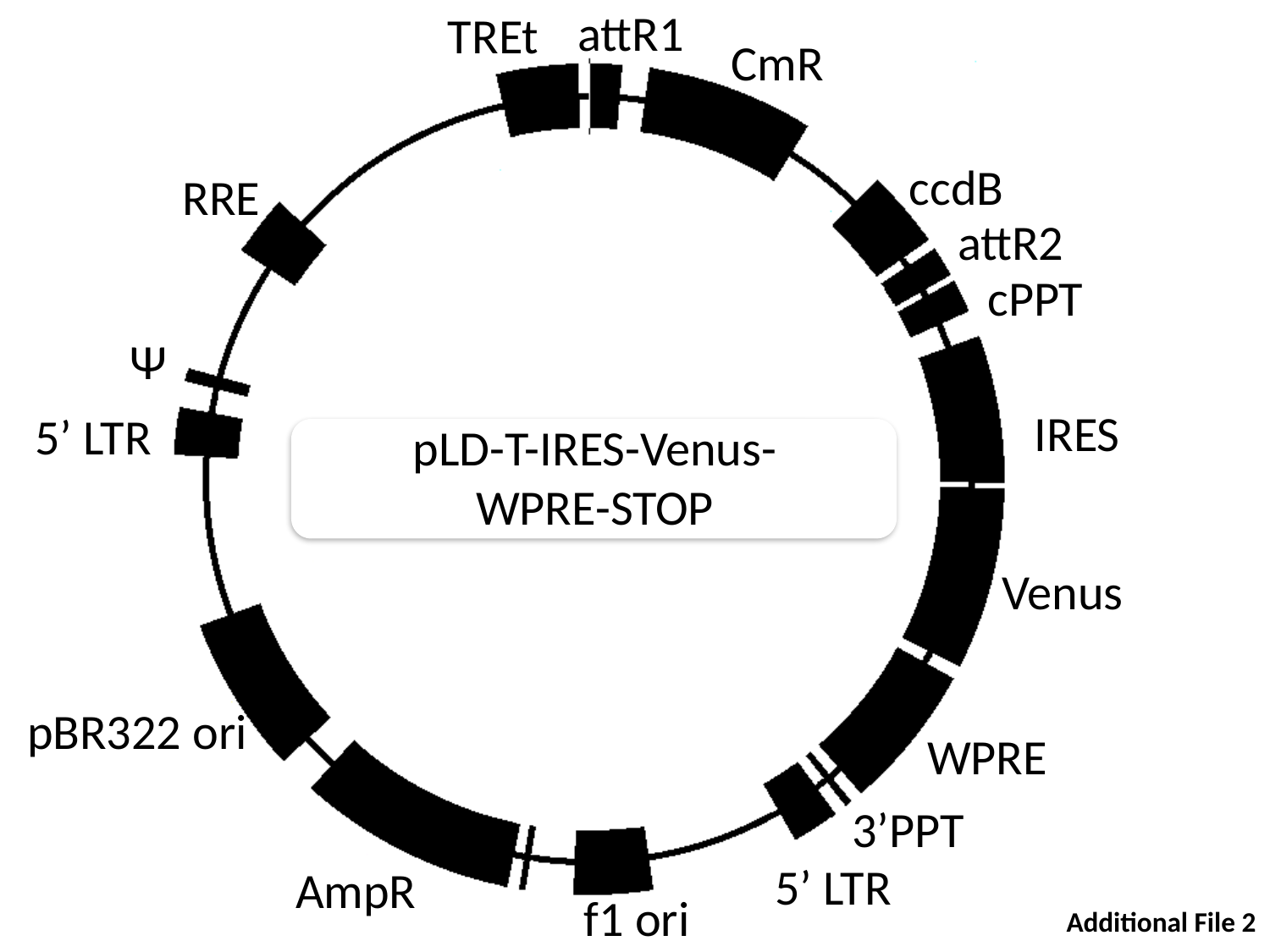

attR1
TREt
CmR
ccdB
RRE
attR2
cPPT
Ψ
IRES
5’ LTR
pLD-T-IRES-Venus-WPRE-STOP
Venus
pBR322 ori
WPRE
3’PPT
5’ LTR
AmpR
f1 ori
Additional File 2
