# Supplementary figures and images for: A genome scale overexpression screen to reveal drug activity in human cells
Source: Genome Med. 2014 Apr 29;6(4):32. doi: 10.1186/gm549 (PMC4062067; doi:10.1186/gm549)

## Slide 1
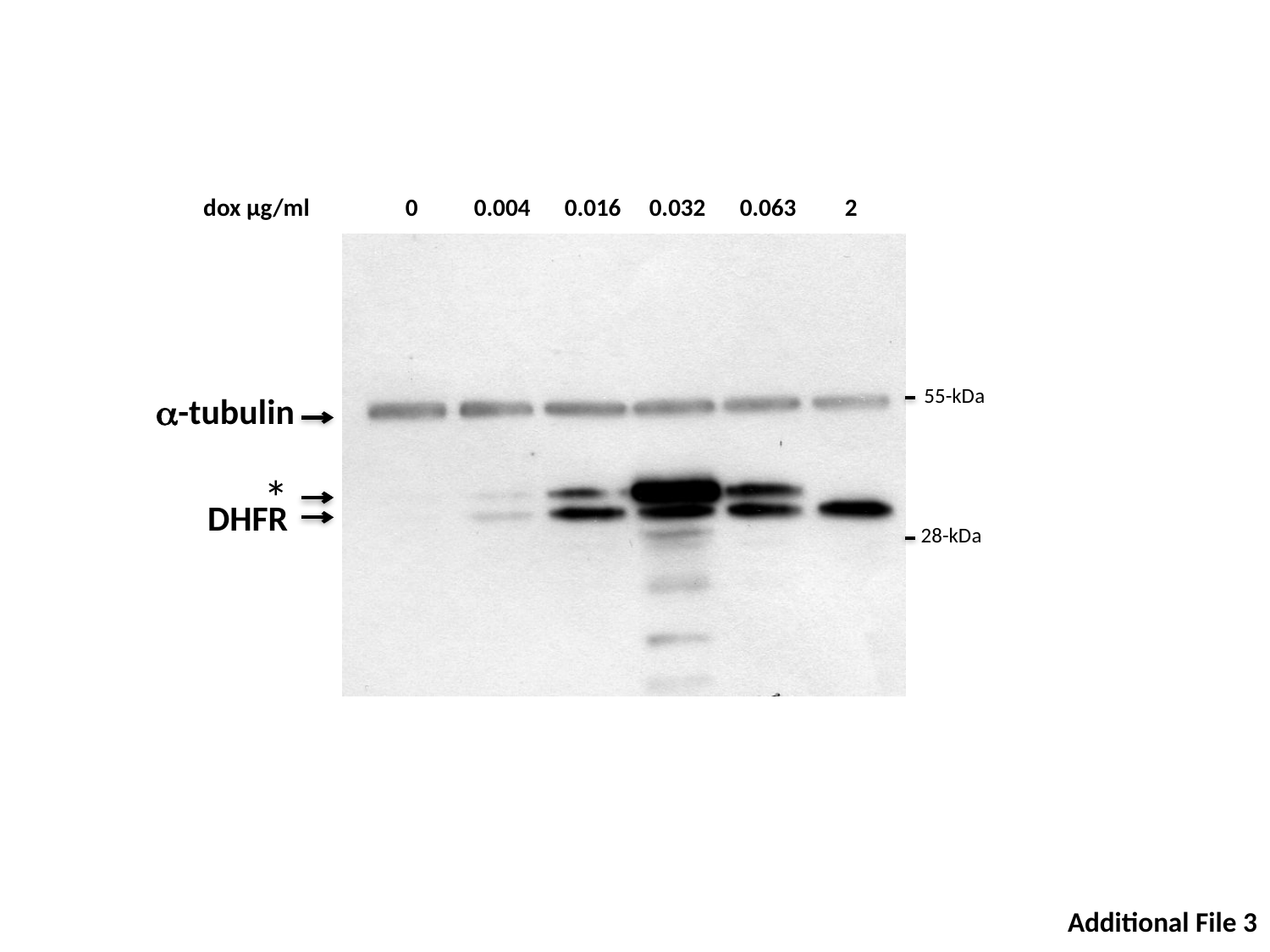

dox μg/ml
0
0.004
0.016
0.032
0.063
2
55-kDa
α-tubulin
*
DHFR
28-kDa
Additional File 3

Supplement: Additional file 3 — Original immunoblotting showing the conditional expression of DHFR in HEK293_M2 cells. Addition of 4 ng/ml, 16 ng/ml, 32 ng/ml, 63 ng/ml and 2,000 ng/ml of doxycycline induced DHFR protein expression in the stable cell line HEK293_M2. Expression level was compared to alpha tubulin as a loading control. The asterisk marks a human protein that cross-reacts with the anti-DHFR antibody. [file gm549-S3.pptx]

## Slide 1
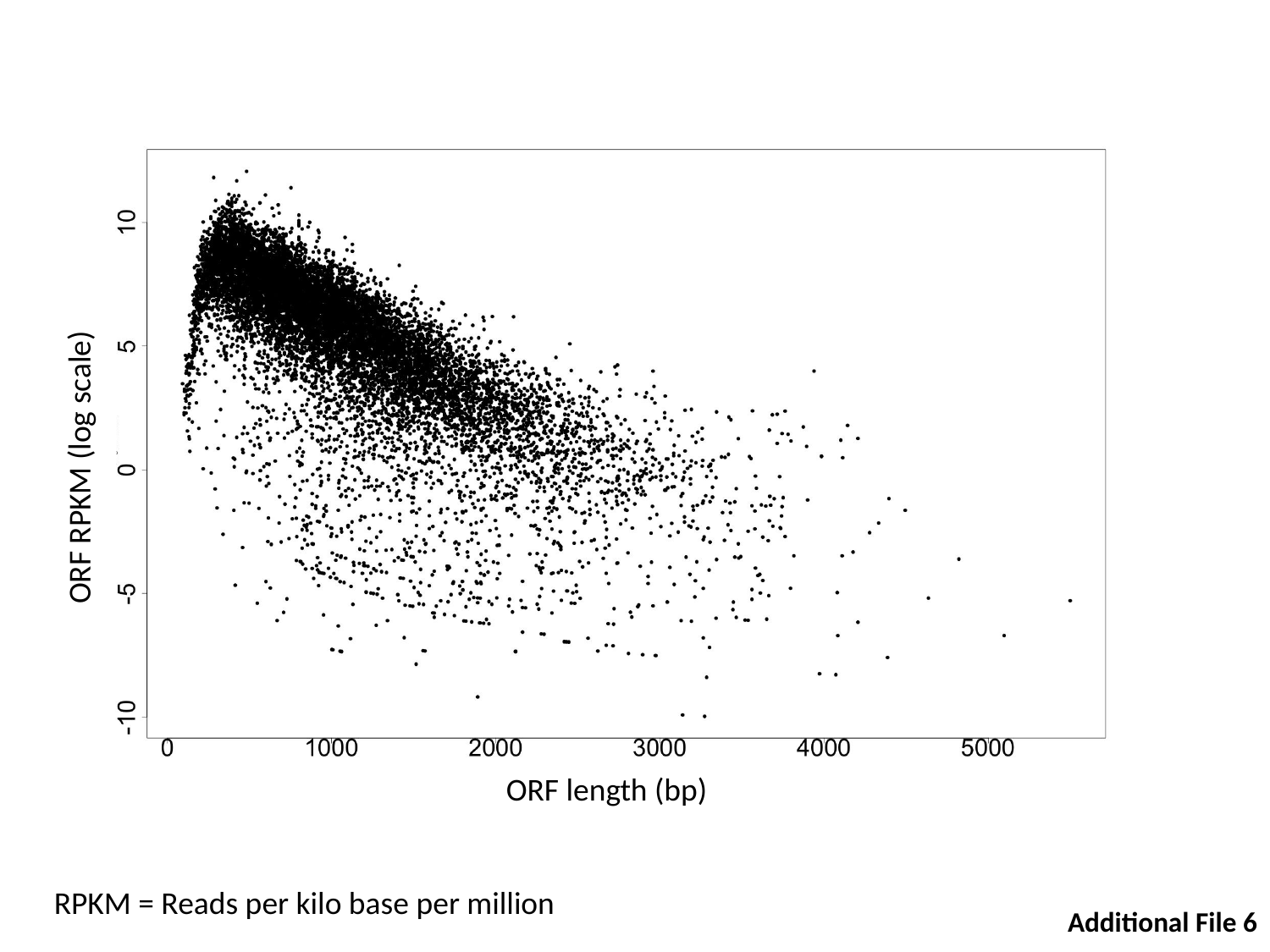

ORF RPKM (log scale)
ORF length (bp)
RPKM = Reads per kilo base per million
Additional File 6

Supplement: Additional file 6 — Human ORFeome relative abundance in HEK293_M2s at T0. [file gm549-S6.pptx]

## Slide 1
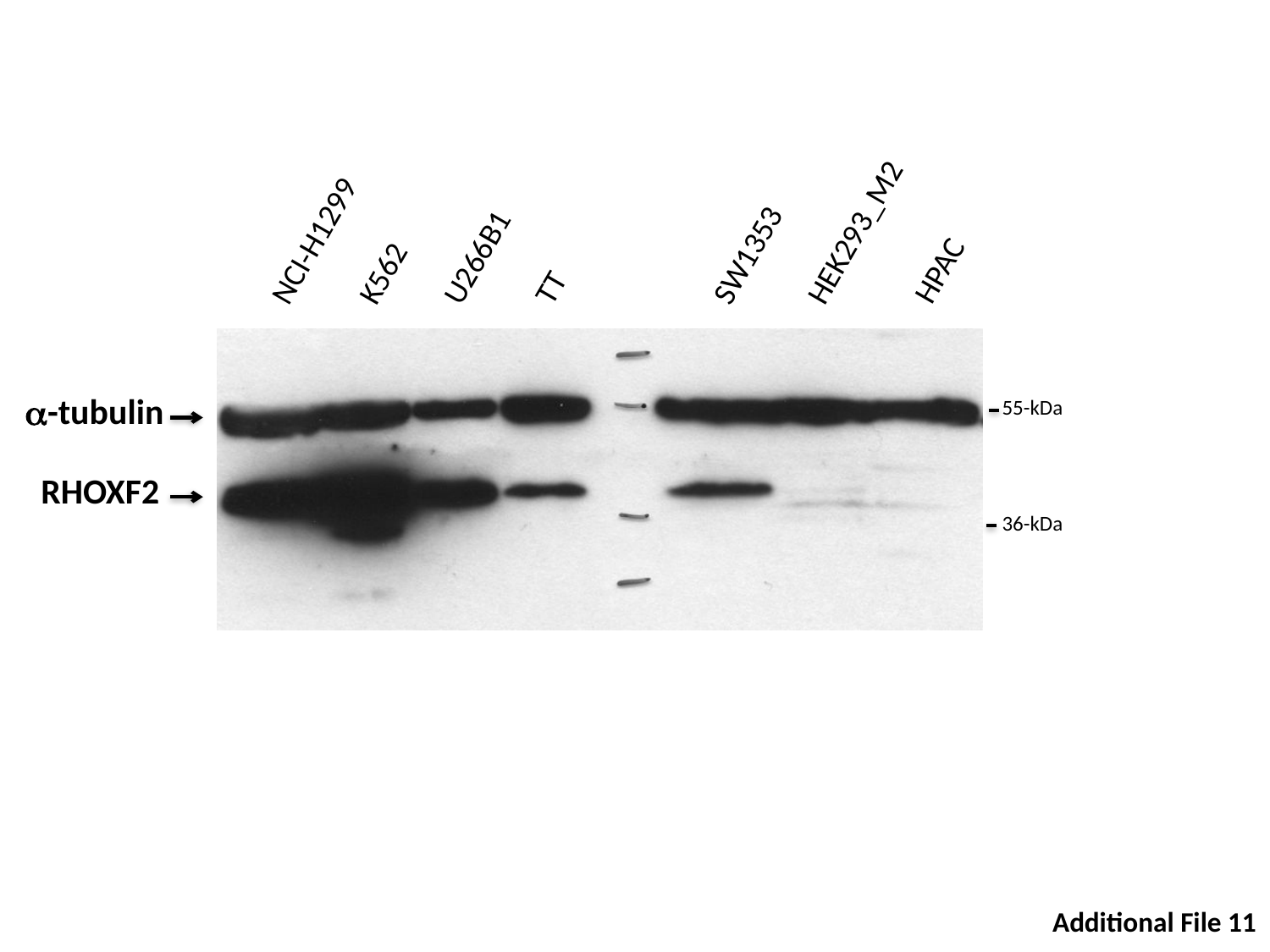

HEK293_M2
NCI-H1299
SW1353
U266B1
HPAC
K562
TT
α-tubulin
55-kDa
RHOXF2
36-kDa
Additional File 11

Supplement: Additional file 11 — RHOXF2 was detected in various tumor cell lines by western blotting. [file gm549-S11.pptx]
