# Supplementary material for: A genome scale overexpression screen to reveal drug activity in human cells
Source: Genome Med. 2014 Apr 29;6(4):32. doi: 10.1186/gm549 (PMC4062067; doi:10.1186/gm549)
Supplement: Additional file 4 — DHFR was identified as the predominant gene in this pool of 376 hORFs whose overexpression provided resistance to methotrexate. HEK293_M2 cells harboring a minipool collection of 376 hORFs (including DHFR) were grown in the presence of a lethal dose of methotrexate. The nature of the hORFs conferring resistance to the drug was identified by plotting the log2 of the signal intensity for each hORF in the cells cultured in the presence of methotrexate on the x-axis; and by plotting the log2 ratio of the signal intensity for each hORF of the cells cultured in presence of the drug divided by the signal intensity for each hORF of the cells grown in presence of DMSO on the y-axis. [file gm549-S4.pptx]

## Slide 1
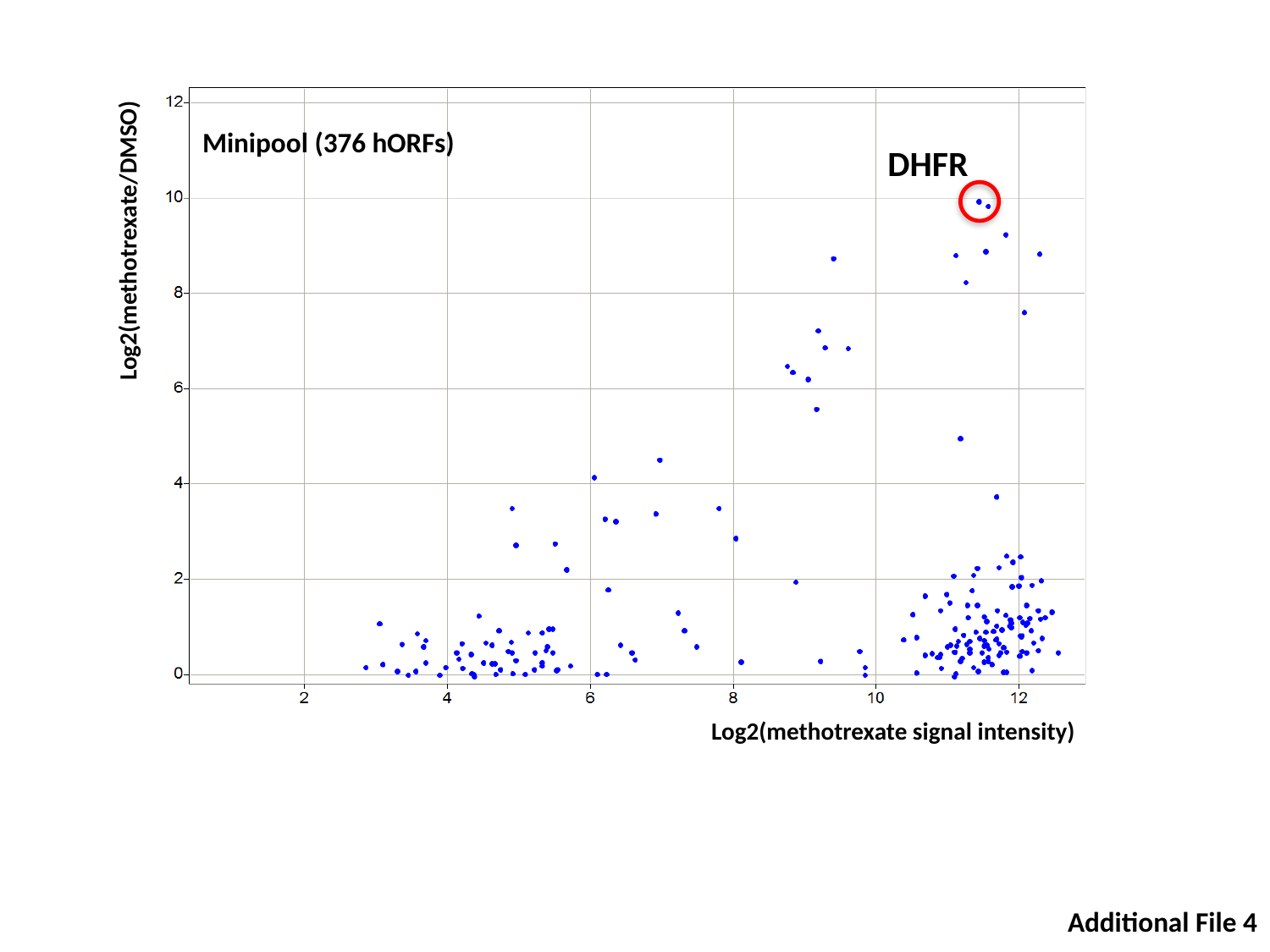

Minipool (376 hORFs)
DHFR
Log2(methotrexate/DMSO)
Log2(methotrexate signal intensity)
Additional File 4
