# Supplementary material for: A genome scale overexpression screen to reveal drug activity in human cells
Source: Genome Med. 2014 Apr 29;6(4):32. doi: 10.1186/gm549 (PMC4062067; doi:10.1186/gm549)
Supplement: Additional file 5 — Detection of Venus-expressing HEK293_M2 cells after 12, 24 and 48 hours of induction. (a,b) HEK293_M2 cells stably transduced with the hORFeome collection (b) or not (a) were cultured in the presence of doxycycline (2 μg/ml) for 12, 24 and 48 hours. After gene induction, cell number and intensity of the Venus fluorescence were measured by flow cytometry. The percentage of Venus positive cells was compared to the total number of living cells measured. [file gm549-S5.ppt]

## Slide 1
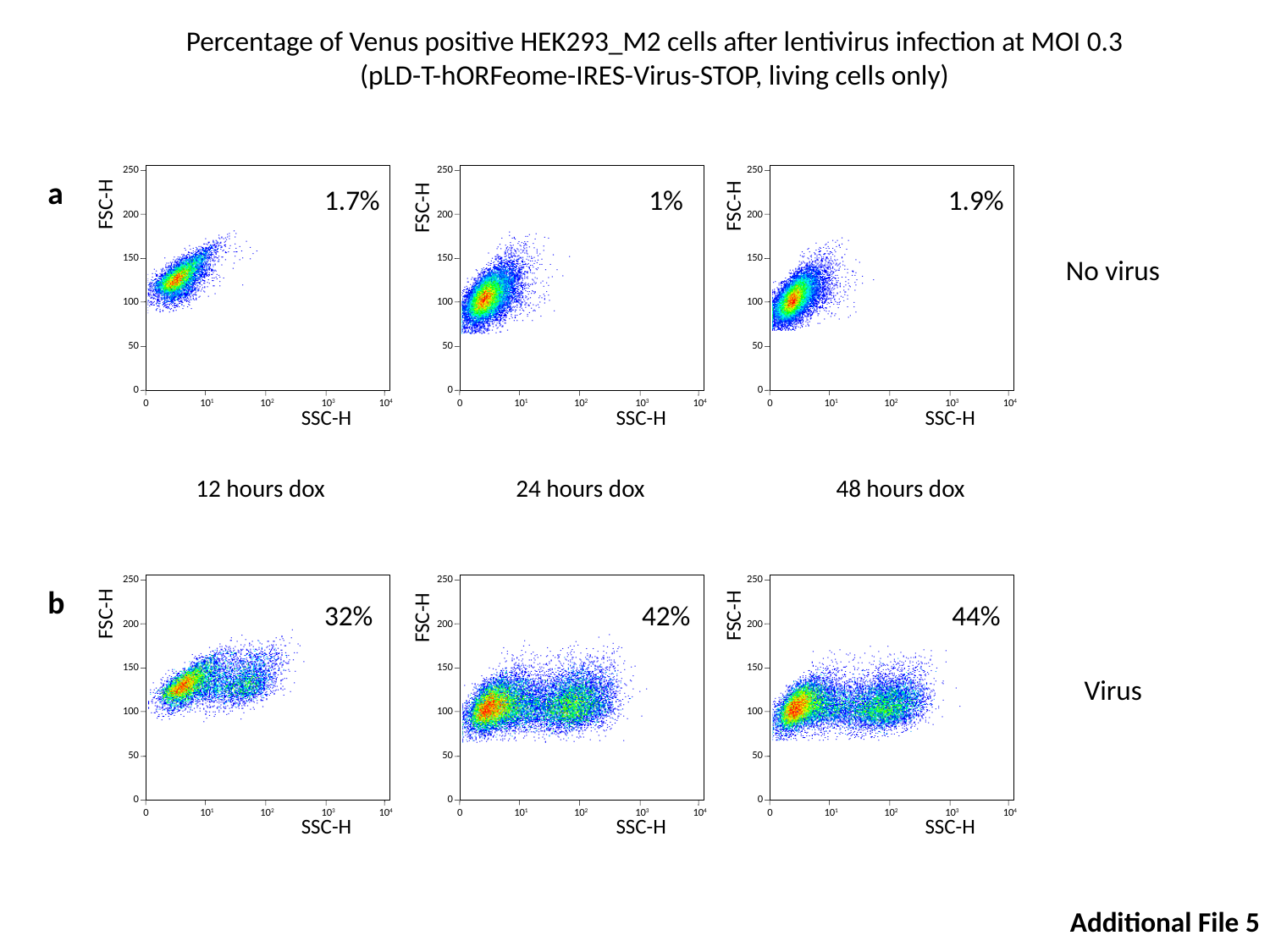

Percentage of Venus positive HEK293_M2 cells after lentivirus infection at MOI 0.3 (pLD-T-hORFeome-IRES-Virus-STOP, living cells only)
250
200
150
100
50
0
0
101
102
103
104
250
200
150
100
50
0
0
101
102
103
104
250
200
150
100
50
0
0
101
102
103
104
a
1.7%
1%
1.9%
FSC-H
FSC-H
FSC-H
No virus
SSC-H
SSC-H
SSC-H
12 hours dox
24 hours dox
48 hours dox
250
200
150
100
50
0
0
101
102
103
104
250
200
150
100
50
0
0
101
102
103
104
250
200
150
100
50
0
0
101
102
103
104
b
32%
42%
44%
FSC-H
FSC-H
FSC-H
Virus
SSC-H
SSC-H
SSC-H
Additional File 5
