# Supplementary material for: A genome scale overexpression screen to reveal drug activity in human cells
Source: Genome Med. 2014 Apr 29;6(4):32. doi: 10.1186/gm549 (PMC4062067; doi:10.1186/gm549)
Supplement: Additional file 7 — Plasmid map of the transposon-based vector PB-TGcMV-Neo. [file gm549-S7.pptx]

## Slide 1
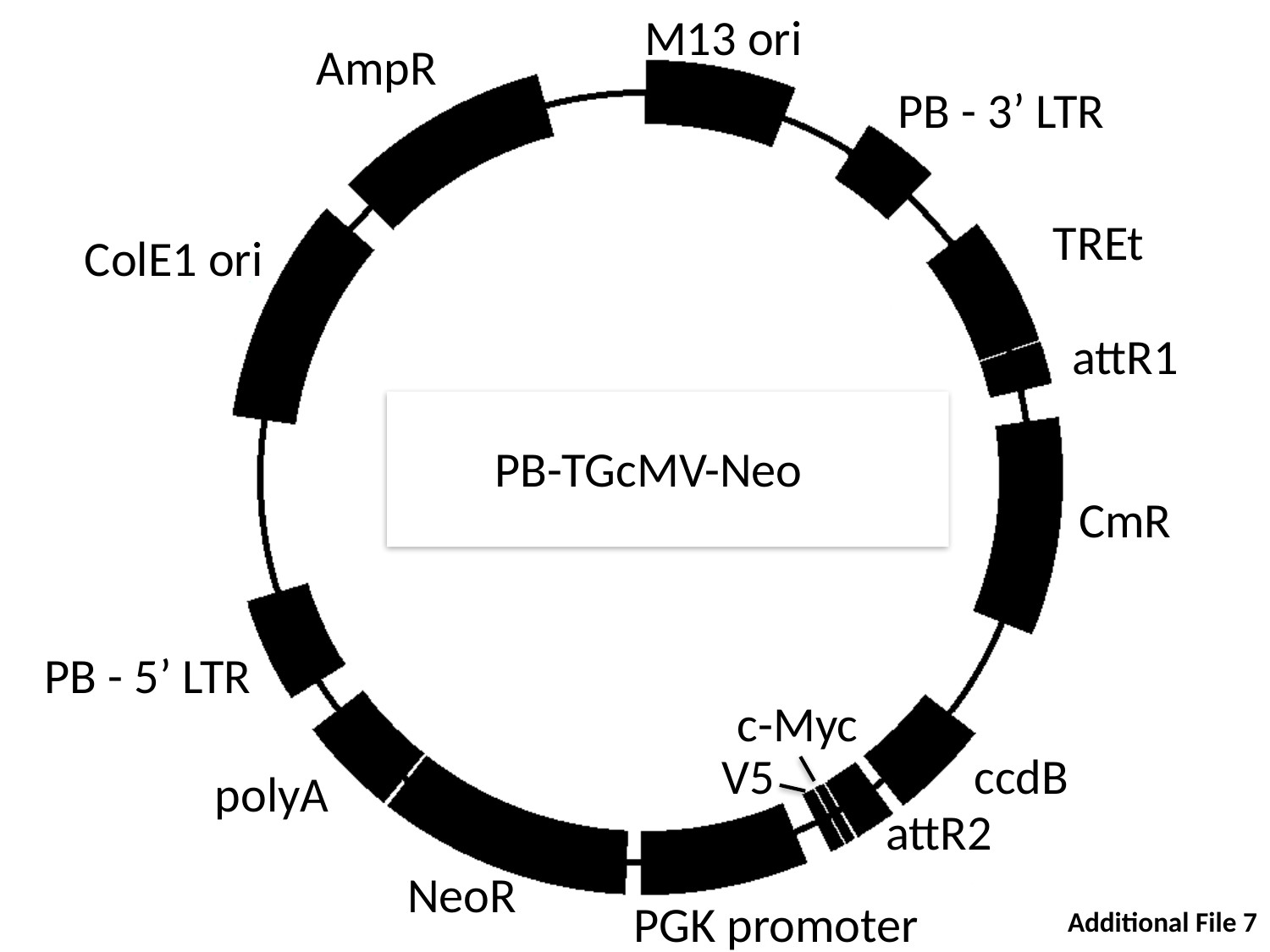

M13 ori
AmpR
PB - 3’ LTR
TREt
ColE1 ori
attR1
PB-TGcMV-Neo
CmR
PB - 5’ LTR
c-Myc
V5
ccdB
polyA
attR2
NeoR
PGK promoter
Additional File 7
