# Supplementary material for: A genome scale overexpression screen to reveal drug activity in human cells
Source: Genome Med. 2014 Apr 29;6(4):32. doi: 10.1186/gm549 (PMC4062067; doi:10.1186/gm549)
Supplement: Additional file 8 — In HEK293_M2 cells, RHOXF2 overexpression conferred resistance to various DNA damaging agents. Stable RHOXF2 cells was cultured in the presence of an increasing concentration of DNA damaging agents and their growth was compared to the stable cells with the empty vector PB-TGcMV-Neo. The effect of RHOXF2 expression on cell viability was measured two days after drug exposure and compared to cells cultured in the absence of drug as a 100% viability control. TukeyHSD test, P < 0.05; *significant difference between RHOXF2 vector with and without doxycycline; **significant difference between RHOXF2 vector with doxycycline and empty vector with doxycycline. Error bars represent standard error of the mean (n = 4). [file gm549-S8.pdf]

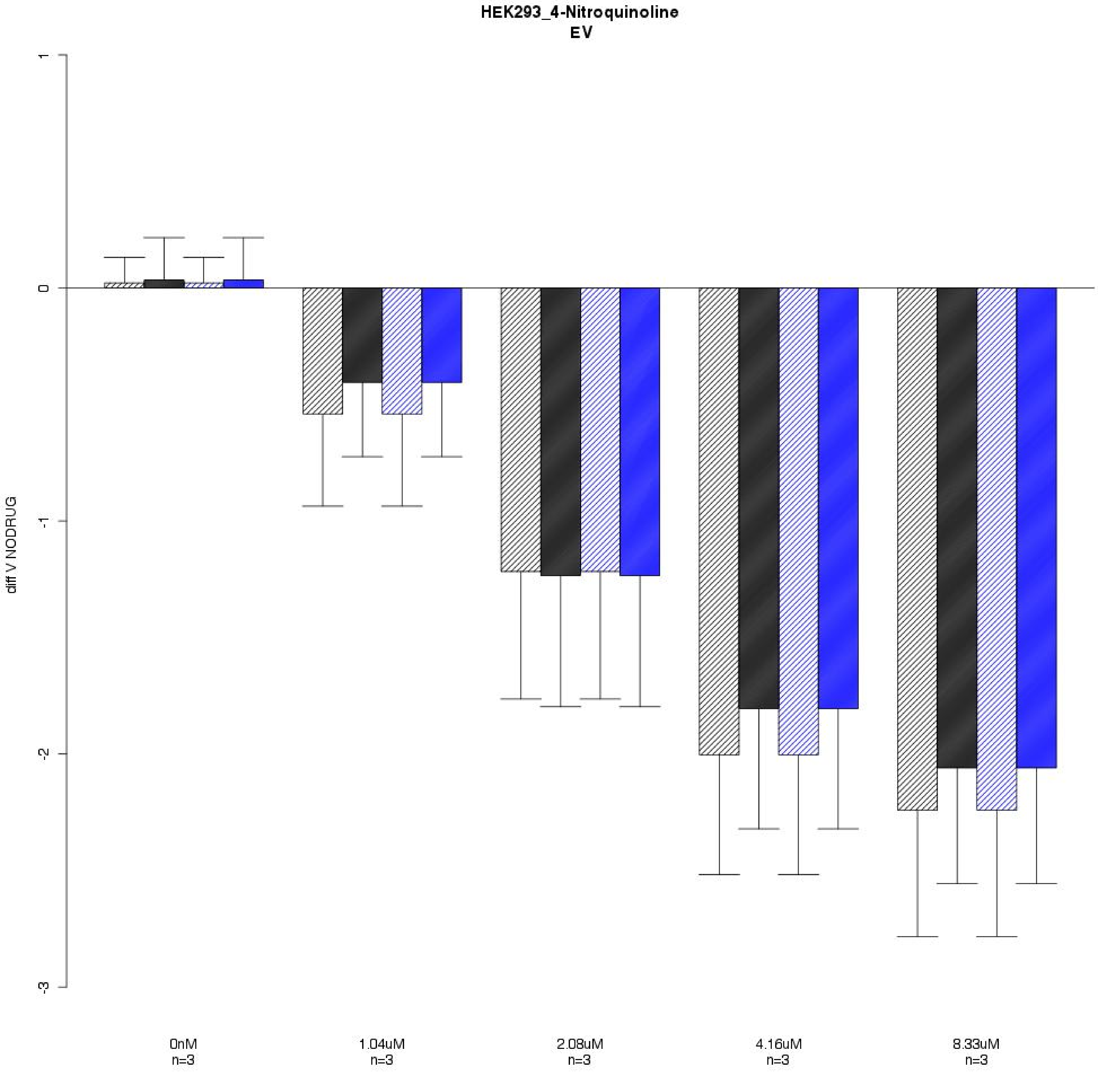

HEK293_4-Nitroquinoline
EV
diff V NODRUG
1
0
-1
-2
-3
0nM
n=3
1.04uM
n=3
2.08uM
n=3
4.16uM
n=3
8.33uM
n=3

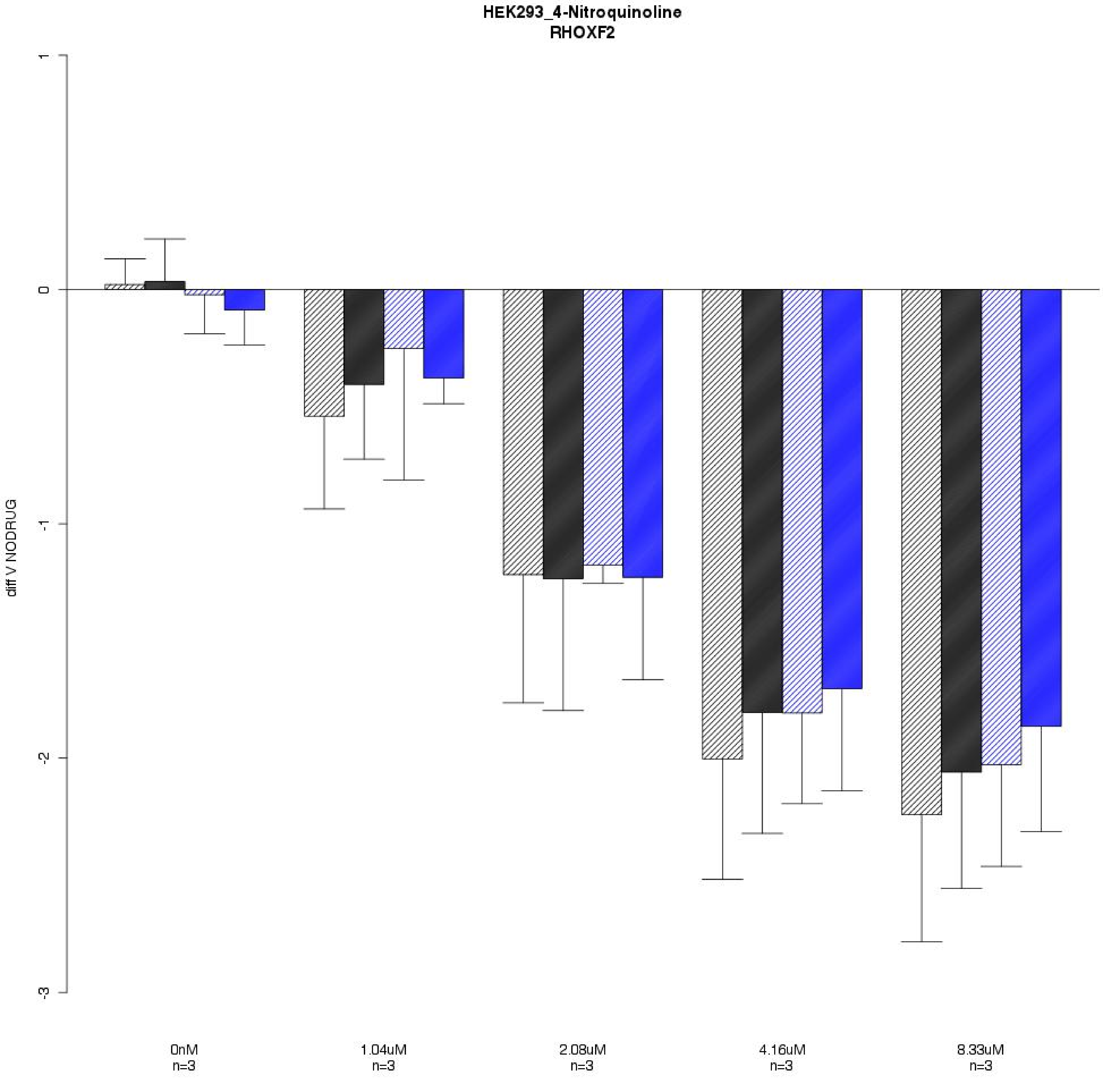
HEK293_4-Nitroquinoline
RHOXF2
diff V NODRUG
1
0
-1
-2
-3
0nM
n=3
1.04uM
n=3
2.08uM
n=3
4.16uM
n=3
8.33uM
n=3

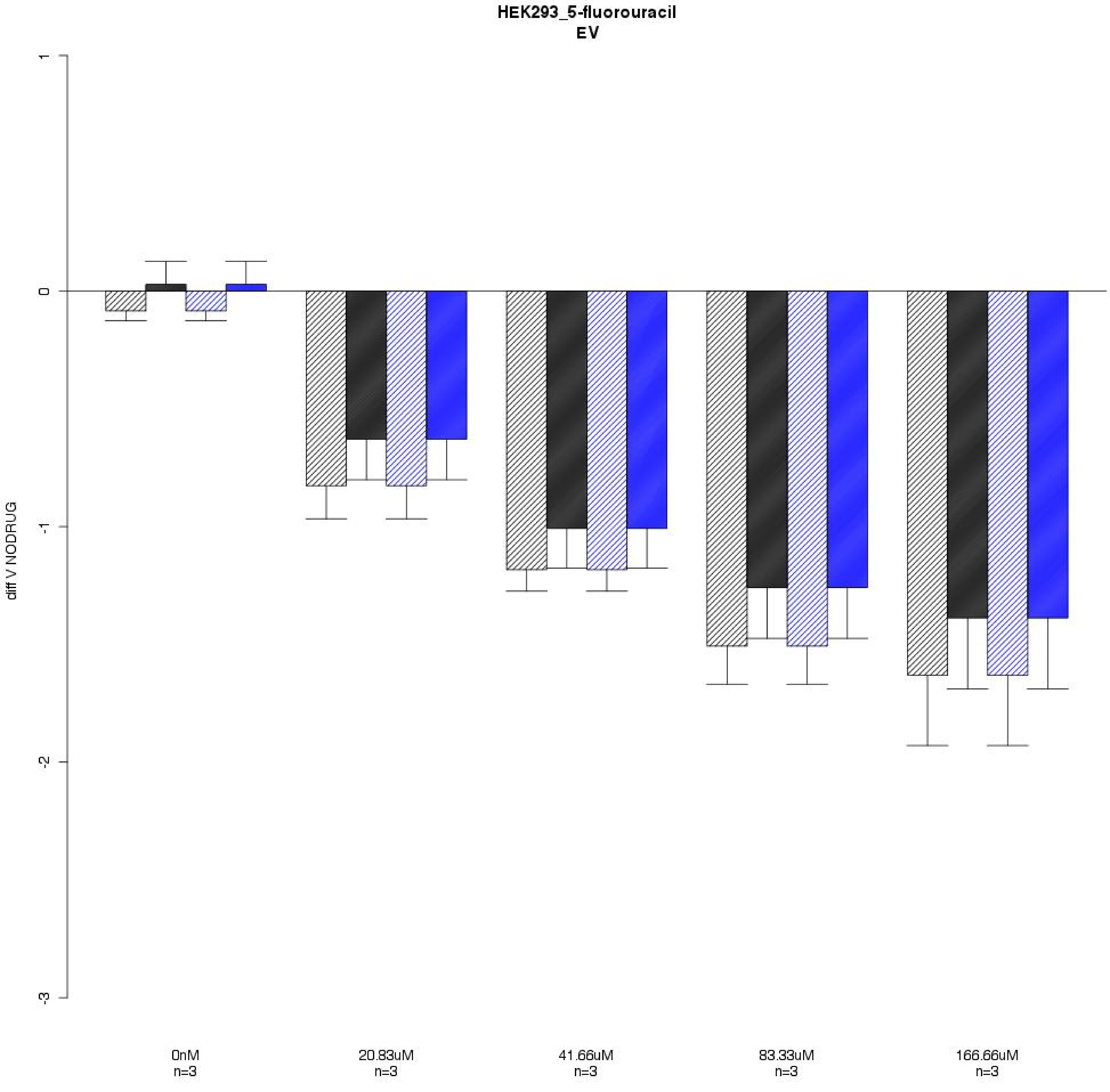
HEK293_5-fluorouracil
EV
diff V NODRUG
1
0
-1
-2
-3
0nM
n=3
20.83uM
n=3
41.66uM
n=3
83.33uM
n=3
166.66uM
n=3

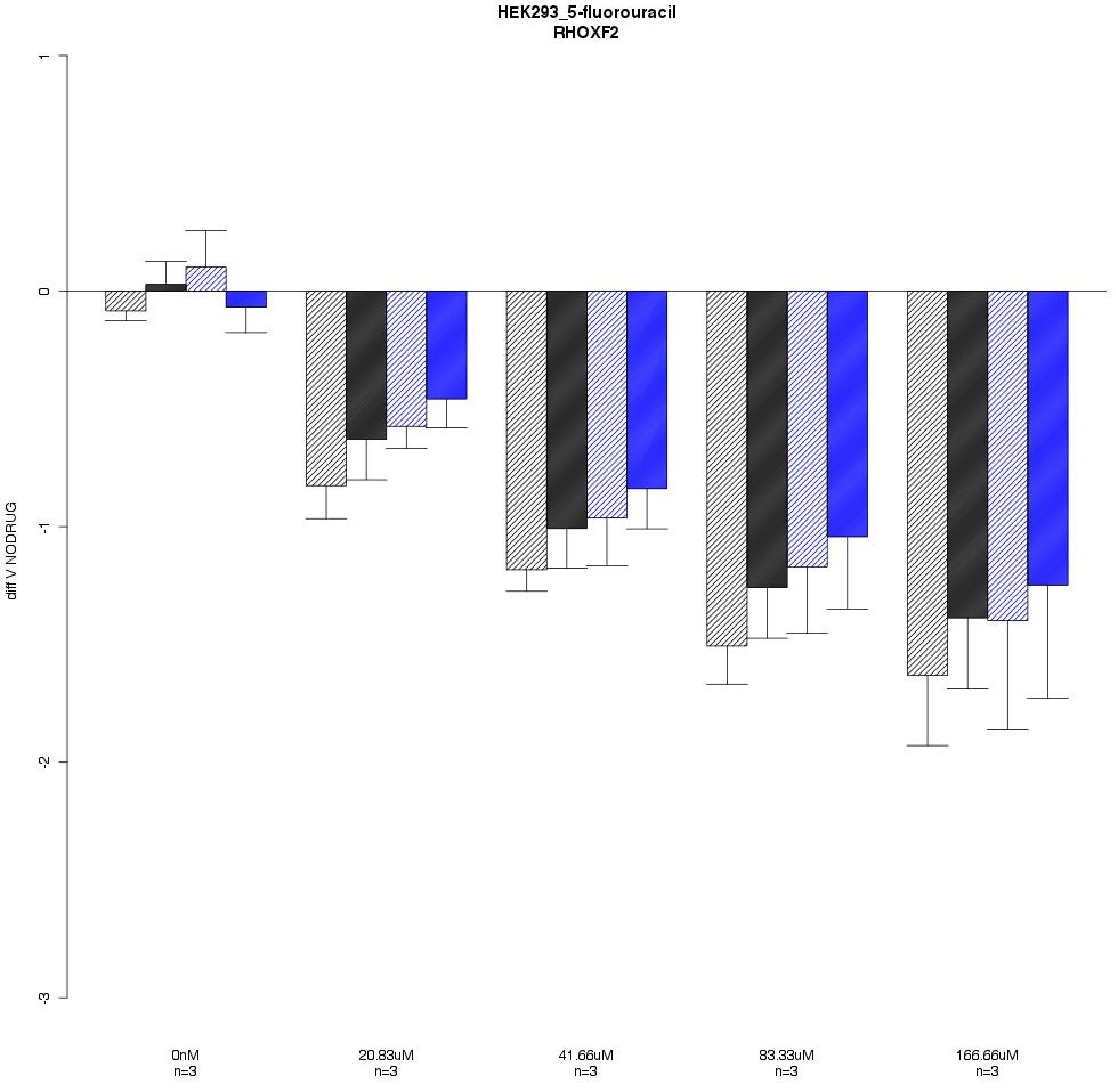
HEK293_5-fluorouracil
RHOXF2
1
0
-1
-2
-3
diff V NODRUG
0nM
n=3
20.83uM
n=3
41.66uM
n=3
83.33uM
n=3
166.66uM
n=3

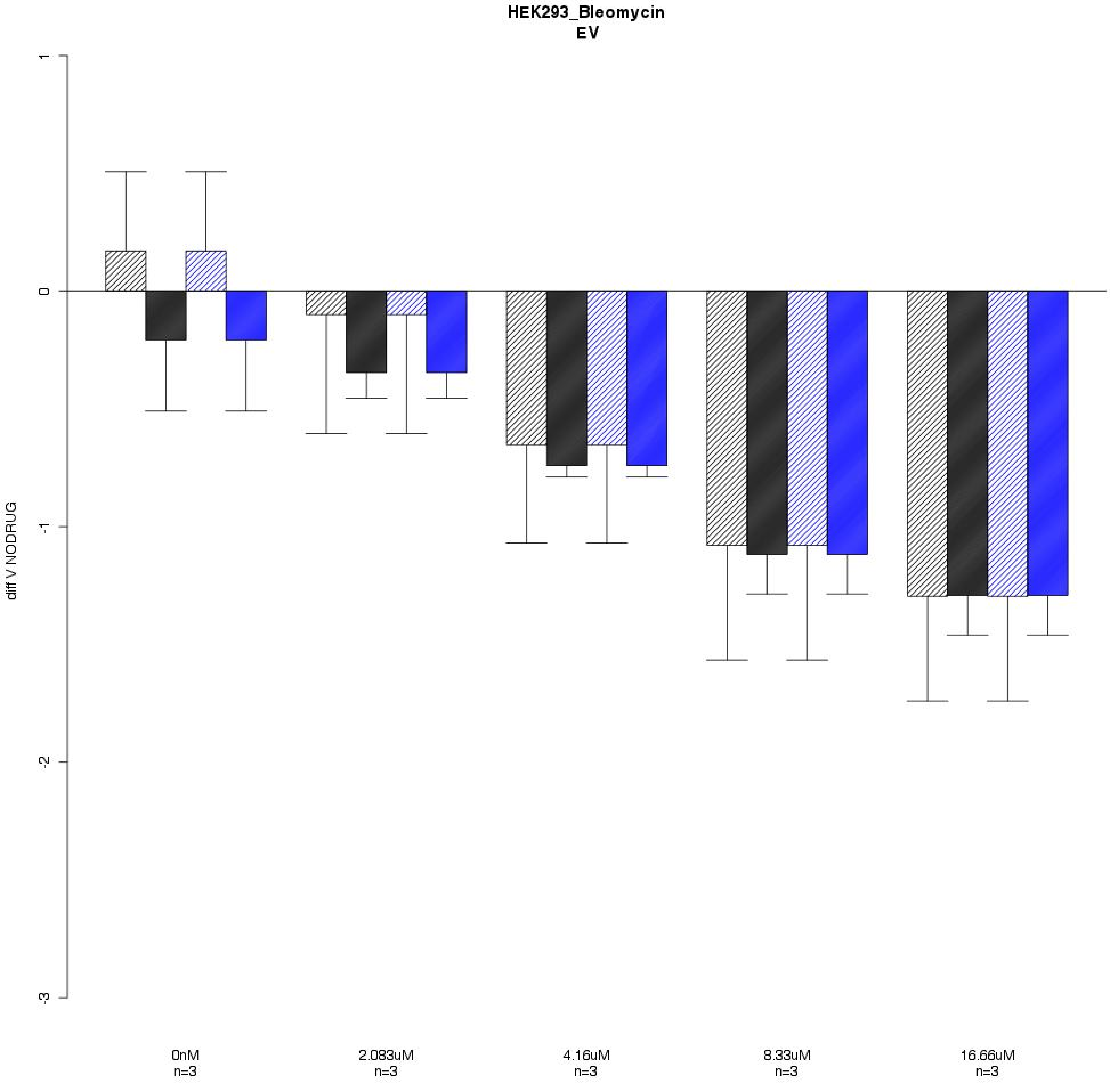

HEK293_Bleomycin
EV
diff V NODRUG
1
0
-1
-2
-3
0nM
n=3
2.083uM
n=3
4.16uM
n=3
8.33uM
n=3
16.66uM
n=3

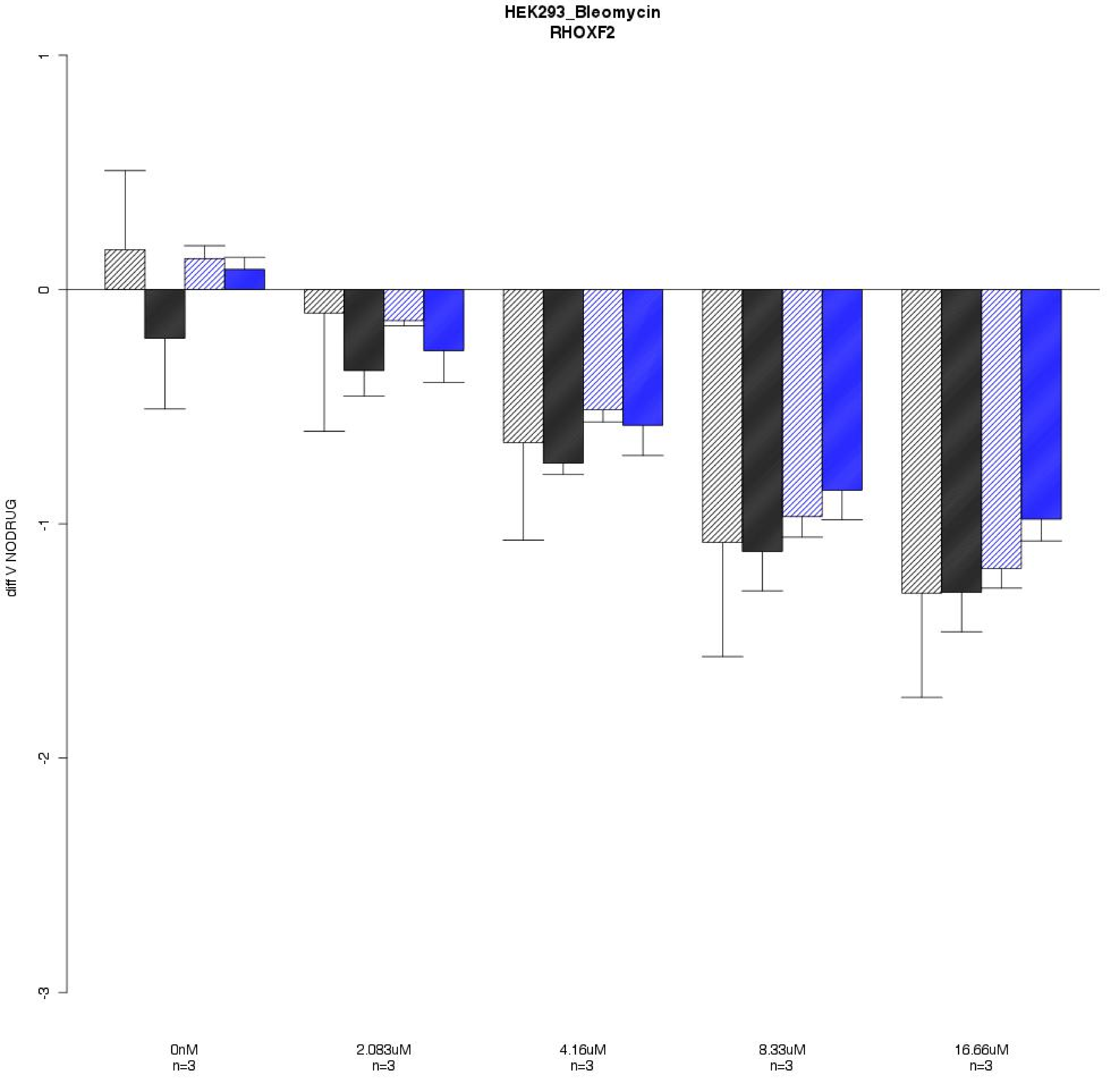

HEK293_Bleomycin
RHOXF2
diff V NODRUG
1
0
-1
-2
-3
0nM
n=3
2.083uM
n=3
4.16uM
n=3
8.33uM
n=3
16.66uM
n=3

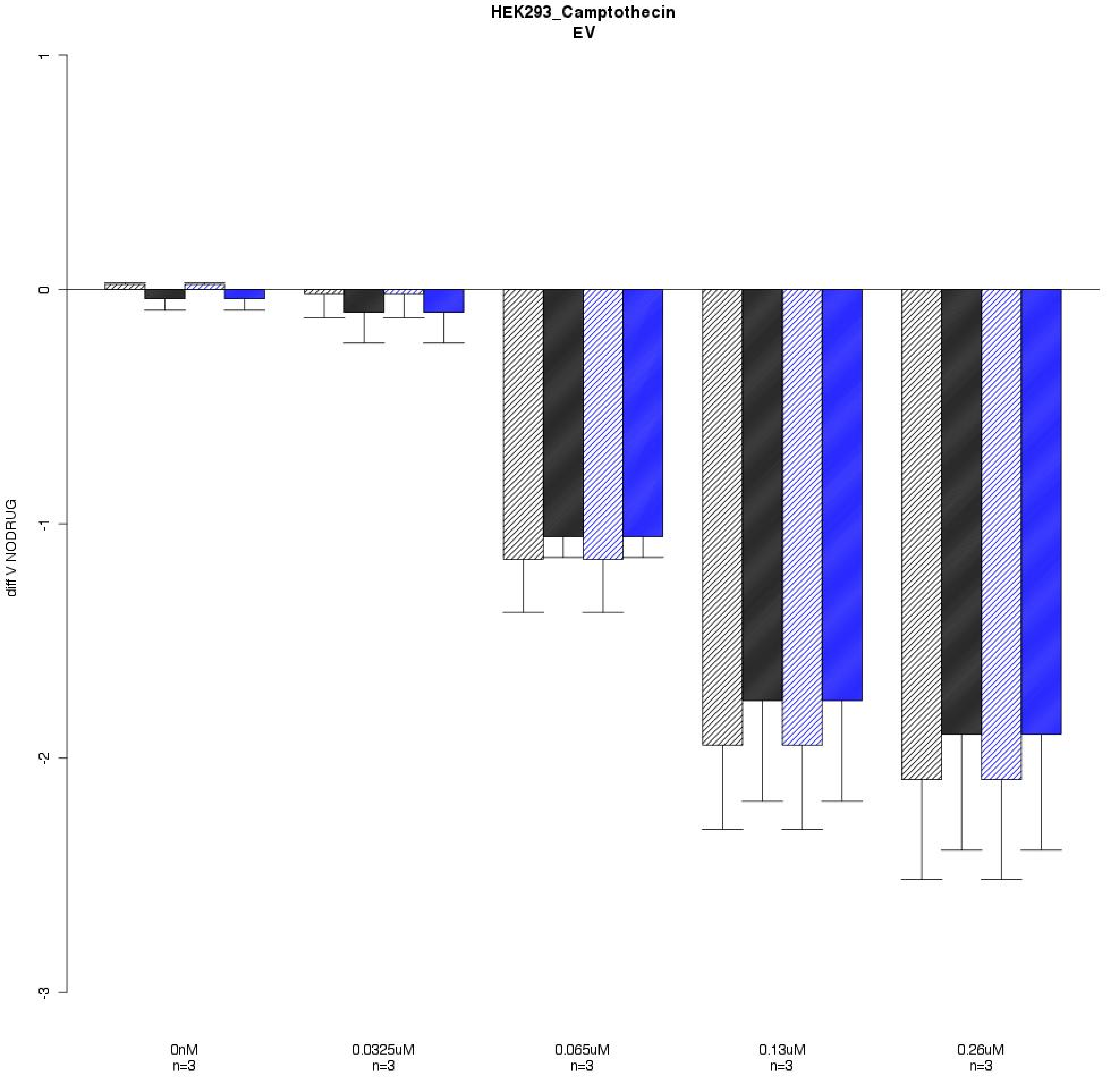
HEK293_Camptothecin
EV
diff V NODRUG
1
0
-1
-2
-3
0nM
n=3
0.0325uM
n=3
0.065uM
n=3
0.13uM
n=3
0.26uM
n=3

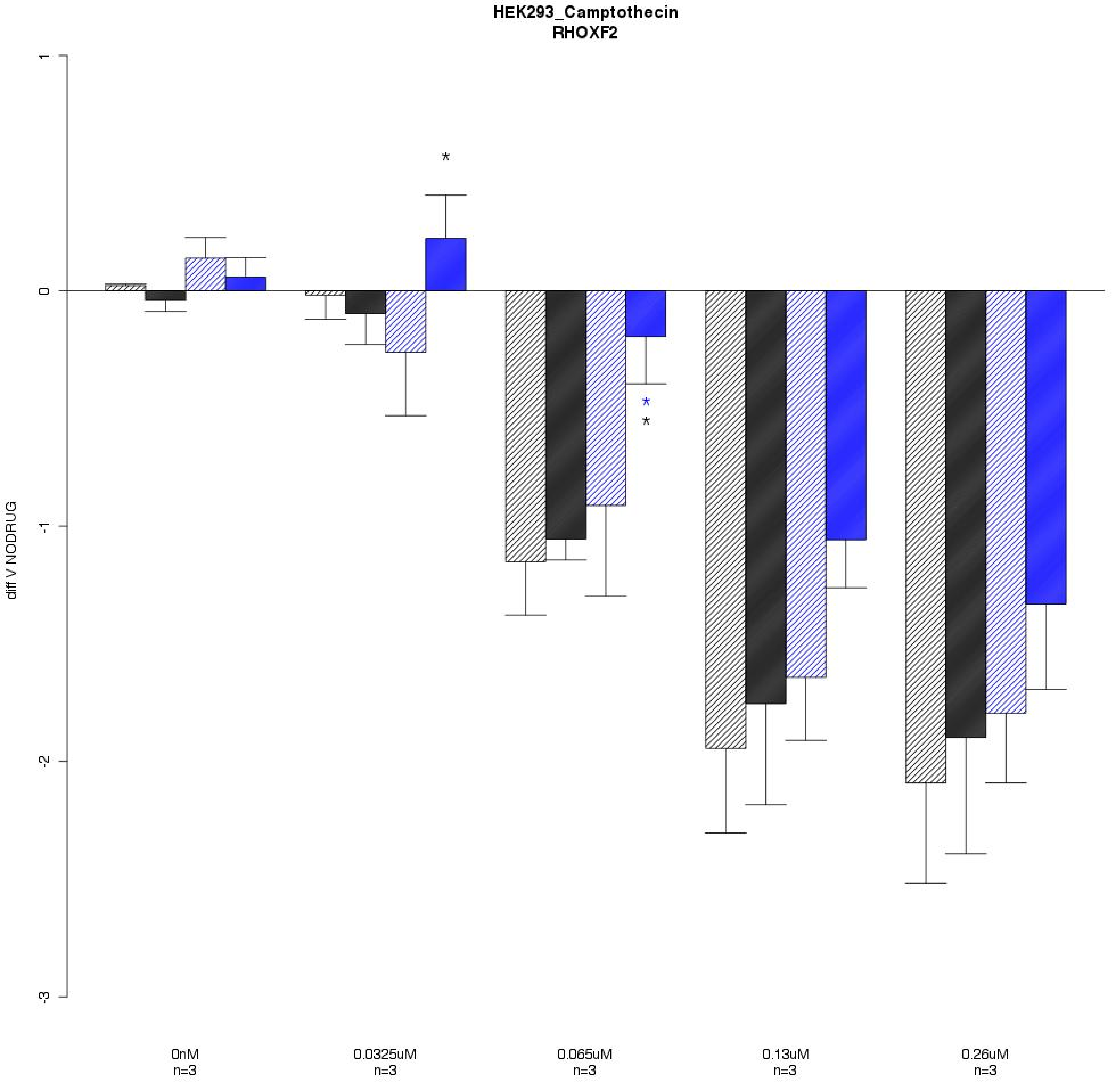
HEK293_Camptothecin
RHOXF2
diff V NODRUG
1
0
-1
-2
-3
*
*
*
0nM
n=3
0.0325uM
n=3
0.065uM
n=3
0.13uM
n=3
0.26uM
n=3

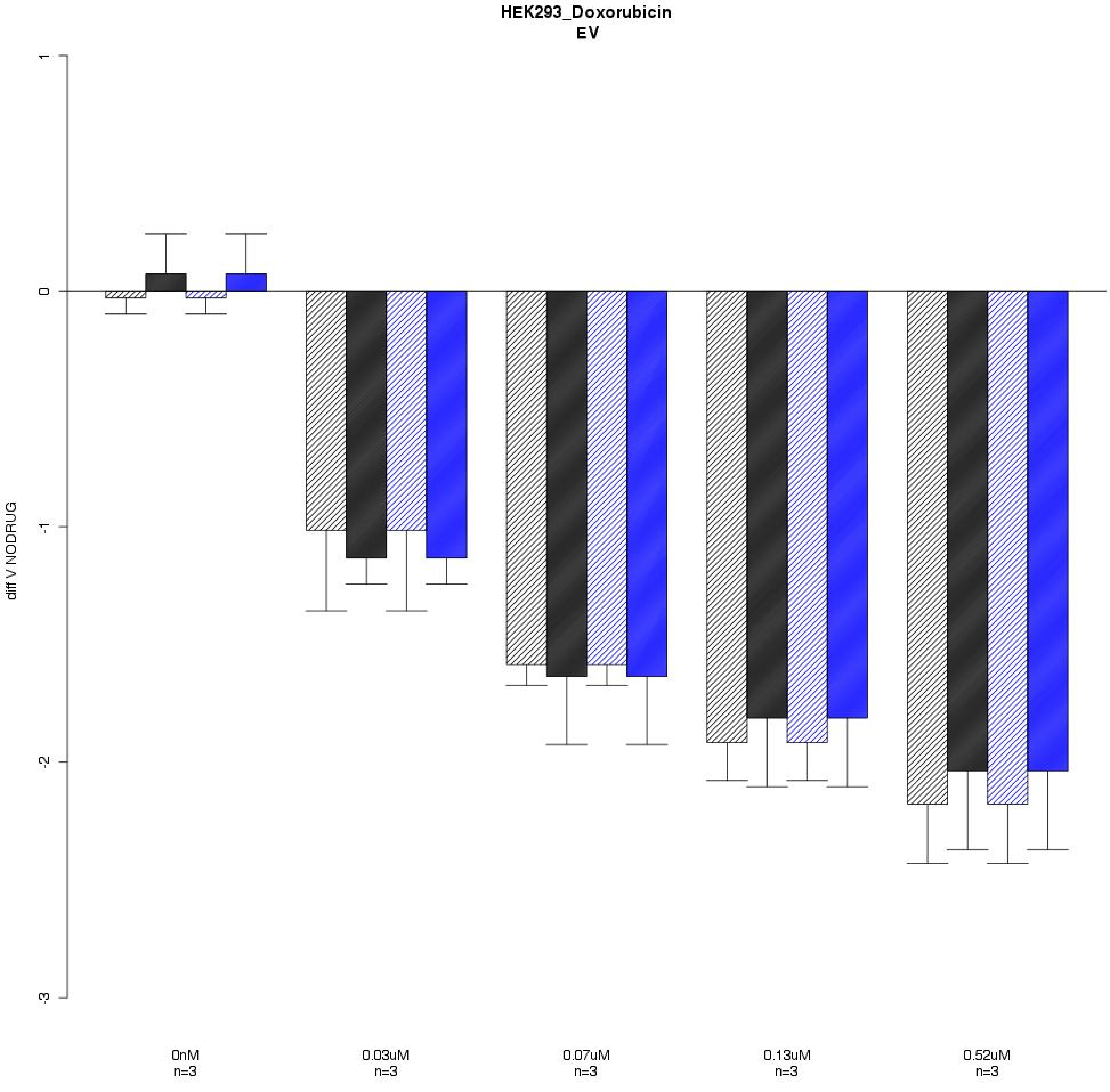

HEK293_Doxorubicin
EV
diff V NODRUG
1
0
-1
-2
-3
0nM
n=3
0.03uM
n=3
0.07uM
n=3
0.13uM
n=3
0.52uM
n=3

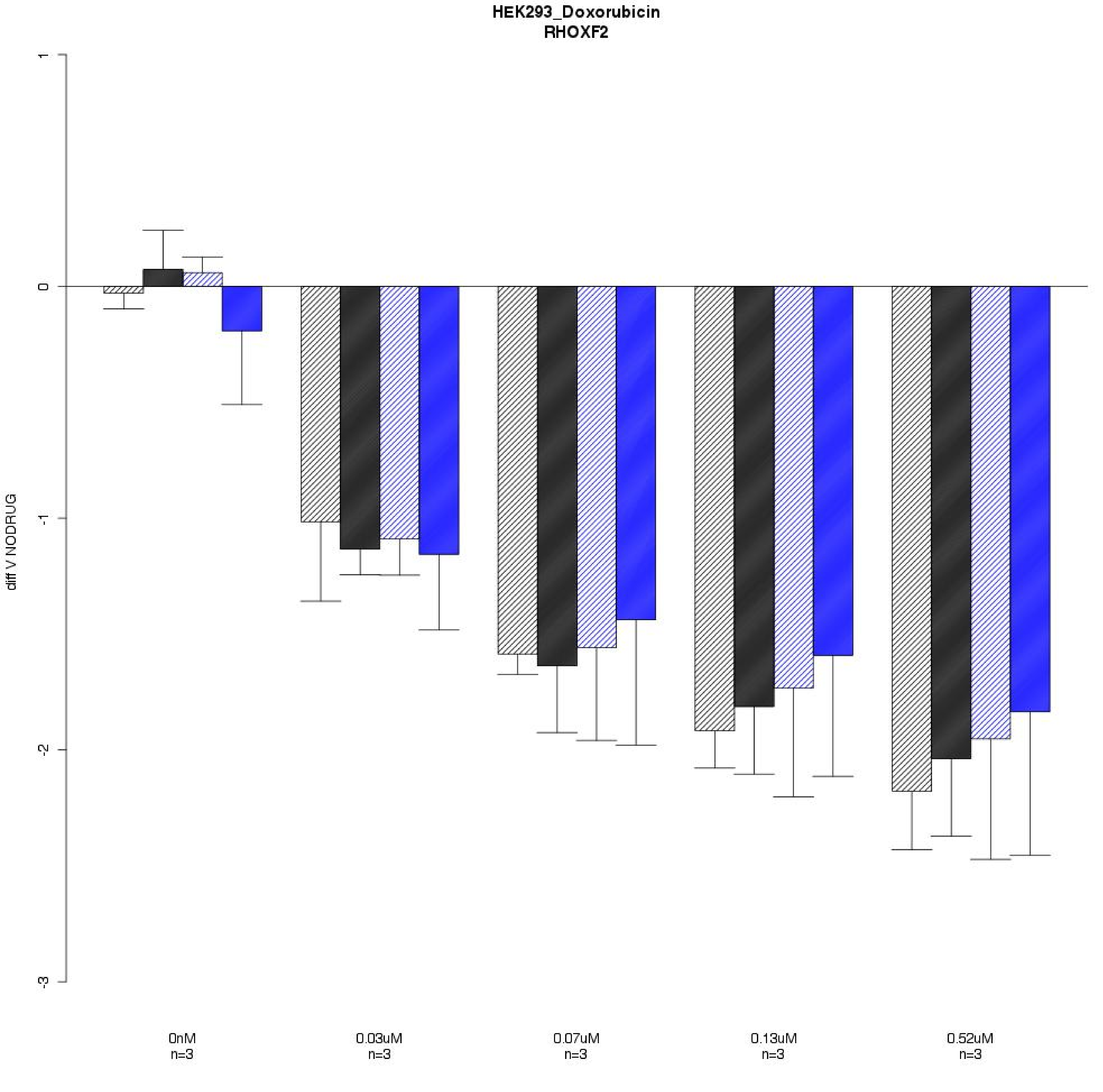

HEK293_Doxorubicin
RHOXF2
diff V NODRUG
1
0
-1
-2
-3
0nM
n=3
0.03uM
n=3
0.07uM
n=3
0.13uM
n=3
0.52uM
n=3

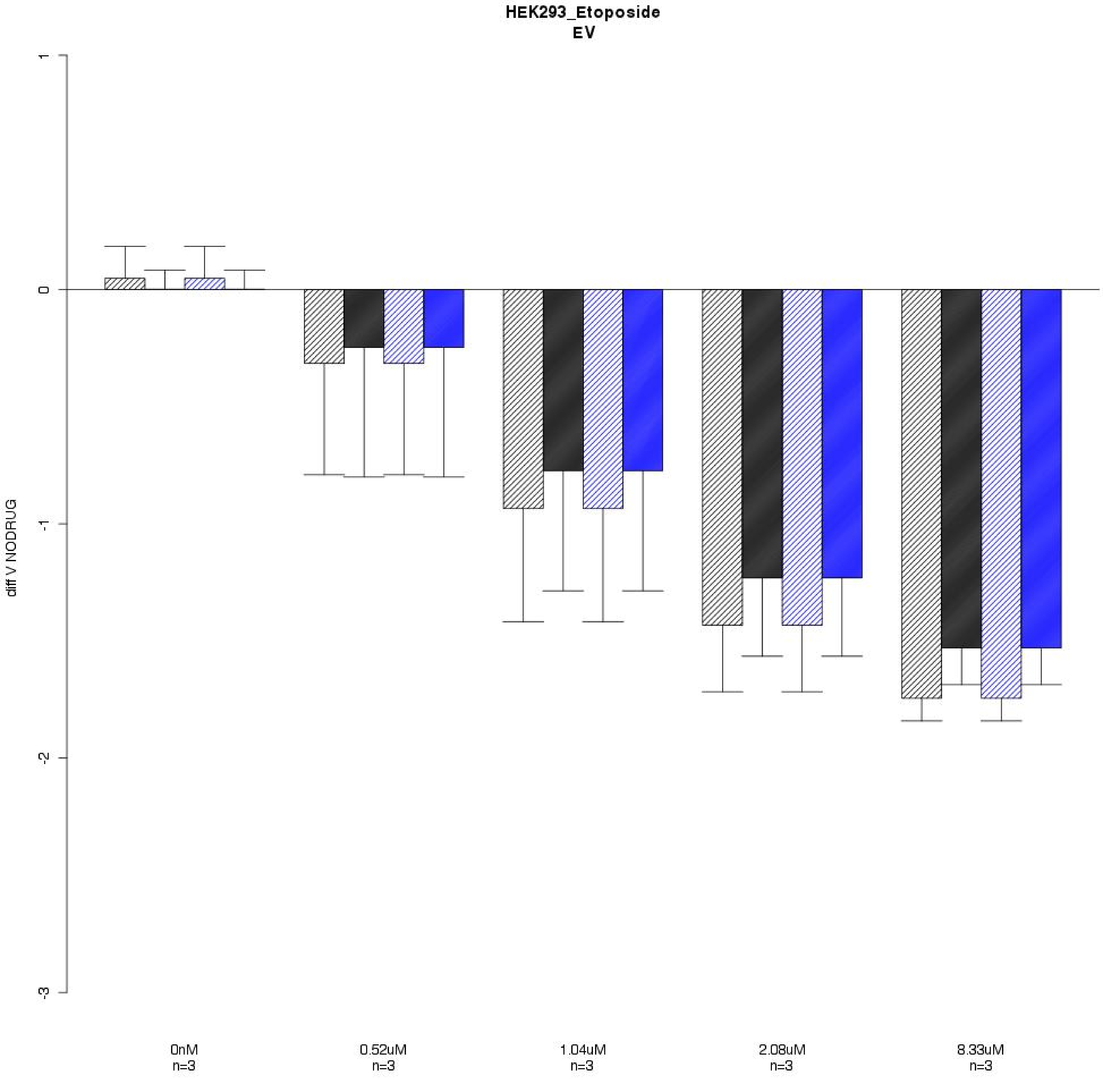
HEK293_Etoposide
EV
diff V NODRUG
1
0
-1
-2
-3
0nM
n=3
0.52uM
n=3
1.04uM
n=3
2.08uM
n=3
8.33uM
n=3

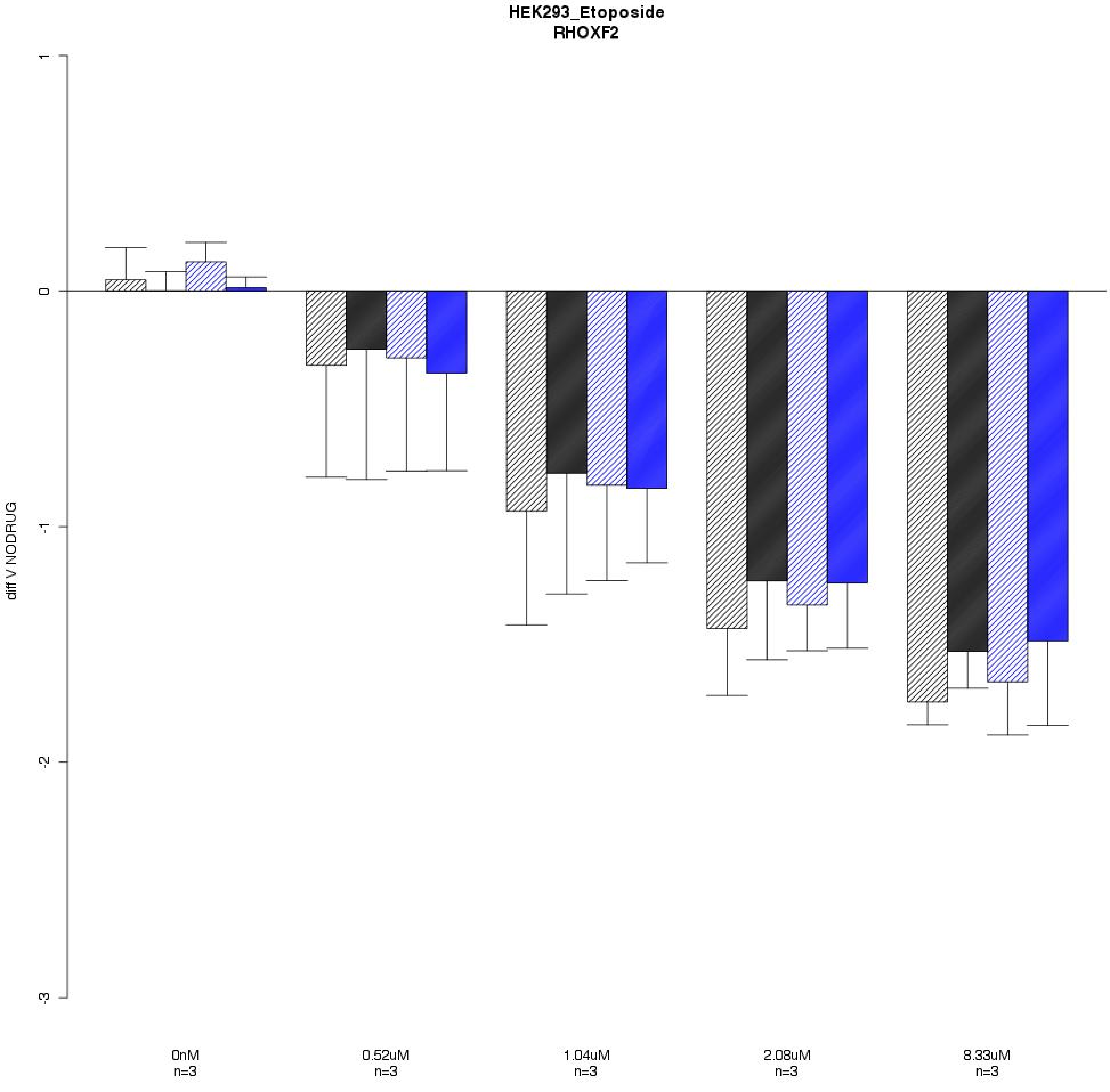
HEK293_Etoposide
RHOXF2
diff V NODRUG
1
0
-1
-2
-3
0nM
n=3
0.52uM
n=3
1.04uM
n=3
2.08uM
n=3
8.33uM
n=3

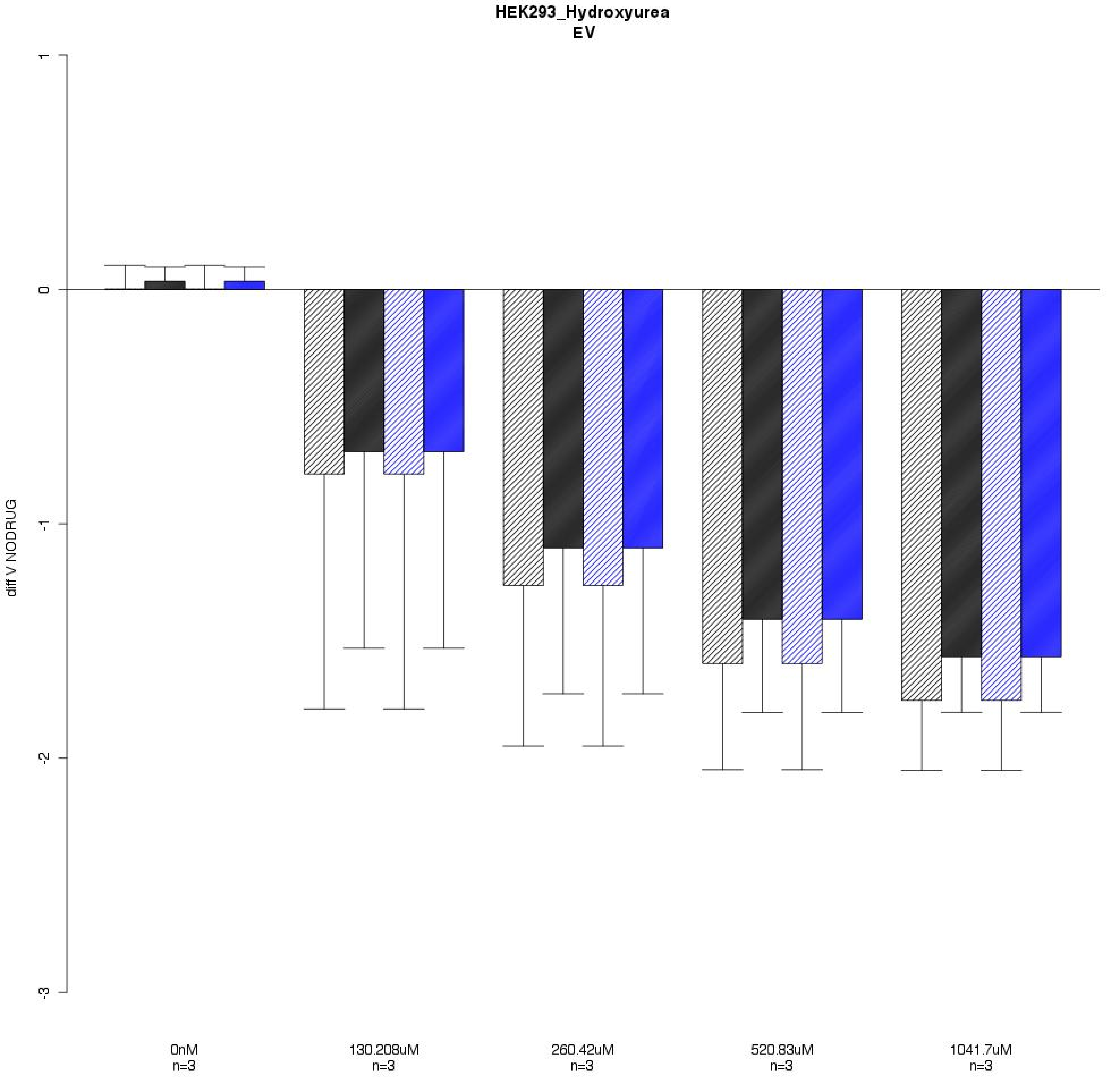
HEK293_Hydroxyurea
EV
diff V NODRUG
1
0
-1
-2
-3
0nM
n=3
130.208uM
n=3
260.42uM
n=3
520.83uM
n=3
1041.7uM
n=3

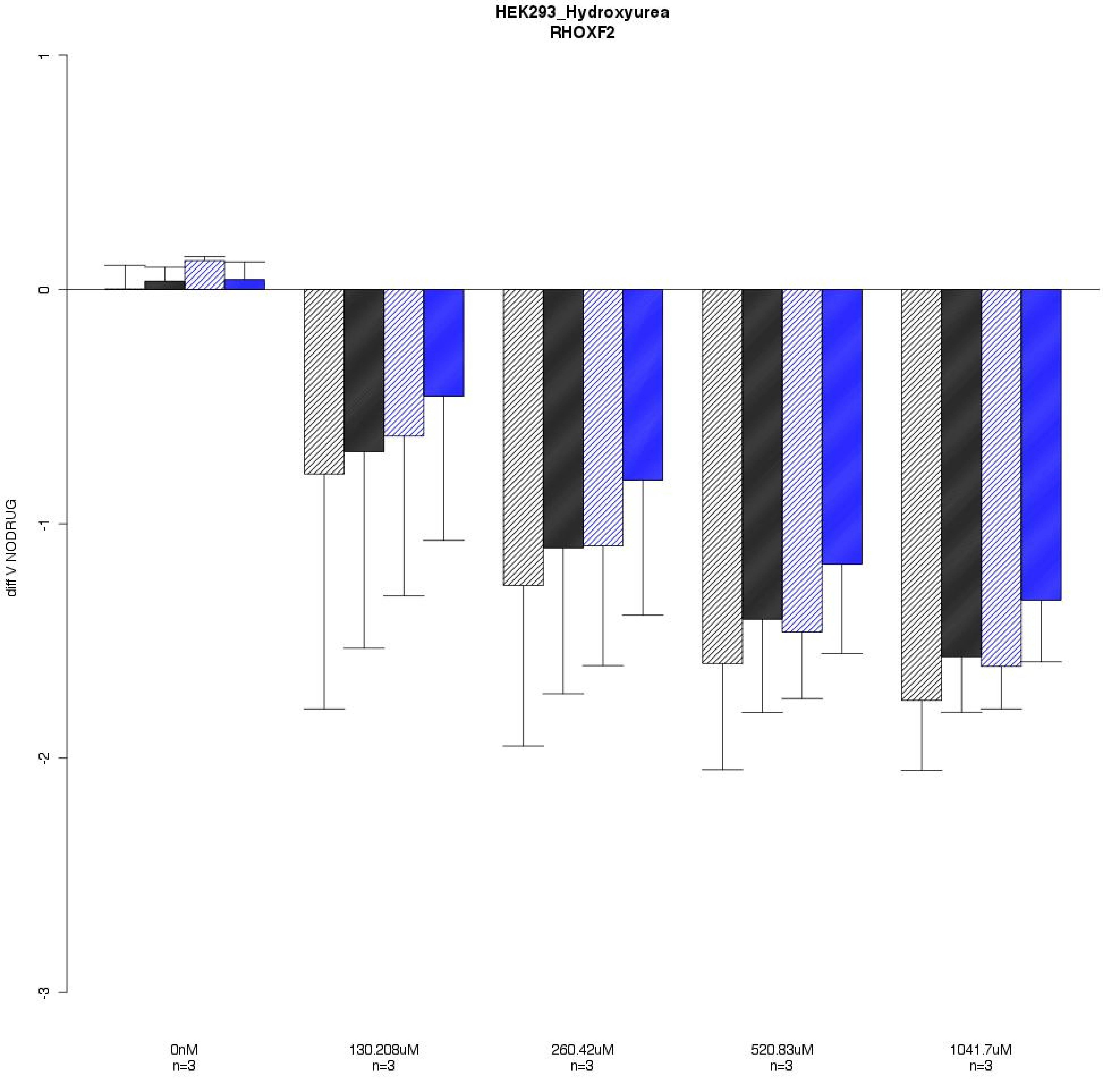

HEK293_Hydroxyurea
RHOXF2
diff V NODRUG
1
0
-1
-2
-3
0nM
n=3
130.208uM
n=3
260.42uM
n=3
520.83uM
n=3
1041.7uM
n=3

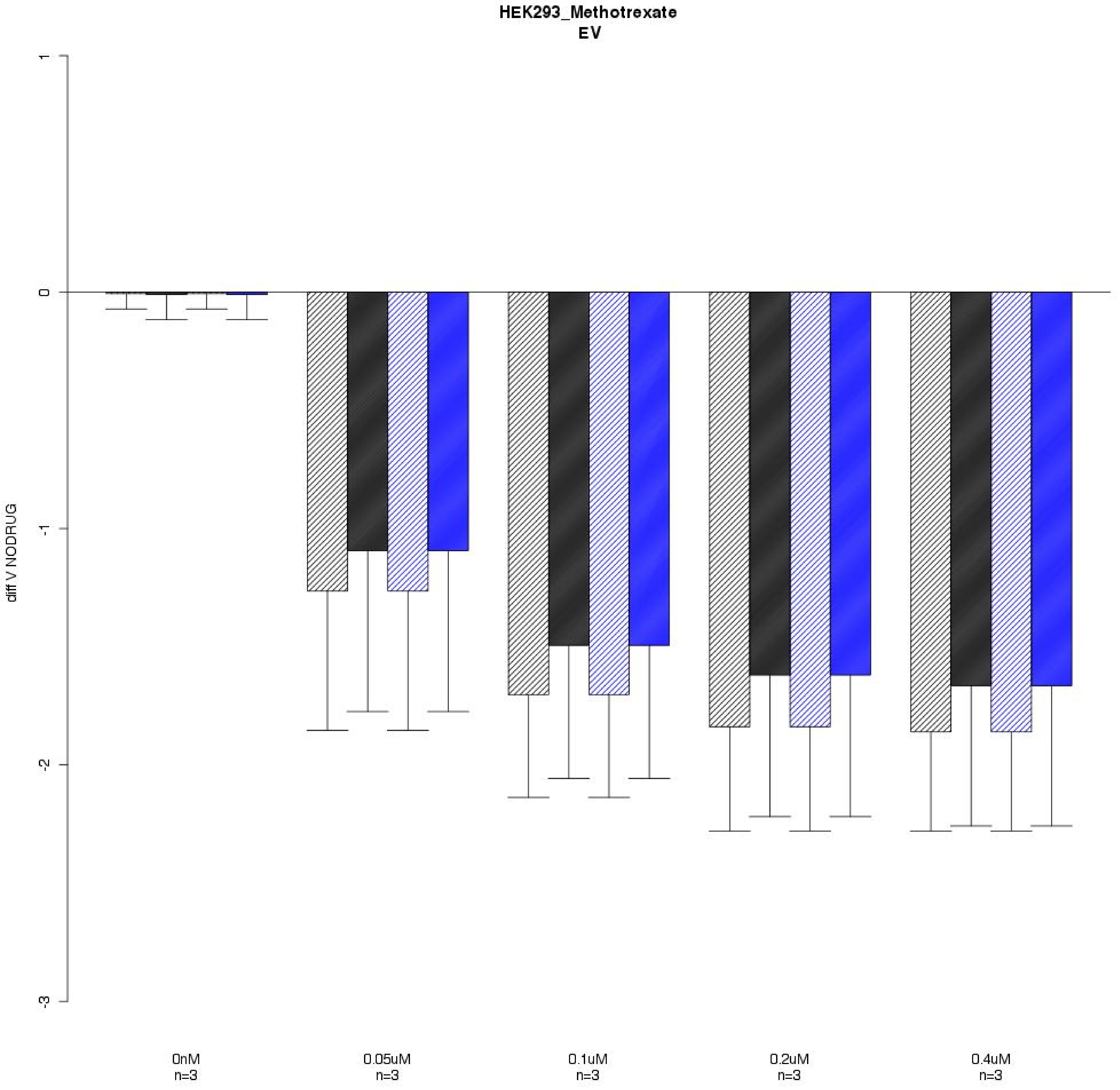

HEK293_Methotrexate
EV
diff V NODRUG
1
0
-1
-2
-3
0nM
n=3
0.05uM
n=3
0.1uM
n=3
0.2uM
n=3
0.4uM
n=3

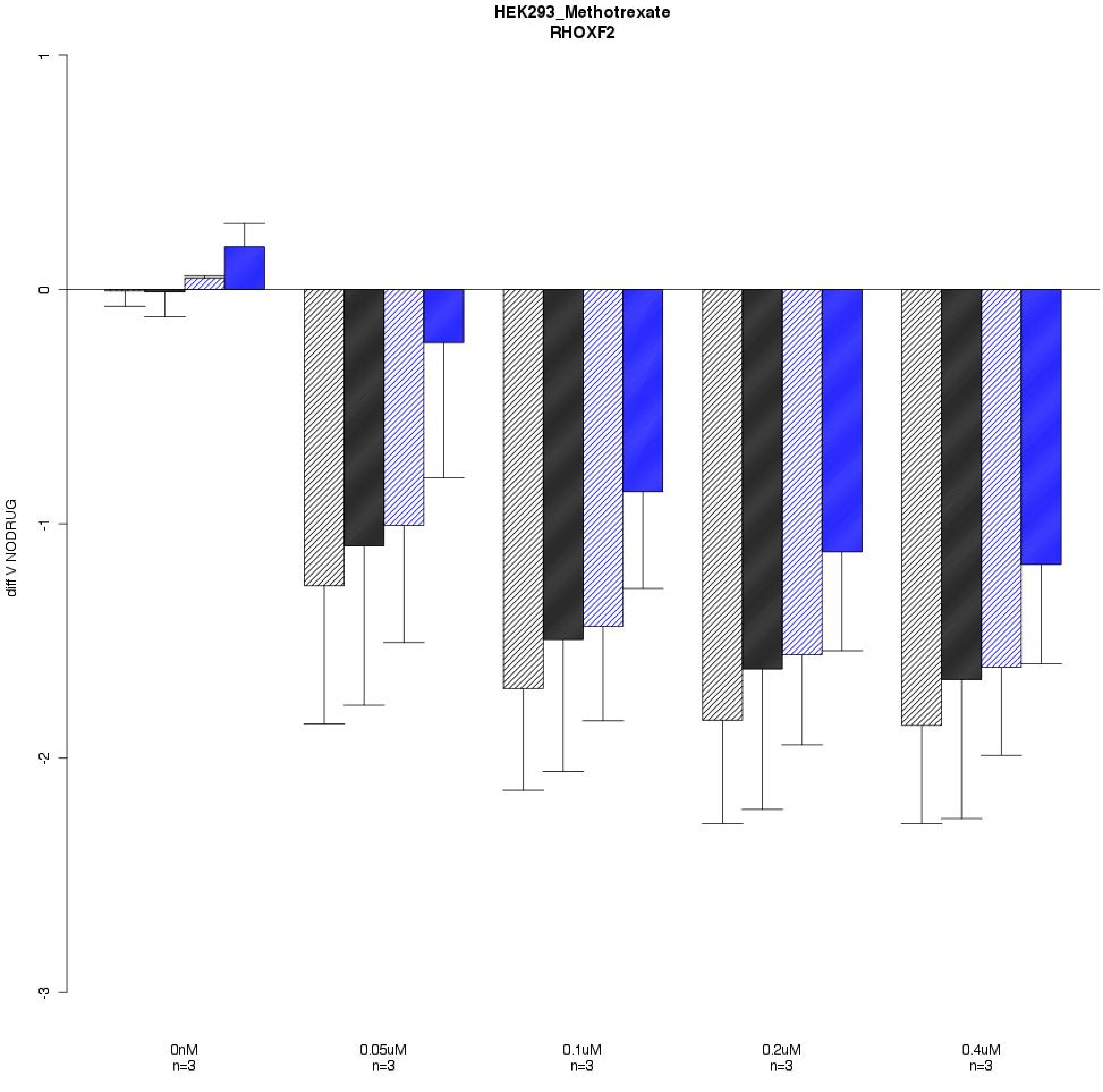

HEK293_Methotrexate
RHOXF2
diff V NODRUG
1
0
-1
-2
-3
0nM
n=3
0.05uM
n=3
0.1uM
n=3
0.2uM
n=3
0.4uM
n=3

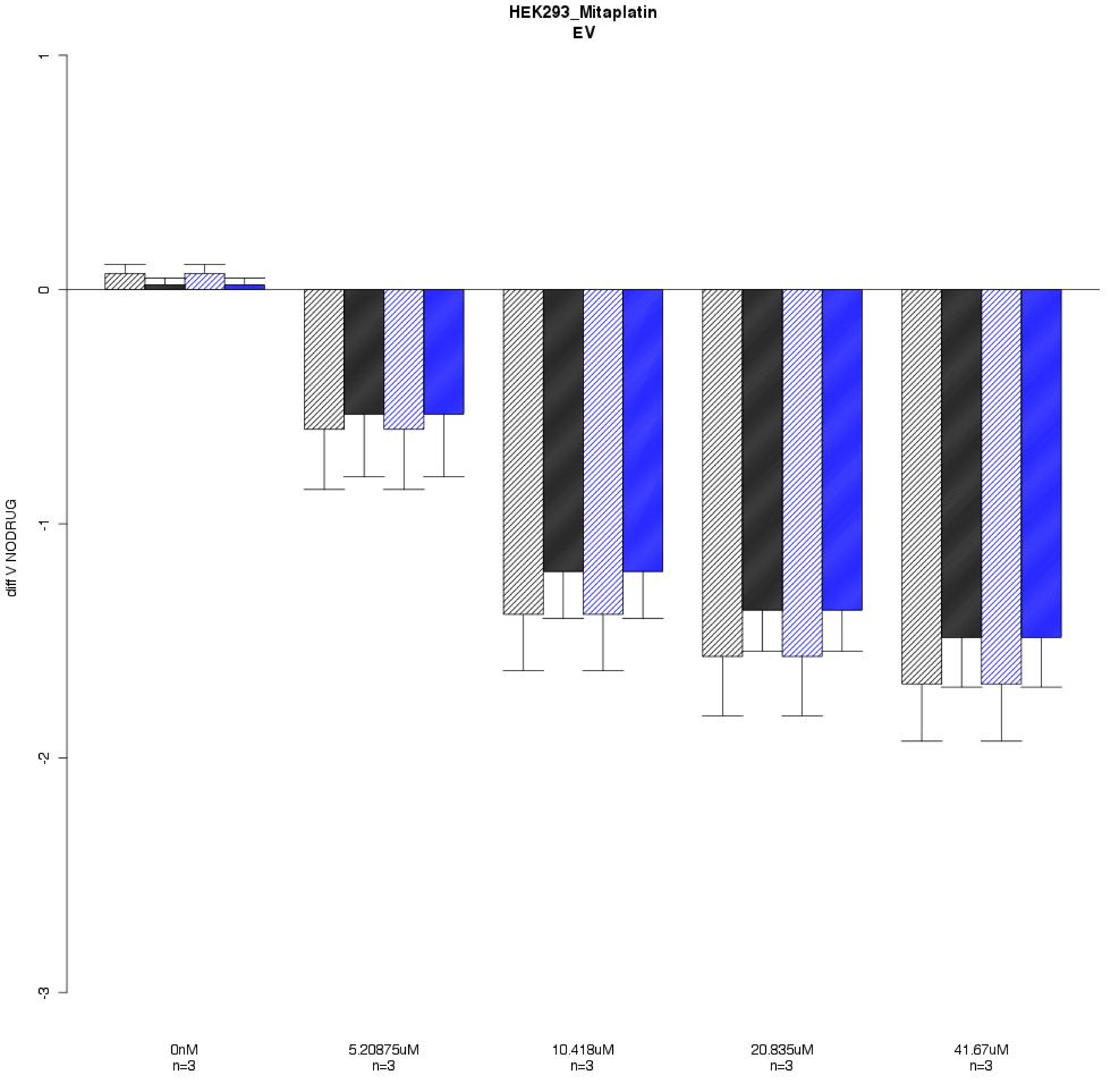
HEK293_Mitaplatin
EV
diff V NODRUG
1
0
-1
-2
-3
0nM
n=3
5.20875uM
n=3
10.418uM
n=3
20.835uM
n=3
41.67uM
n=3

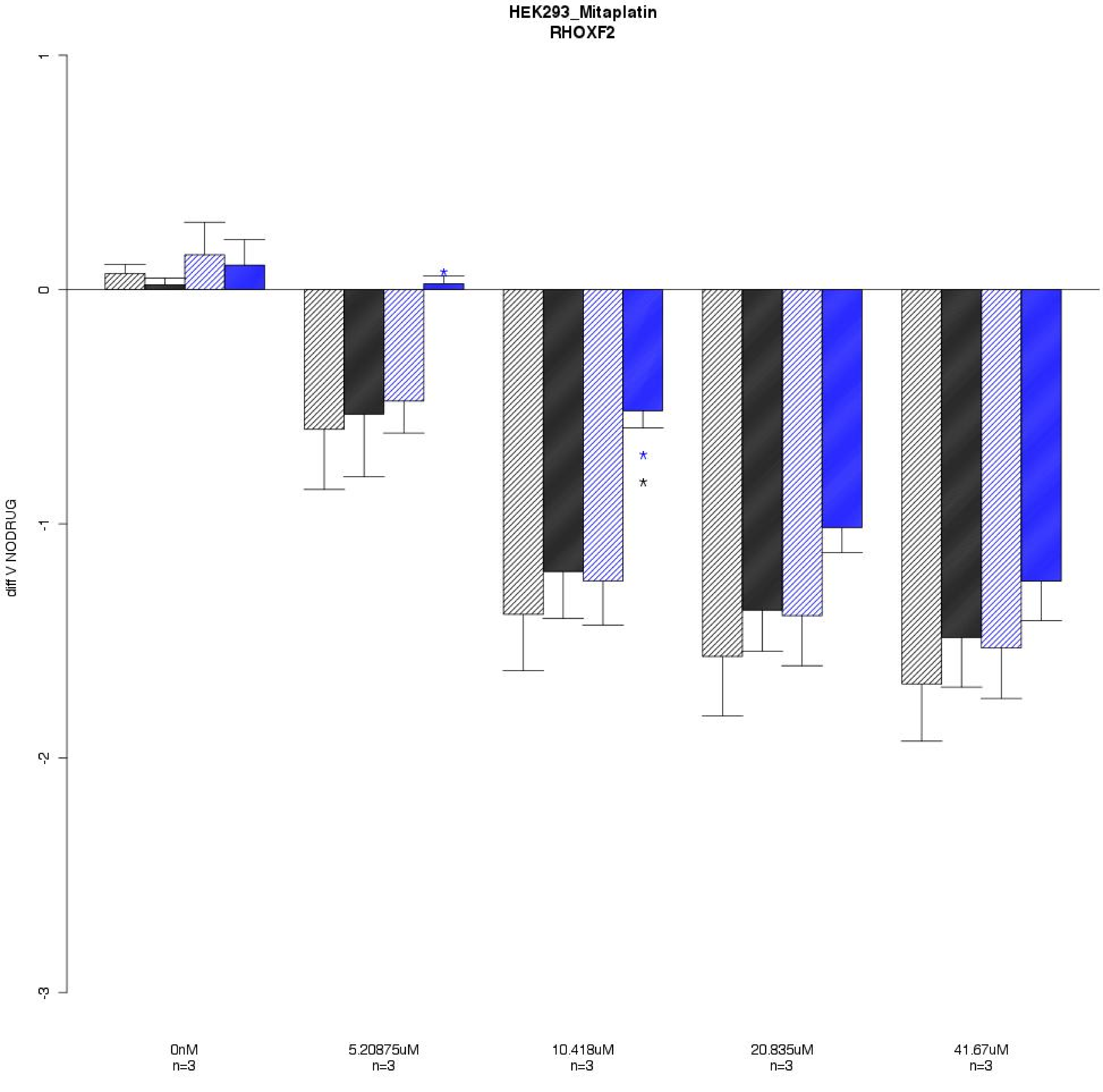
HEK293_Mitaplatin
RHOXF2
diff V NODRUG
1
0
-1
-2
-3
*
*
*
0nM
n=3
5.20875uM
n=3
10.418uM
n=3
20.835uM
n=3
41.67uM
n=3

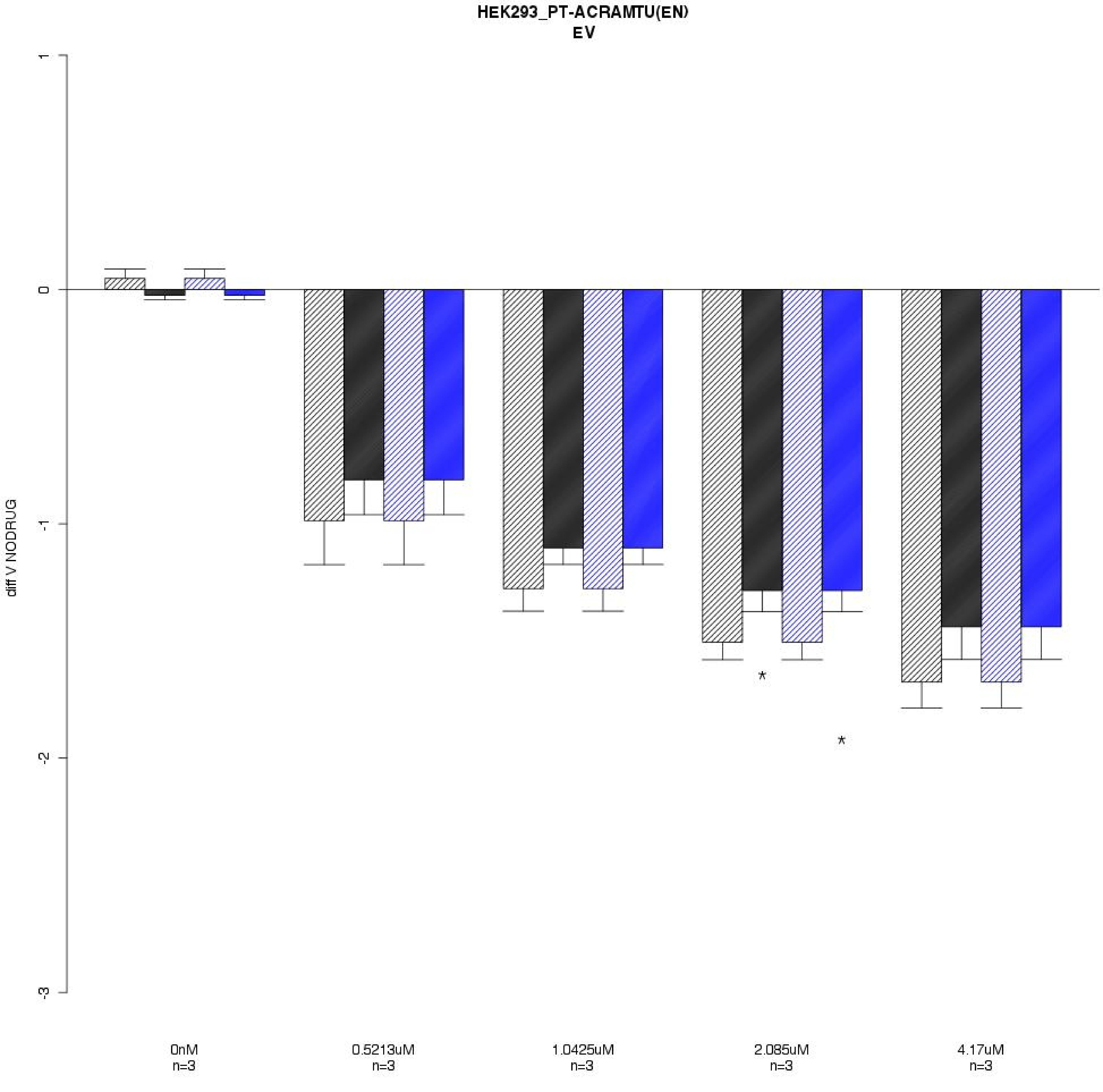
HEK293_PT-ACRAMTU(EN)
EV
diff V NODRUG
1
0
-1
-2
-3
*
*
0nM
n=3
0.5213uM
n=3
1.0425uM
n=3
2.085uM
n=3
4.17uM
n=3

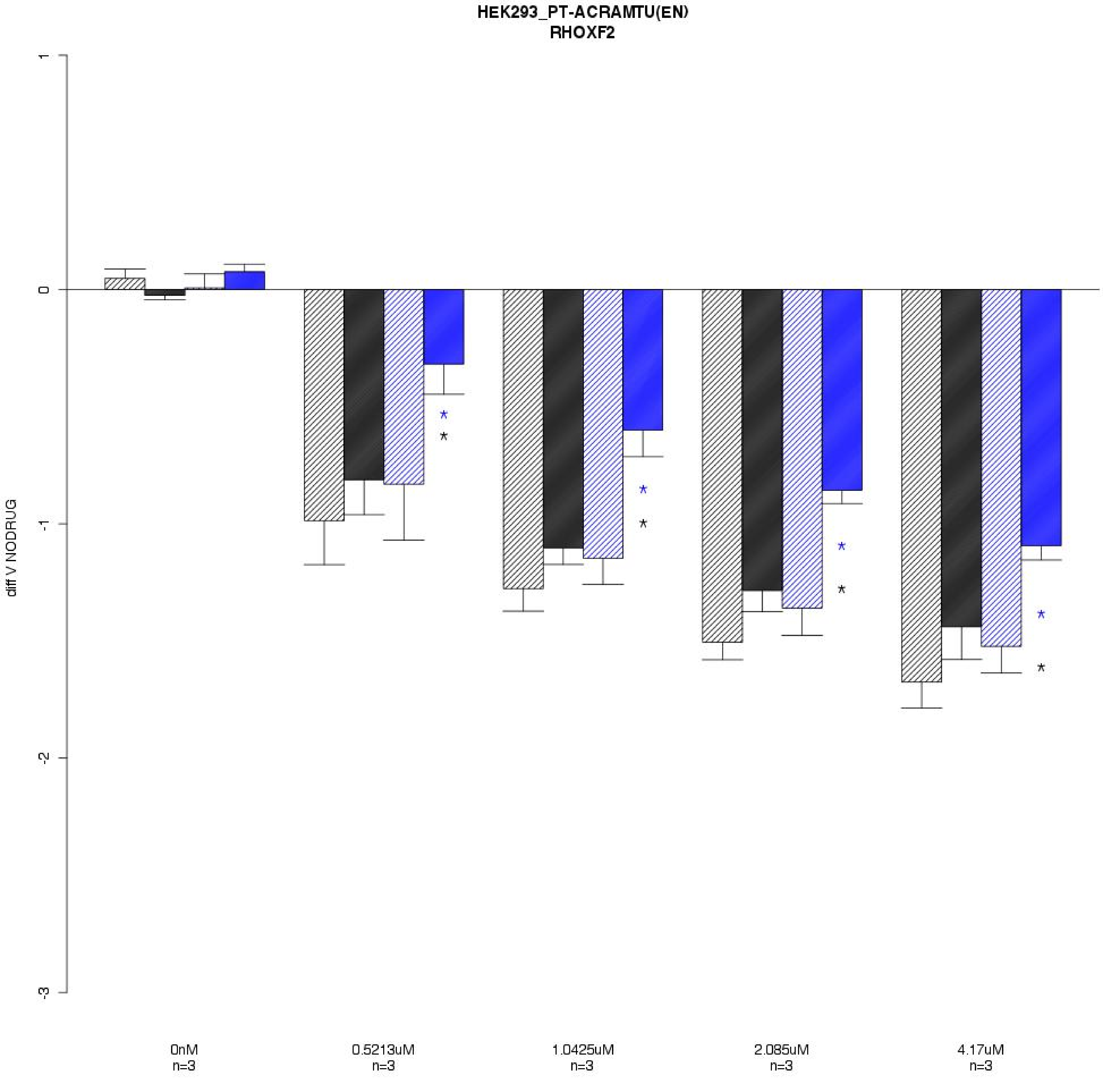
HEK293_PT-ACRAMTU(EN)
RHOXF2
1
0
-1
-2
-3
diff V NODRUG
*
*
0nM
n=3
0.5213uM
n=3
1.0425uM
n=3
2.085uM
n=3
4.17uM
n=3

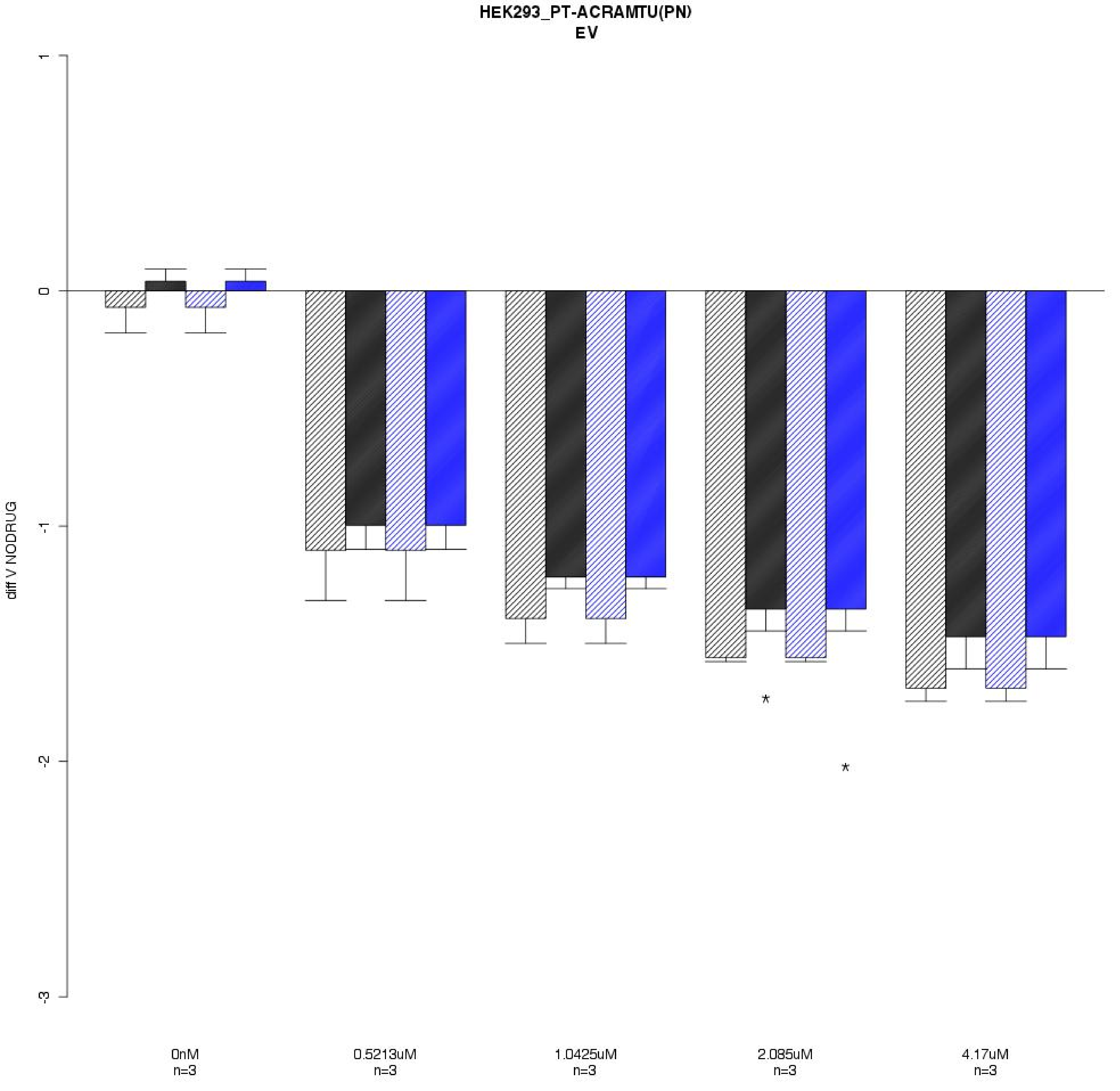
HEK293_PT-ACRAMTU(PN)
EV
diff V NODRUG
1
0
-1
-2
-3
0nM
n=3
0.5213uM
n=3
1.0425uM
n=3
2.085uM
n=3
4.17uM
n=3
*
*

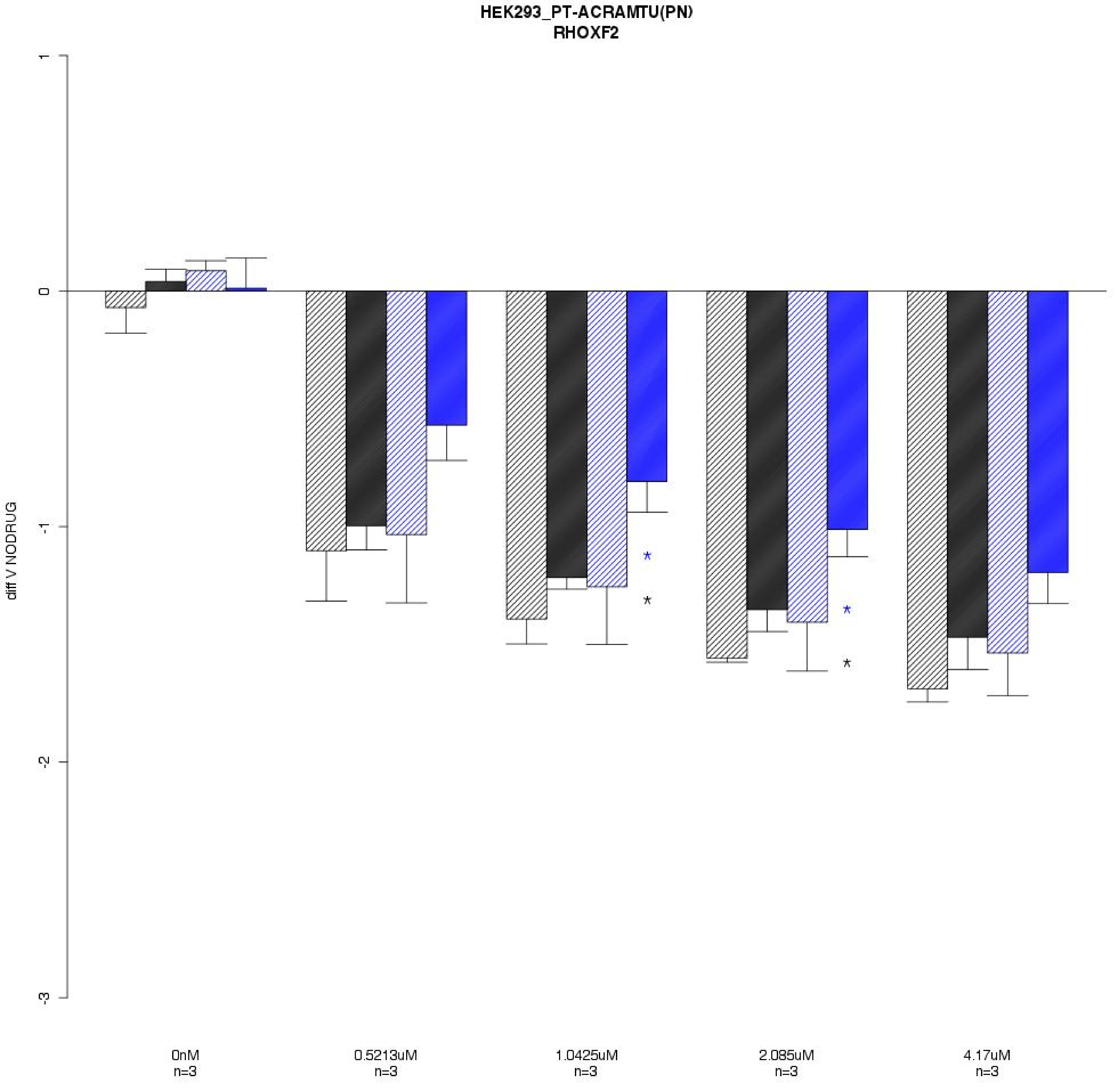

HEK293_PT-ACRAMTU(PN)
RHOXF2
diff V NODRUG
1
0
-1
-2
-3
*
*
*
*
0nM
n=3
0.5213uM
n=3
1.0425uM
n=3
2.085uM
n=3
4.17uM
n=3

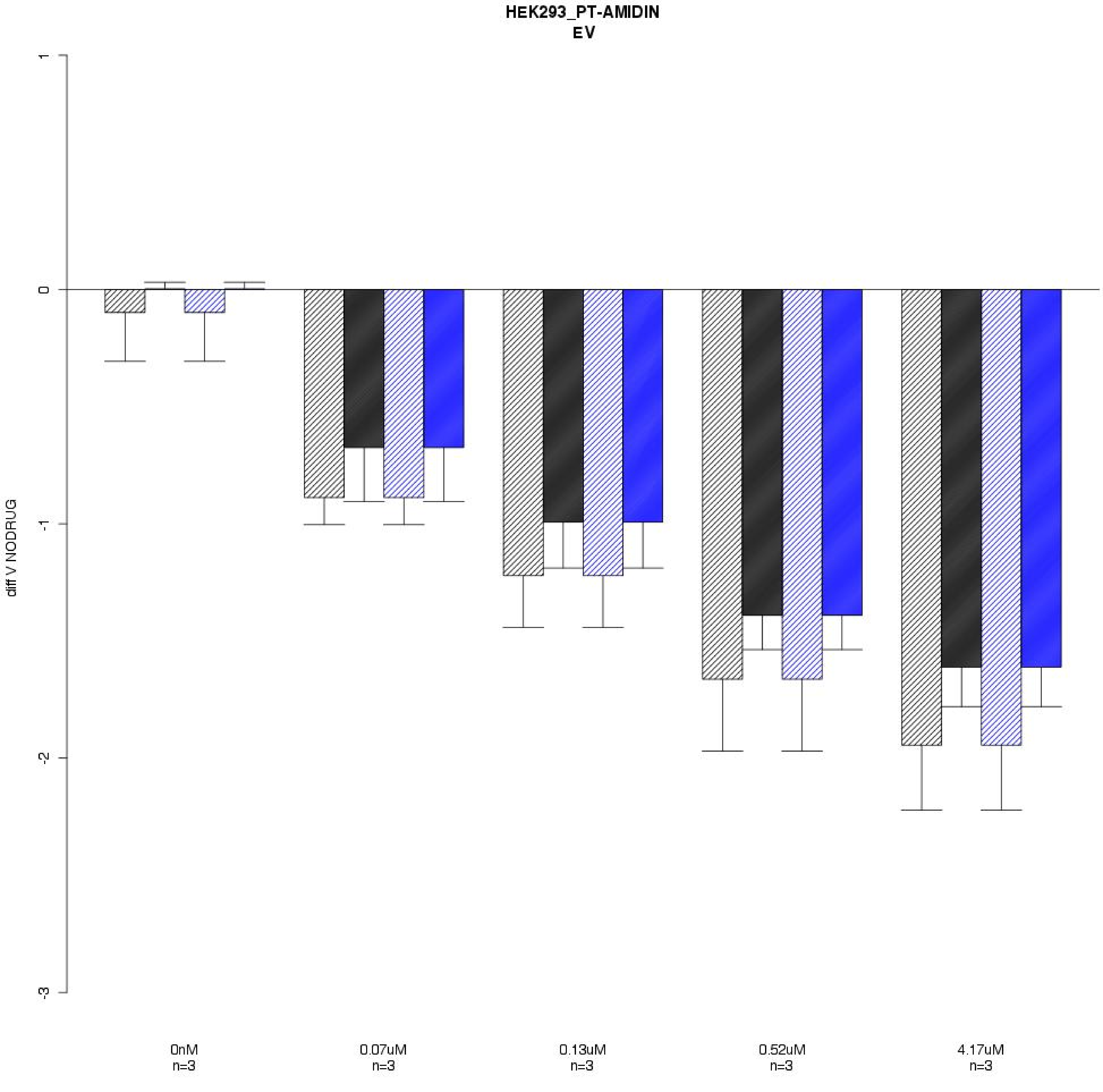
HEK293_PT-AMIDIN
EV
1
0
-1
-2
-3
diff V NODRUG
0nM
n=3
0.07uM
n=3
0.13uM
n=3
0.52uM
n=3
4.17uM
n=3

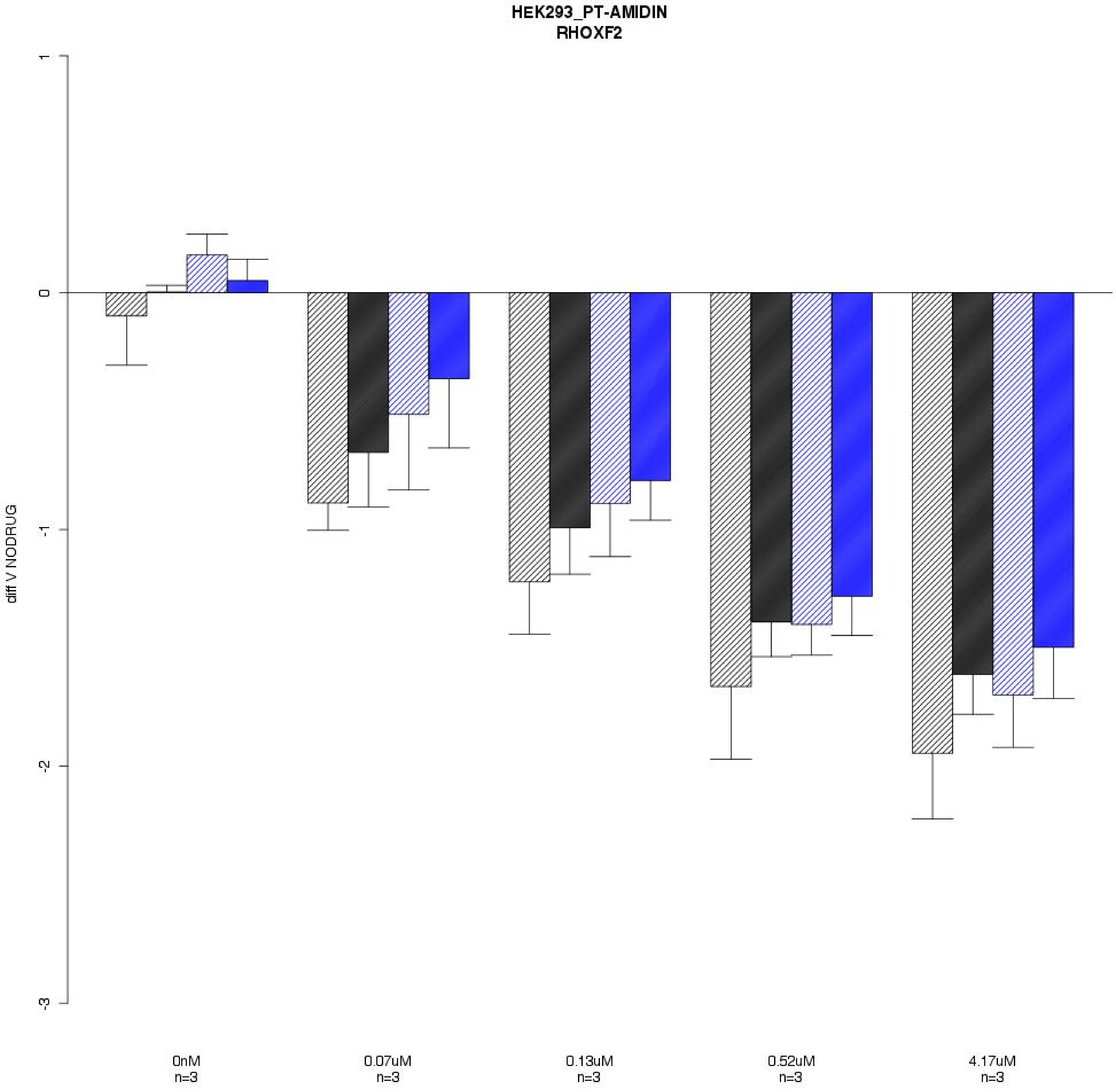
HEK293_PT-AMIDIN
RHOXF2
diff V NODRUG
1
0
-1
-2
-3
0nM
n=3
0.07uM
n=3
0.13uM
n=3
0.52uM
n=3
4.17uM
n=3

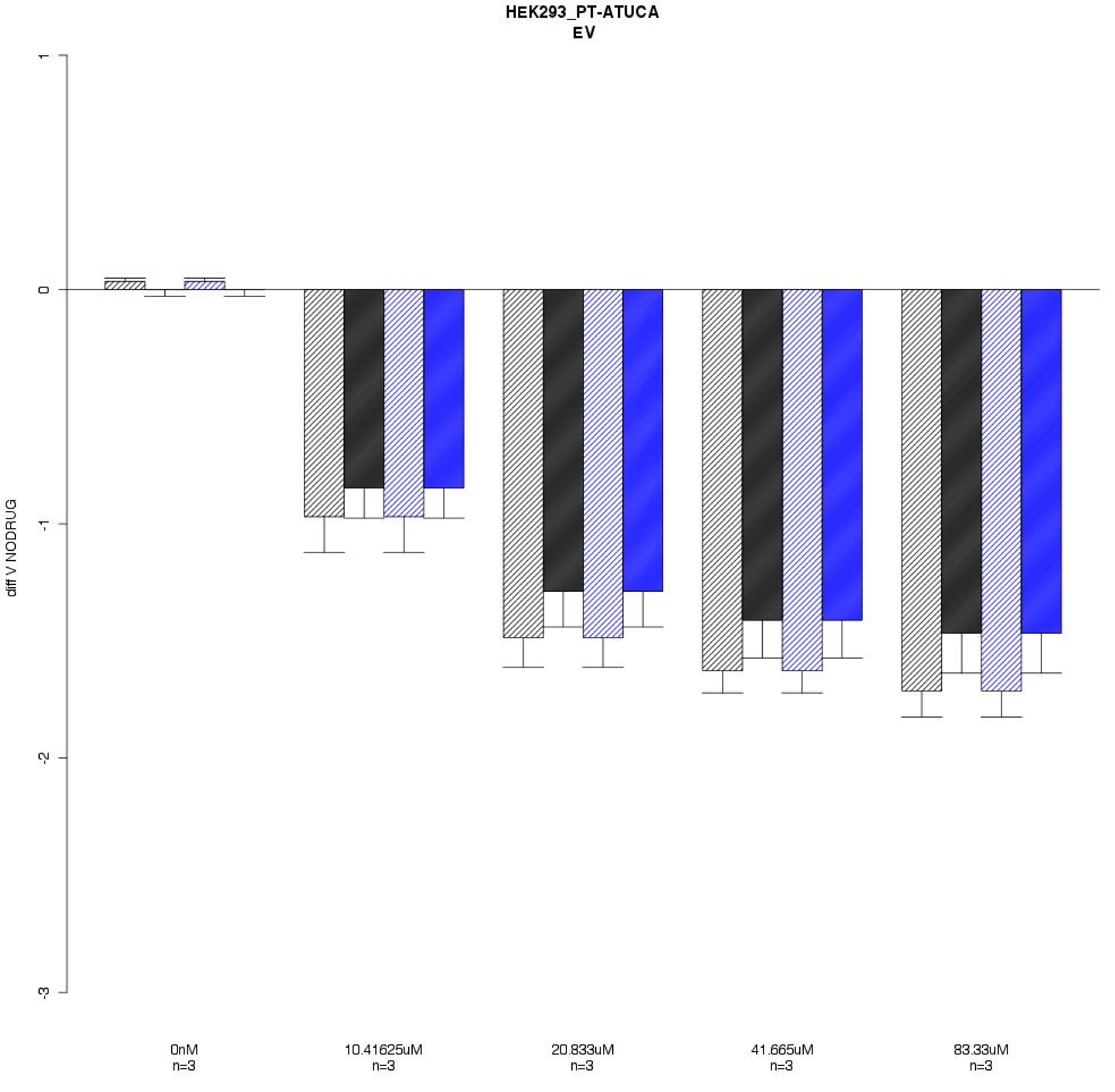
HEK293_PT-ATUCA
EV
1
0
-1
-2
-3
diff V NODRUG
0nM
n=3
10.41625uM
n=3
20.833uM
n=3
41.665uM
n=3
83.33uM
n=3

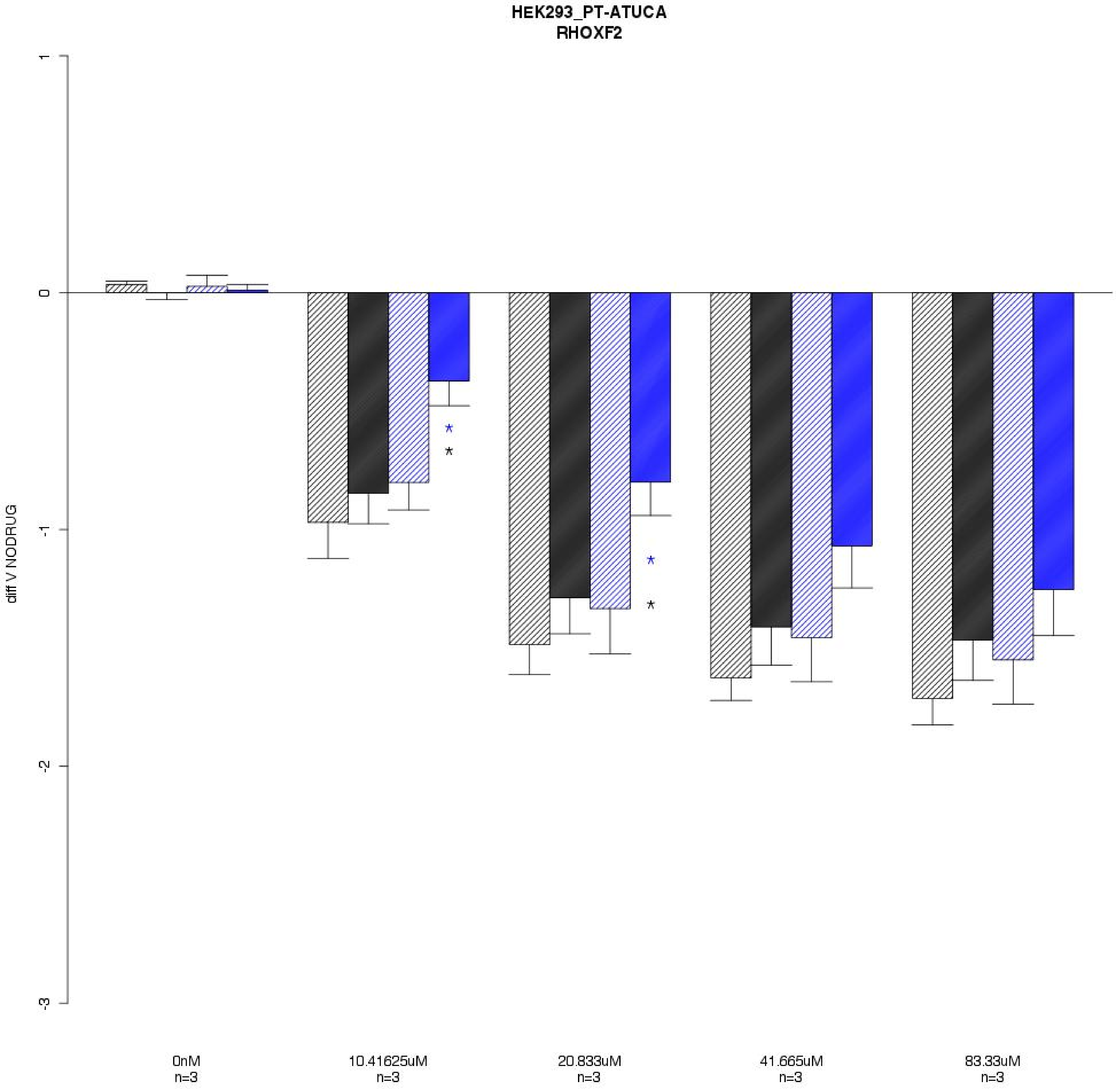
HEK293_PT-ATUCA
RHOXF2
diff V NODRUG
1
0
-1
-2
-3
0nM
n=3
10.41625uM
n=3
20.833uM
n=3
41.665uM
n=3
83.33uM
n=3

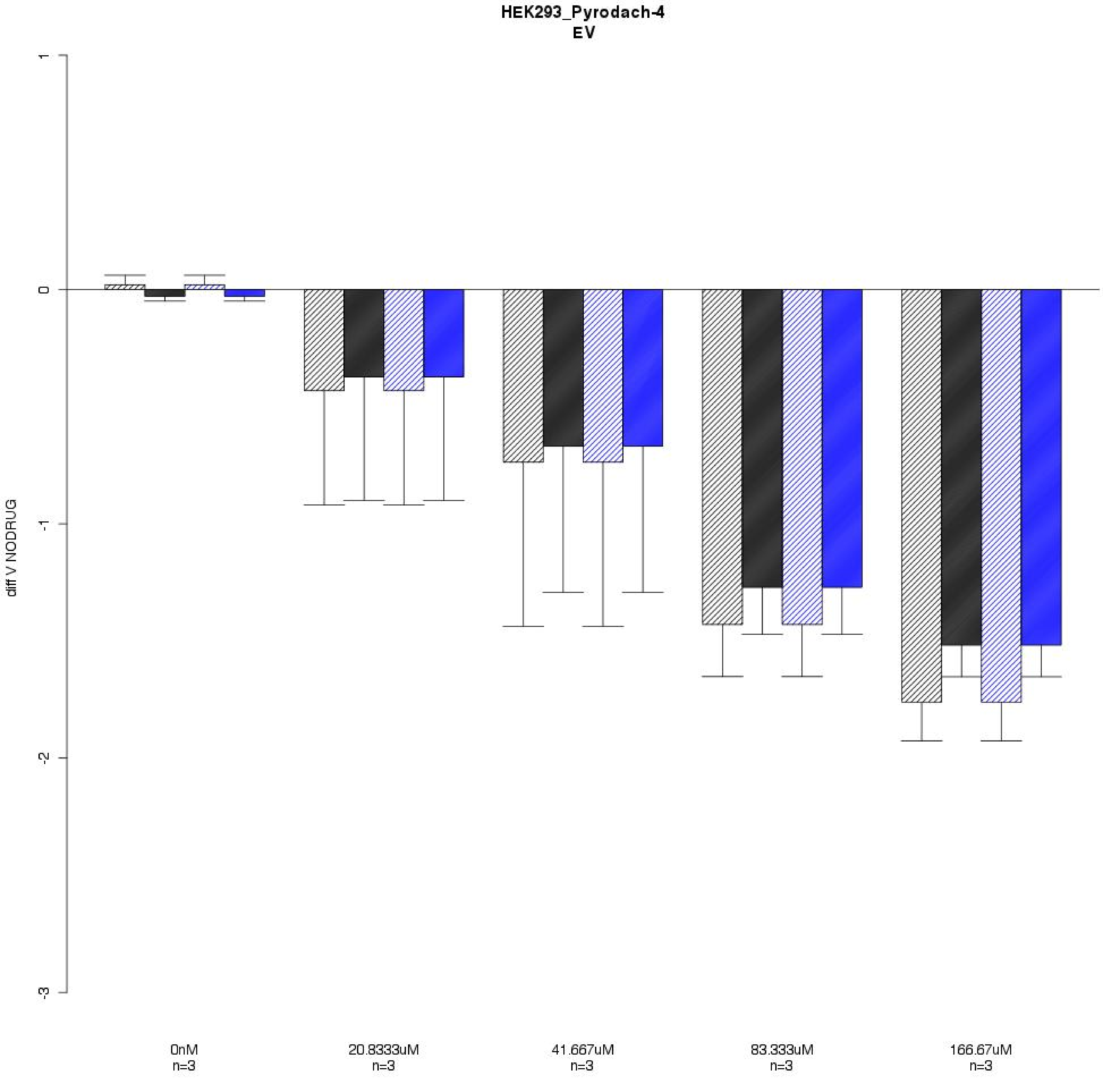
HEK293_Pyrodach-4
EV
diff V NODRUG
1
0
-1
-2
-3
0nM
n=3
20.8333uM
n=3
41.667uM
n=3
83.333uM
n=3
166.67uM
n=3

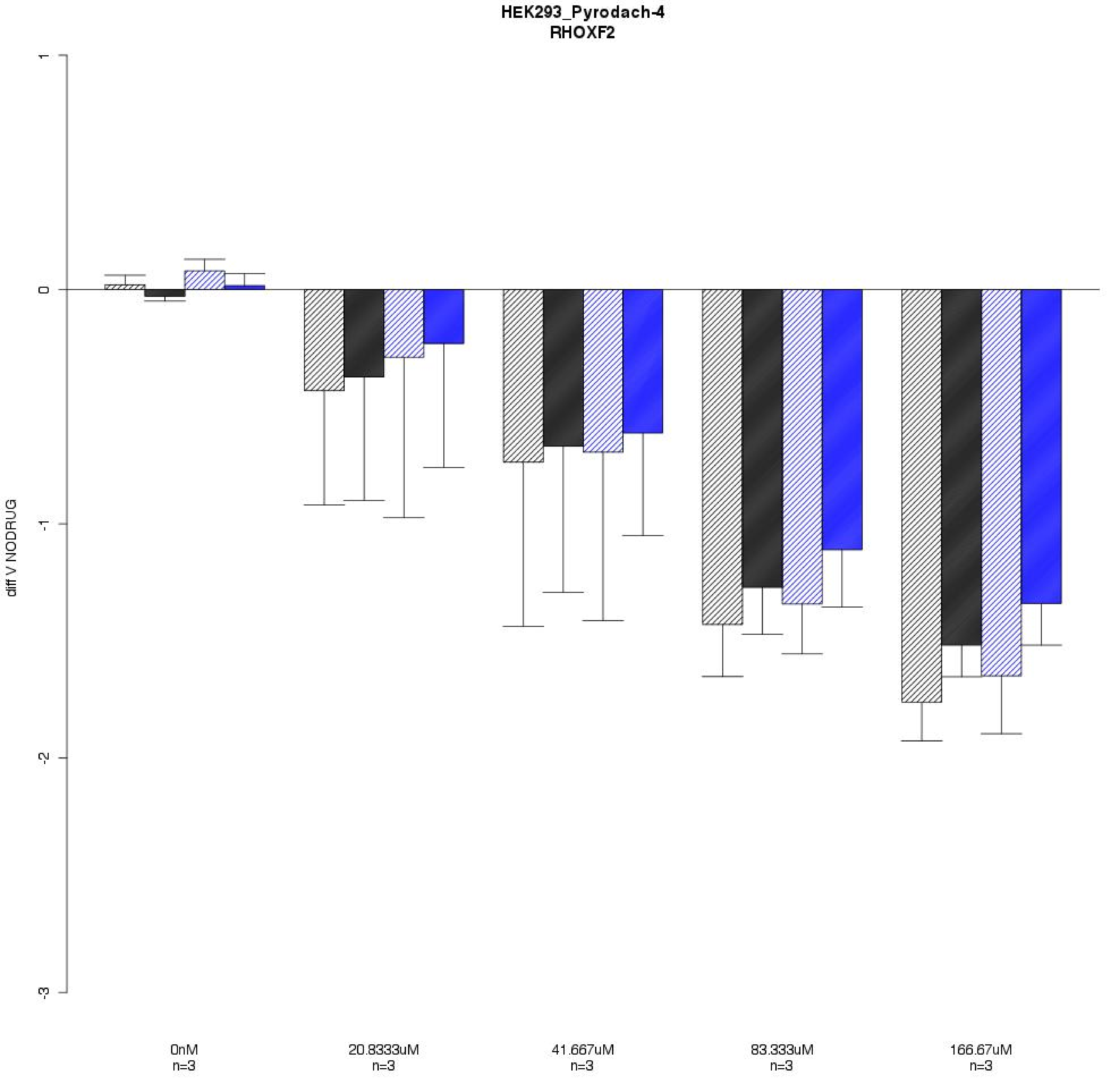
HEK293_Pyrodach-4
RHOXF2
diff V NODRUG
1
0
-1
-2
-3
0nM
n=3
20.8333uM
n=3
41.667uM
n=3
83.333uM
n=3
166.67uM
n=3
