# Supplementary material for: A genome scale overexpression screen to reveal drug activity in human cells
Source: Genome Med. 2014 Apr 29;6(4):32. doi: 10.1186/gm549 (PMC4062067; doi:10.1186/gm549)
Supplement: Additional file 10 — RHOXF2 overexpression rescued cisplatin toxicity in HEK293_M2 and MCF7_M2 but not A549_M2 cells. Stable RHOXF2 cells were cultured in the presence of an increasing concentration of cisplatin and their growth was compared to the stable cells with the empty vector PB-TGcMV-Neo. The effect of RHOXF2 expression on cell viability was measured three days after drug exposure and compared to cell cultured in the absence of drug as a 100% viability control. Lines show the nonlinear fit of a variable-slope dose-response model for (a) MCF7_M2 cells, (b) A549_M2 cells and (c) HEK293_M2 cells as positive control; (d) resulting IC50s. Data analysis was performed using the 'drc' package in R. Experiments were done in triplicate. [file gm549-S10.pptx]

## Slide 1
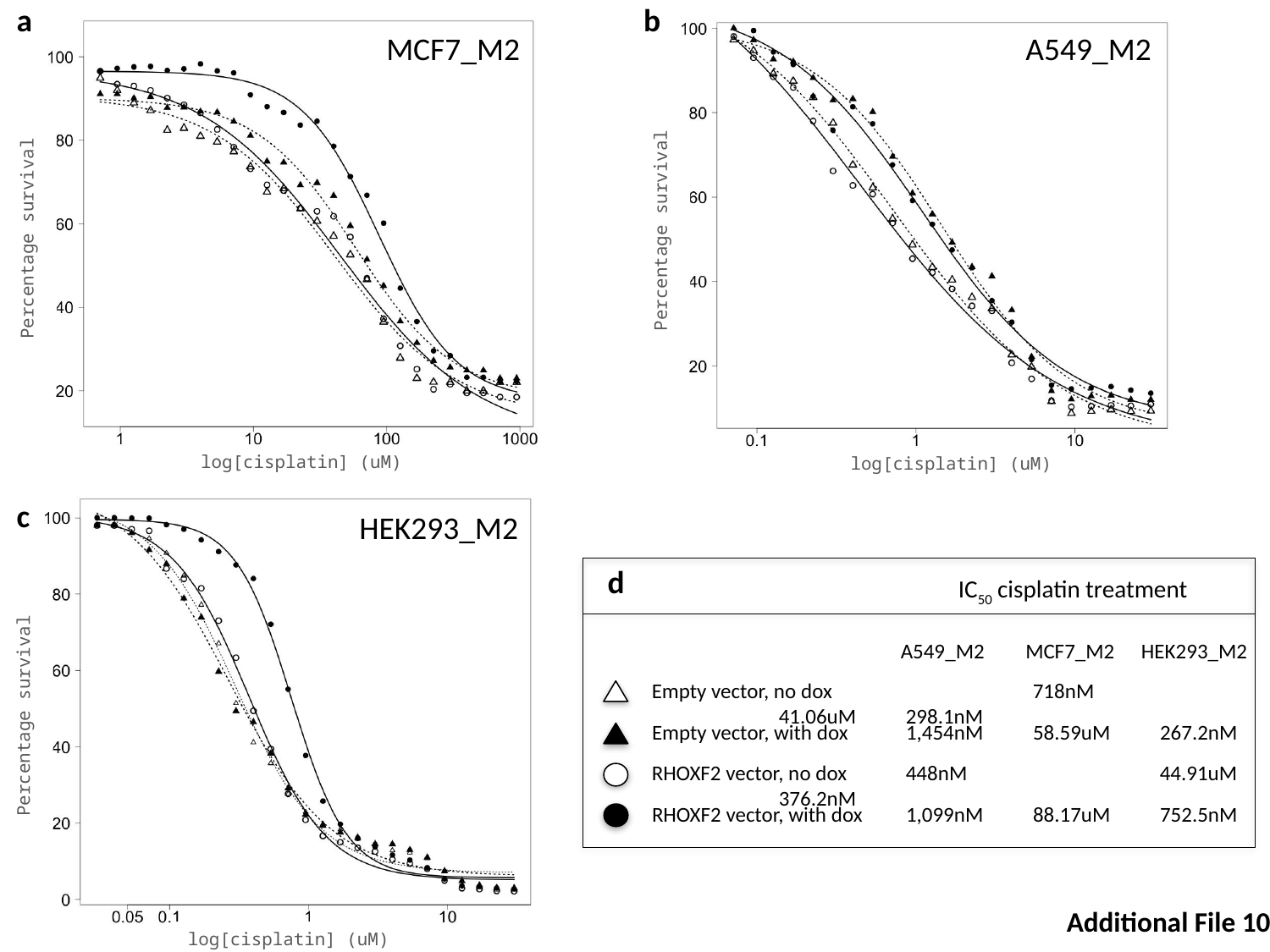

a
b
MCF7_M2
A549_M2
Percentage survival
Percentage survival
log[cisplatin] (uM)
log[cisplatin] (uM)
c
HEK293_M2
d
IC50 cisplatin treatment
Percentage survival
A549_M2
MCF7_M2
HEK293_M2
Empty vector, no dox		718nM		41.06uM	298.1nM
Empty vector, with dox	1,454nM	58.59uM	267.2nM
RHOXF2 vector, no dox	448nM		44.91uM	376.2nM
RHOXF2 vector, with dox	1,099nM	88.17uM	752.5nM
Additional File 10
log[cisplatin] (uM)
