# Supplementary material for: A genome scale overexpression screen to reveal drug activity in human cells
Source: Genome Med. 2014 Apr 29;6(4):32. doi: 10.1186/gm549 (PMC4062067; doi:10.1186/gm549)
Supplement: Additional file 12 — RHOXF2 knocked-down in HPAC, NCI-H1299 and SW1353 cells does not modulate cisplatin and mitomycin C sensitivities. (a-g) Dose-response curves for HPAC (a,b), NCI-H1299 (c,d) and SW1353 (f,g) were established after 2 days of exposure to cisplatin (a,c,f) or mitomycin C (b,d,g). Percentage of growth inhibition was calculated by comparing the number of cells treated with drug to the number of cells cultured in media with DMSO as control. Error bars represent standard error of the mean (n = 4). RHOXF2 depletion in NCI-H1299 (e) and SW1353 cells (h). Total cell extracts for each cell line were used to detect the presence of RHOXF2 by western blotting. Expression level was compared to the alpha tubulin as loading control. [file gm549-S12.pptx]

## Slide 1
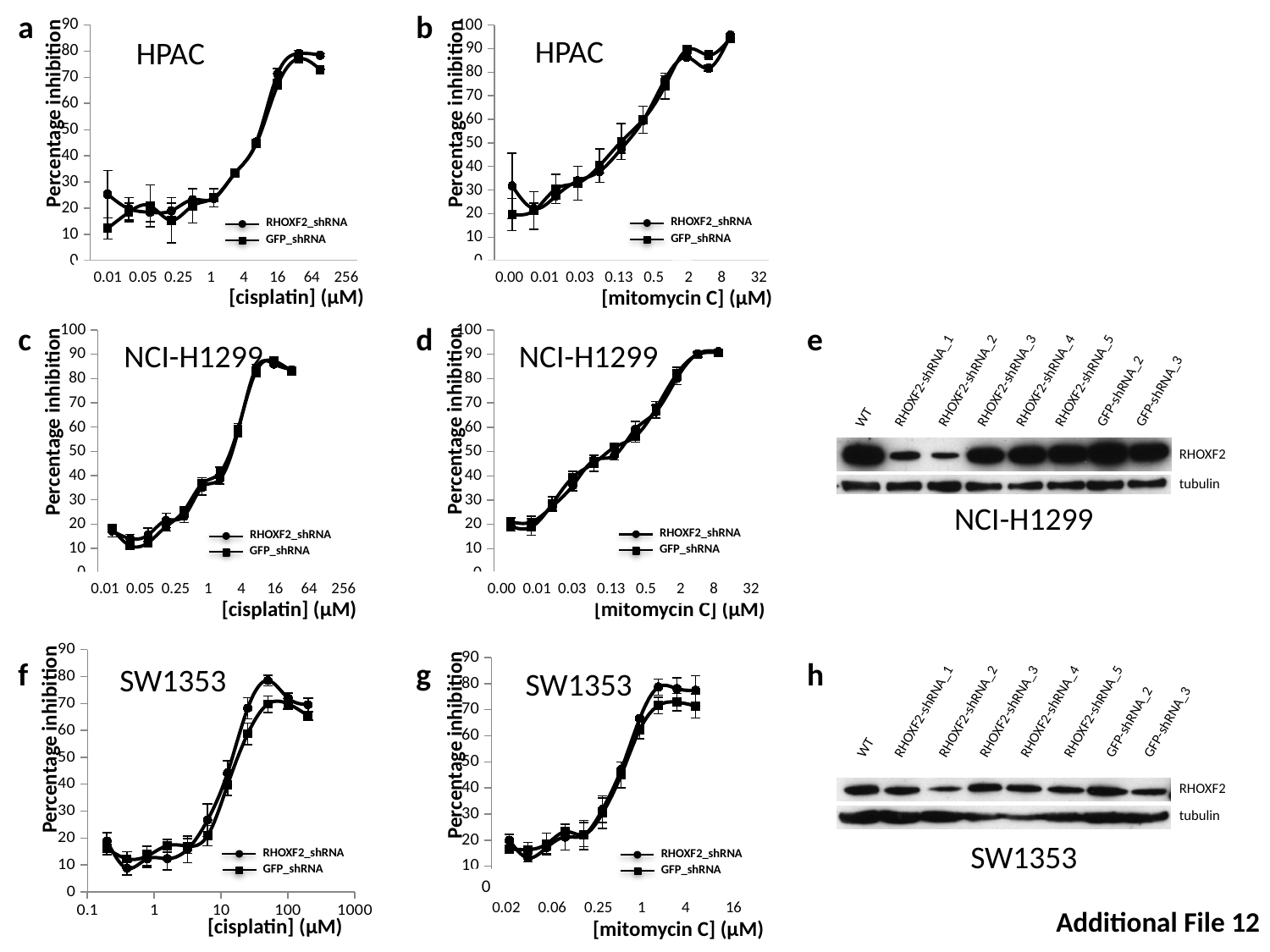

a
b
### Chart
| Category | Cisplatin inhibition RHOXF2-shRNA_2 | Cisplatin inhibition RHOXF2-GFP_2 |
|---|---|---|
### Chart
| Category | Mitomycin C inhibition RHOXF2-shRNA_2 | Mitomycin C inhibition RHOXF2-GFP_3 |
|---|---|---|HPAC
HPAC
Percentage inhibition
Percentage inhibition
RHOXF2_shRNA
GFP_shRNA
RHOXF2_shRNA
GFP_shRNA
 0.00 0.01 0.03 0.13 0.5
2 8 32
 0.01 0.05 0.25 1 4 16 64 256
[cisplatin] (μM)
[mitomycin C] (μM)
c
d
e
### Chart
| Category | Cisplatin inhibition RHOXF2-shRNA_2 | Cisplatin inhibition RHOXF2-GFP_3 |
|---|---|---|
### Chart
| Category | Mitomycin C inhibition RHOXF2-shRNA_2 | Cisplatin inhibition RHOXF2-GFP_3 |
|---|---|---|NCI-H1299
NCI-H1299
RHOXF2-shRNA_1
RHOXF2-shRNA_2
RHOXF2-shRNA_3
RHOXF2-shRNA_4
RHOXF2-shRNA_5
GFP-shRNA_2
GFP-shRNA_3
Percentage inhibition
WT
Percentage inhibition
RHOXF2
tubulin
NCI-H1299
RHOXF2_shRNA
GFP_shRNA
RHOXF2_shRNA
GFP_shRNA
 0.00 0.01 0.03 0.13 0.5
2 8 32
 0.01 0.05 0.25 1 4 16 64 256
[cisplatin] (μM)
[mitomycin C] (μM)
### Chart
| Category | Cisplatin inhibition RHOXF2-shRNA_2 | Cisplatin inhibition RHOXF2-GFP_2 |
|---|---|---|f
g
### Chart
| Category | Mitomycin C inhibition RHOXF2-shRNA_2 | Cisplatin inhibition RHOXF2-GFP_2 |
|---|---|---|h
SW1353
SW1353
RHOXF2-shRNA_1
RHOXF2-shRNA_2
RHOXF2-shRNA_3
RHOXF2-shRNA_4
RHOXF2-shRNA_5
GFP-shRNA_2
GFP-shRNA_3
Percentage inhibition
Percentage inhibition
WT
RHOXF2
tubulin
SW1353
RHOXF2_shRNA
GFP_shRNA
RHOXF2_shRNA
GFP_shRNA
0
 0.02 0.06 0.25 1 4 16
Additional File 12
[cisplatin] (μM)
[mitomycin C] (μM)
