# Supplementary material for: A genome scale overexpression screen to reveal drug activity in human cells
Source: Genome Med. 2014 Apr 29;6(4):32. doi: 10.1186/gm549 (PMC4062067; doi:10.1186/gm549)
Supplement: Additional file 13 — RHOXF2 knocked down in K562 cells. (a,b) Cell lines in which RHOXF2 and GFP were knocked down were grown in the presence of 35 μM cisplatin (a) or 7 μM of mitomycin C (b) for 2 days. Total cell extracts for each cell line were used to detect the presence of RHOXF2 by western blotting. Expression level was compared to alpha tubulin as loading control. (c) Total cell extracts from a RHOXF2-expressing cell line and a cell line with GFP knocked down but overexpressing RHOXF2 were used to detect the presence of RHOXF2 by western blotting. [file gm549-S13.pptx]

## Slide 1
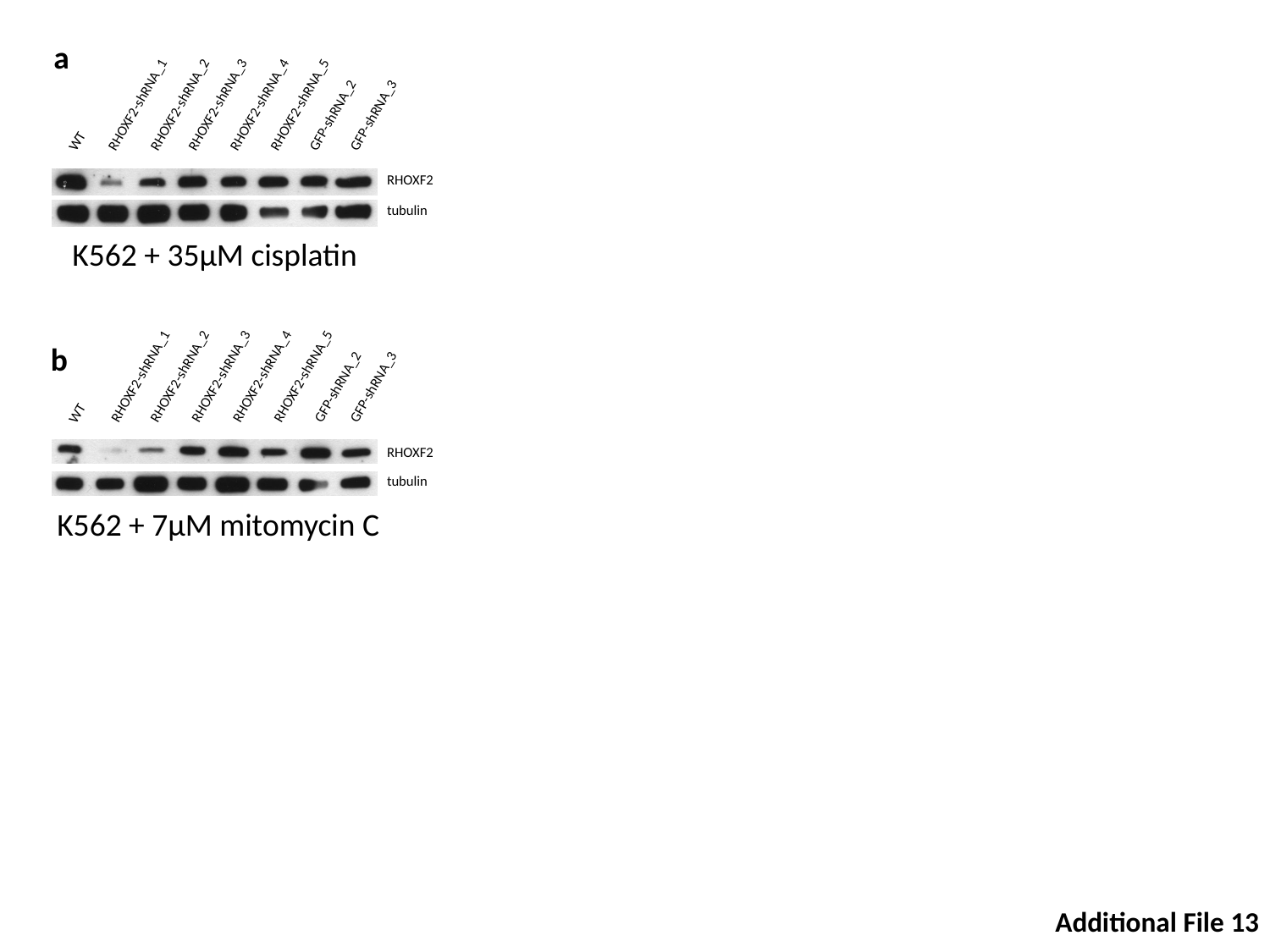

a
RHOXF2-shRNA_1
RHOXF2-shRNA_2
RHOXF2-shRNA_3
RHOXF2-shRNA_4
RHOXF2-shRNA_5
GFP-shRNA_2
GFP-shRNA_3
WT
RHOXF2
tubulin
K562 + 35μM cisplatin
b
RHOXF2-shRNA_1
RHOXF2-shRNA_2
RHOXF2-shRNA_3
RHOXF2-shRNA_4
RHOXF2-shRNA_5
GFP-shRNA_2
GFP-shRNA_3
WT
RHOXF2
tubulin
K562 + 7μM mitomycin C
Additional File 13
